# Identification of 1*H*-pyrazolo[3,4-b]pyridine derivatives as novel and potent TBK1 inhibitors: design, synthesis, biological evaluation, and molecular docking study

**DOI:** 10.1080/14756366.2022.2076674

**Published:** 2022-05-19

**Authors:** Yin Sun, Haotian Tang, Xiaoyan Wang, Fang Feng, Tiantian Fan, Dongmei Zhao, Bing Xiong, Hua Xie, Tongchao Liu

**Affiliations:** a Key Laboratory of Structure-Based Drug Design and Discovery of Ministry of Education, Shenyang Pharmaceutical University, Shenyang, P. R. China; b Shanghai Institute of Materia Medica, Chinese Academy of Sciences, Shanghai, P. R. China; c University of Chinese Academy of Sciences, Beijing, P. R. China; d Zhongshan Institute for Drug Discovery, Shanghai Institute of Materia Medica, Chinese Academy of Sciences, Shanghai, P. R. China

**Keywords:** TBK1 inhibitors, 1*H*-pyrazolo[3,4-b]pyridine, structure–activity relationships (SARs), immune response, cancer therapy

## Abstract

TANK-binding kinase 1 (TBK1), a noncanonical member of the inhibitor-kappaB kinases (IKKs) family, plays a vital role in coordinating the signalling pathways of innate immunity, involving in the process of neuroinflammation, autophagy, and oncogenesis. In current study, based on rational drug design strategy, we discovered a series of 1*H*-pyrazolo[3,4-b]pyridine derivatives as potent TBK1 inhibitors and dissected the structure–activity relationships (SARs). Through the several rounds of optimisation, compound **15y** stood out as a potent inhibitor on TBK1 with an IC_50_ value of 0.2 nM and also displayed good selectivity. The mRNA detection of TBK1 downstream genes showed that compound **15y** effectively inhibited TBK1 downstream IFN signalling in stimulated THP-1 and RAW264.7 cells. Meanwhile, compound **15y** exhibited a micromolar antiproliferation effect on A172, U87MG, A375, A2058, and Panc0504 cell lines. Together, current results provided a promising TBK1 inhibitor **15y** as lead compound for immune- and cancer-related drug discovery.

## 1. Introduction

The inhibitor-kappaB kinases (IKKs), a conserved serine/threonine kinases family, is involved in the metabolism, immune response, and tumourigenesis.^1–3^ The five members of the IKKs comprise three canonical kinases: IKKα, IKKβ, and IKKγ (NEMO), and two noncanonical ones: TANK-binding kinase 1 (TBK1) and IKKε.^4^ Except IKKγ possessing a nonenzymatic regulatory component, the other canonical and noncanonical IKK kinases share similar structure characteristics: an N-terminal (Ser/Thr) catalytic kinase domain (KD) containing the ATP-binding site followed closely a ubiquitin-like domain (ULD), a C-terminal domain (CTD) facilitating the formation of multi-enzyme complexes by mediating the binding of adaptor proteins and a helical scaffold dimerisation domain (SDD) maintaining structural integrity.^5^ Moreover, the KD of TBK1 presents 49% identity and 65% similarity to that of IKKε. Thus, in order to further exploring the biological function of TBK1, development of a highly selective TBK1 inhibitor has become an urgent need. The past years has witnessed an exponential increase in the number of researchers who try to develop a selective TBK1 inhibitor due to the importance of TBK1 in cell signalling pathways.

TBK1, also known as NF-κB-activating kinase (NAK) or T2K, which is widely expressed in all tissues, has emerged as a prospective therapeutic target, playing increasingly momentous roles in metabolic diseases, autoimmune diseases, and cancer.^3^^,^^6^^,^^7^ Several studies have found that TBK1 is positively regulated by RIG-I like receptors (RLRs), Toll-like receptors (TLRs) and the stimulator of interferon genes (STING) protein.^8–10^ The activation of TBK1 is essential for the production of type I interferon (IFN) *in vivo*. The activated TBK1 can regulate IFN signal by phosphorylating interferon regulatory factor 3/7 (IRF3/7) and then dimerising them into the nucleus, thus inducing the expression of pro-inflammatory and anti-viral genes. In addition to mediating the innate immune response, TBK1 also plays a critical role in cancer therapy. A growing number of studies have suggested that aberrant activation of TBK1 is closely associated to the occurrence and development of cancer, such as lung,^11^ breast,^12^ colon,^13^ bladder,^14^ glioblastoma,^6^^,^^15^ melanoma,^16^ and pancreas^17^^,^^18^ cancers. Knockdown experiments have identified that TBK1 is a synthetic lethal partner of oncogenic mutated oncogenic Kirsten rat sarcoma 2 viral oncogene homolog (KRAS).^19^ Although TBK1 has been proposed as a target of inflammatory, autoimmune and metabolic disorders as well as cancer, the related mechanisms remain unclear to large extent. Therefore, tool molecules need to be developed urgently for further revealing TBK1 biological functions involving the correlative network of cell signals of immune response and cancer.

**Figure 1.**
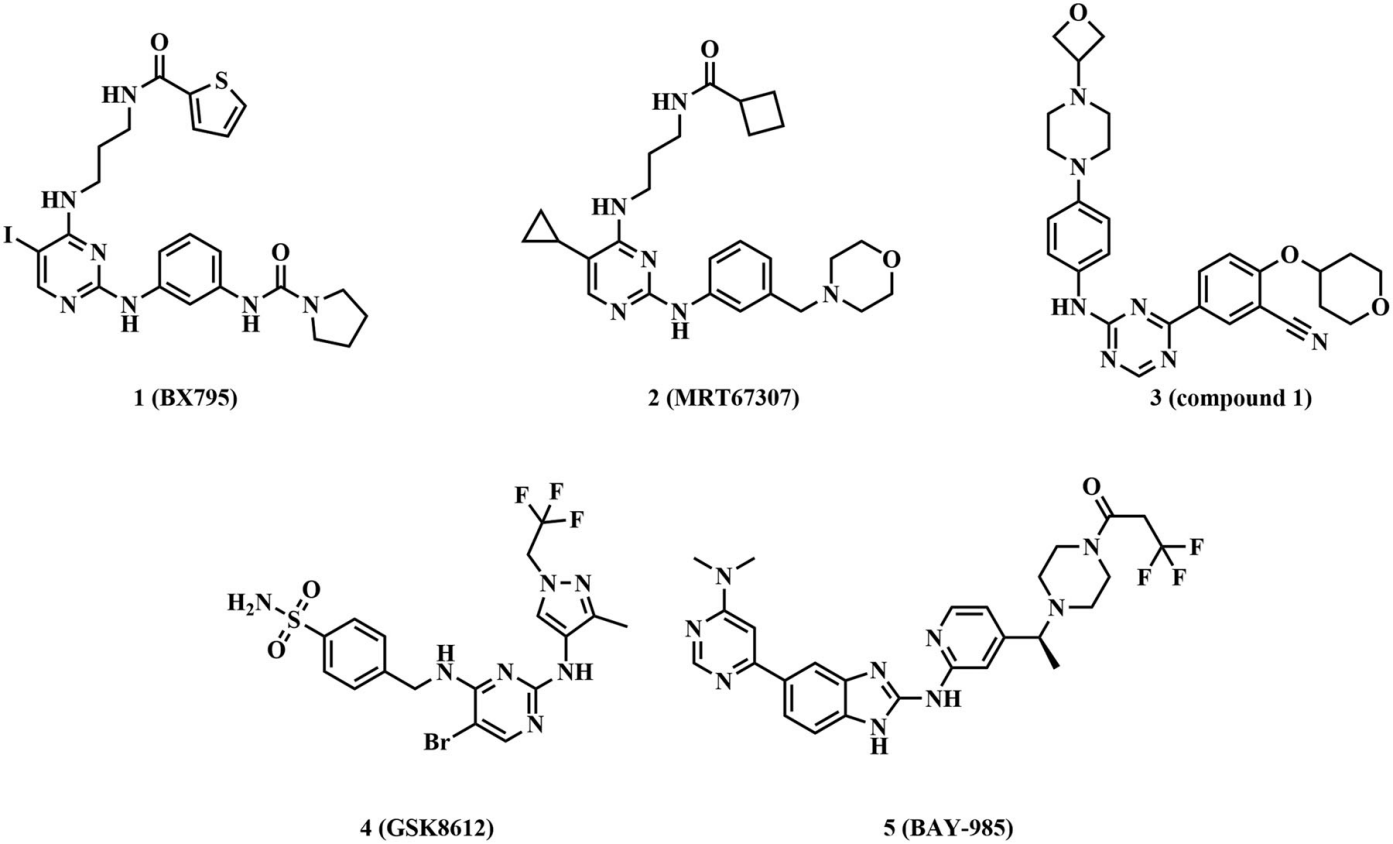
Structures of potent TBK1 inhibitors.

Currently, there are no TBK1 inhibitors in the clinical trials. A few representative small molecule inhibitors of TBK1 were depicted in Figure 1. **BX795** (**1**) was initially designed as an inhibitor of PDK1, and then researchers found that it displayed nanomolar activity on TBK1 (IC_50_ = 2 nM) and IKKε (IC_50_ = 9 nM).^20–22^ Although **BX795** is a multi-target kinase inhibitor, the researchers obtained the co-crystal structure of **BX795** and TBK1 (PDB ID 4IM2), which revealed the binding mode of inhibitors of this type and TBK1, laying the foundation for the follow-up studies. **BX795** was optimised by Clark group in the University of Dundee to generate **MRT67307** (**2**),^23^^,^^24^ which also presented robust suppressive activity on TBK1 (IC_50_ = 19 nM) and IKKε (IC_50_ = 160 nM). Furthermore, it possessed almost no inhibitory effect on IKKα and IKKβ at a concentration of 10 µM. **Compound 1** (**3**),^25^ a potent TBK1/IKKε inhibitor, showed the IC_50_ values of 1.0 nM and 5.6 nM against TBK1 and IKKε, respectively. It could enhance the response to PD-1 blockade and effectively predict the tumour response *in vivo*. In recent years, two TBK1 inhibitors **GSK8612** (**5**)^26^ and **BAY-985** (**6**),^27^ reported by GlaxoSmithKline and Bayer, respectively, served as ideal probes to further dissect the biological function of TBK1 in models of immunity and cancer. **GSK8612** exhibited an excellent kinase selectivity with pK_d_ of 8.0. **BAY-985** showed strong inhibition activity on TBK1 (IC_50_ = 2 nM) and IKKε (IC_50_ = 2 nM); however, it displayed weak antitumour activity in a xenograft model of SK-MEL-2 human melanoma cell line.

### 1.1. Rationale of the design

**Figure 2.**
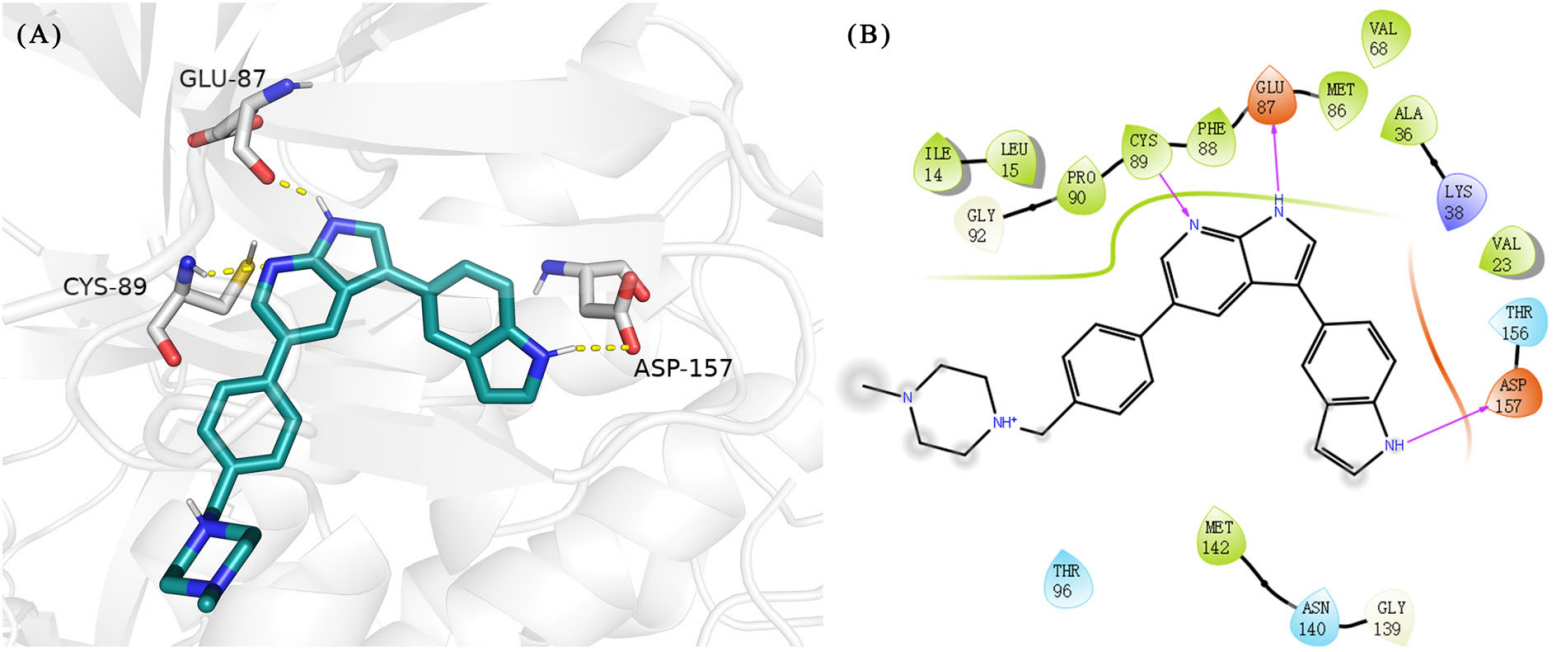
(A) The binding mode of **URMC-099** (coloured by element with carbons in teal) in the TBK1 active site. The kinase was depicted in white cartoon, and interactions were illustrated with yellow dashed lines. (B) 2D diagram of the interaction between **URMC-099** and TBK1.

**Figure 3.**
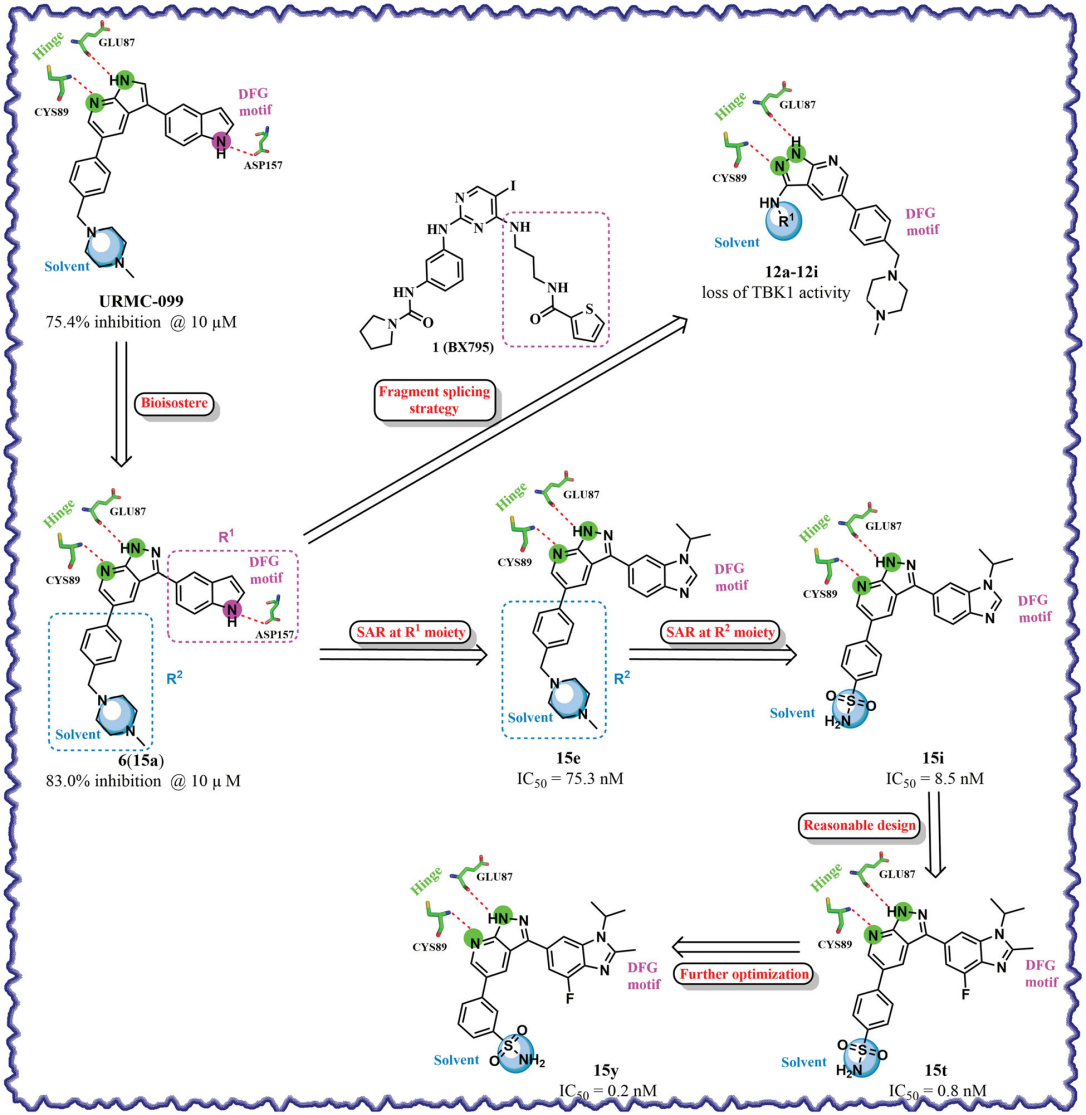
Design and modification strategies of novel TBK1 inhibitors.

Based on the in-house kinase compound library, we performed a TBK1 screening campaign and screened out the azaindole skeleton compound **URMC-099** with the inhibition rate of 75.3% on TBK1 at the concentration of 10 µM. In an effort to gain potent TBK1 inhibitors, we first docked and analysed the binding mode of **URMC-099** and TBK1 (PDB code 4IWQ) (Figure 2). The interactions showed that the NH of indole formed a hydrogen bond with Asp157 of DFG motif, the NH and N of azaindole formed two hydrogen bonds with hinge residue Glu87 and Cys89, and polar-fragment methylpiperazine extended to the solvent region. On the basis of the synthesis accessibility and the binding mode of azaindole, nitrogen atom was introduced at the 2-position of pyrrole ring according to the principle of bioisostere, which was expected to enhance the anchoring effect between NH and Glu87 of hinge region under the strong electron-withdrawing function of pyrazole N atom. Therefore, we designed and synthesised the compound **6** (**15a**, 83.0% inhibition @ 10 µM) as a hit. The docking study displayed that the binding model of **6** was same as that of **URMC-099**. Subsequently, two modification sites (R^1^ and R^2^) were chosen to guide the design and synthesis of these 1*H*-pyrazolo[3,4-b]pyridine derivatives (Figure 3).

On the basis of above analysis and with the aid of computer-aided drug design (CADD), we described here our efforts to discover a novel class of potent TBK1 inhibitors by applying structure-based drug design (SBDD). In the light of the designed pyrazolopyridine core, we fixed the methylpiperazine fragment in the solvent region and first investigated the structure of the indole ring extending to the DFG region. Hereby, two-series compounds were designed: one (compounds **12a**–**12i**) was that we employed fragment splicing strategy to introduce the similar alkylamino side chain of **BX795** and explore the chain length; the other (compounds **15a**–**15f**) was that allowing for the hydrogen bond between NH of indole ring and Asp157, we modified the indole ring and analysed the importance of the hydrogen bond. Unfortunately, the first-series compounds had little activity on TBK1. We speculated that the main reason might be that the binding mode of anchoring region was changed from pyridopyrazole to aminopyrazole as a result of the introduction of alkylamine fragment. Fortunately, the second-series compound **15e** exhibited strong inhibitory activity on TBK1 with an IC_50_ value of 75.3 nM after replacing indole ring with 1-isopropylbenzimidazole, which suggested compound **15e** became an appropriate lead in hit to lead stage. Meanwhile, we concluded that Asp157 was an important amino acid for TBK1-dependent activity. Subsequently, we carried out the next structural modification around sites R^1^ and R^2^. With the **15i** (IC_50_ = 8.5 nM) and 1**5t** (IC_50_ = 0.8 nM) were obtained, further optimisation and structure–activity relationships (SARs) study were conducted, which led to identification of a potent TBK1 inhibitor **15y** (IC_50_ = 0.2 nM). The design, synthesis, biological evaluation, and docking study of these inhibitors are discussed in this manuscript.

## 2. Materials and methods

### 2.1. Chemistry

All reagents used were commercially available without further purification. Solvents were purified according to standard procedures. Flash chromatography was performed on silica gel (300–400 mesh ASTM) and monitored by thin layer chromatography (TLC) on HSGF-254 (10–40 µm) TLC plates. Nuclear magnetic resonance (NMR) data were collected on a Varian Mercury-300 High Performance Digital FT-NMR, a Varian Mercury-400 High Performance Digital FT-NMR, a Bruker Ultrashield 500 NMR, or an Agilent 1260 Prospekt 2 Bruker Ascend 600 NMR. HRMS were carried out on a Thermo Finnigan MAT-95 spectrometer (for EI), or on a Waters, Q-Tof Ultima Global spectrometer (for ESI). The purity of compounds was determined by Gilson-215 high performance liquid chromatography (HPLC) using an YMC ODS3 column (50 mm × 4.6 mm, 5 µm) and confirmed to be more than 95%, monitored by UV absorption at 214 and 254 nm. Conditions were as follows: CH_3_CN/H_2_O eluent at 2.5 ml/min flow containing 0.1% trifluoroacetic acid (TFA) at 35 °C, 8 min, gradient 5% CH_3_CN to 95% CH_3_CN.

### 2.2. Biological evaluation

#### 2.2.1. Enzymatic assay

The TBK1 kinase activity of the novel compounds were evaluated by the FRET-based Z'-LYTE assay (Invitrogen, PV3178) following the manufacture’s instruction. Briefly, test compounds were added to the mixture of 4 ng of TBK1 kinase (Thermo Scientific, A31513) into each well of a 384 well-plate (Corning, 3514) and to react with 4 µM substrate peptide in 100 µM ATP for 1 h at room temperature. Subsequently 5 µL of development reagent was added into each well for further 1 h until 5 µL of stop reagents were added to eliminate the reaction. Fluorescence signals were measured by SpectraMax Paradigm (Molecular Devices).

#### 2.2.2. Kinase selectivity profile

Compound **15y** was evaluated for their inhibitory activities against 31 kinases at a single concentration (1 µM). Two different assay platforms were available for profiling—activity and binding. The Z′-LYTE and Adapta kinase activity assays were used most extensively for profiling and a smaller subset of kinases might be profiled using the LanthaScreen Eu Kinase Binding Assays.

#### 2.2.3. mRNA detection of TBK1 downstream genes

Human monocyte THP-1 cells and murine macrophage RAW264.7 cells were purchased form ATCC and were cultured in RMPI or DMEM medium supplement with 10% FBS. Both cells were seeded overnight and were pre-treated with different compounds for 2 h, and then were stimulated with either 0.1 µg/mL poly(I:C) or 1 µg/mL LPS for 3 h, respectively. Total RNA was extracted from cultured cells using EZ-press RNA purification kit (EZBioscience, B0004DP). Afterwards, extracted RNA was reverse transcribed into first strand cDNA by HiScript III RT Super Mix for qPCR (Vazyme, R323-00) and was applied for quantitative real-time polymerase chain reaction (RT-PCR) via ChamQ Universal SYBR qPCR Master Mix (Vazyme, Q711-02) with the BIO-RAD CFX96 C1000 touch thermal cycler. The amplification conditions were followed by protocol of SYBR qPCR Master Mix (Vazyme, Q711-02). All RT-PCR experiments were tested in triplicate, and the relative expression of genes were normalised to the control gene β-actin using the 2-ΔΔCq method. Data are shown in mean ± SD value, and t-test was performed to compare the significance between control group and treated groups by GraphPad 8.0.

The primers used for RT-PCR were as follows: *ifnb* (human)-Forward: GGCACAACAGGTAGTAGGCG; *ifnb* (human)-Reverse: GTGGAGAAGCACAACAGGAGA; *cxcl-10* (human)-Forward: CCTGCAAGCCAATTTTGTCCA; *cxcl-10* (human)-Reverse: TGTGGTCCATCCTTGGAAGC; *β-actin* (human)-Forward: GAGCACAGAGCCTCGCCTTT; *β-actin* (human)-Reverse: TCATCATCCATGGTGAGCTGGC; *ifnb* (mouse)-Forward: CAACAGCTACGCCTGGATGG; *ifnb* (mouse)-Reverse: CCTGCAACCACCACTCATTC; *cxcl-10* (mouse)-Forward: AGTGCTGCCGTCATTTTCTG; *cxcl-10* (mouse)-Reverse: TCCCTATGGCCCTCATTCTCA; *β-actin* (mouse)-Forward: GTCGAGTCGCGTCCACC; *β-actin* (mouse)-Reverse: ACGATGGAGGGGAATACAGC.

#### 2.2.4. Antiproliferative activity of compound 15y

A172, U87MG, A375, A2058, and Panc0504 cell lines were obtained from ATCC and cultured in indicated medium according to ATCC’s instructions. Cells were seeded in 96 well-plates at a destiny of around 2000 cells per well one day prior to administrated to increasing doses of indicated compounds. After 72 h of treatment, cells were washed with PBS and were fixed by 10% trichloroacetic acid before stained by sulphorhodamine B (SRB) solution. Unstained SRB were washed away by 1% acetic acid to reduce background signals. A Tris-based solution (10 mM) were used to dissolve stained SRB and the absorbance at 540 nm was measured with SpectraMax Paradigm (Molecular Devices). Inhibition rate was calculated by the relative absorbance value of test compound wells with the average of control wells plus 100%.

#### 2.2.5. Molecular docking

The TBK1 crystal structure (PDB code: 4IWQ), which was downloaded from the protein data bank (https://www.rcsb.org/), was processed with the Protein Preparation Wizard in the Schrçdinger suite. The protein structure was adjusted and modified, followed by adding hydrogen atoms, deleting solvent water molecules, and defining right bonds orders using Prime. The protonation and tautomeric states of Asp, Lys, and His were assigned at pH 7.4 state. Afterward, all hydrogen atoms of TBK1 complexes were optimised with OPLS_2005 force field, which minimised and converged heavy atoms to an RMSD of 0.3. The four selected inhibitors were prepared by using LigPrep from the Schrçdinger suite with the OPLS_2005 force field. The structure of inhibitors was also adjusted and modified, followed by adding all hydrogen atoms, checking the bond order and atom types. The prepared protein ligand complex was imported into Glide 9.7, which defined it as the receptor structure with size box (15 Å × 15 Å × 15 Å). Based on the OPLS_2005 force field, the grid of TBK1 crystal structure was generated. The standard precision (SP) mode was set for docking studies without constrained binding to gain results.

## 3. Results and discussion

### 3.1. Chemistry

**Scheme 1.**
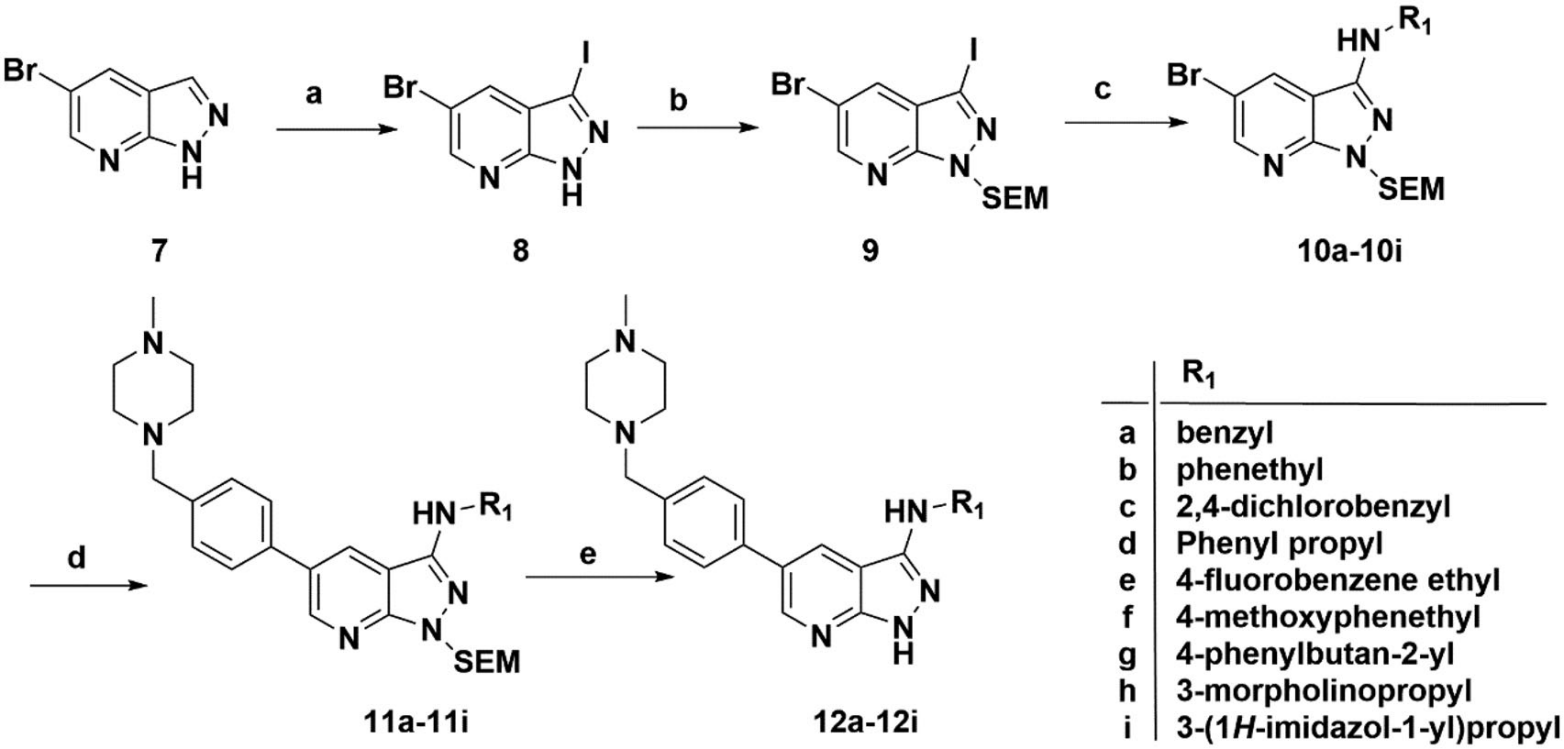
Synthesis of target compounds **12a**–**12i**. Reagents and conditions: (a) NIS, DMF, 80 °C, 8 h; (b) NaH, SEM-Cl, DMF, 0 °C–r.t., 10 h; (c) Ar(CH_2_CH_2_)_n_NH_2_, Pd_2_(dba)_3_, Xtanphos, *t*-BuONa, 1,4-dioxane, 80 °C, 10 h; (d) 1-Methyl-4-[4–(4,4,5,5-tetramethyl-1,3,2-dioxaborolan-2-yl)benzyl]piperazine, Pd(PPh_3_)_4_, Na_2_CO_3_, 1,4-dioxane: H_2_O = 4: 1, 80 °C, 6 h; and (e) 4 M HCl in 1,4-dioxane, r.t., 4 h.

Target compounds **12a**–**12i** and **15a**–**15aa** were synthesised in a few steps from the key intermediate **9**, an pyrazolo[3,4-b]pyridine core protected with SEM. The key intermediate **9** was prepared by a previously reported synthetic route, which was optimised moderately.^28^^,^^29^ Then, palladium-catalysed C-N coupling reaction was utilised to obtain corresponding intermediates **10a**–**10i**. Subsequently, a palladium-catalysed Suzuki reaction between **10a**–**10i** and 1-methyl-4-[4–(4,4,5,5-tetramethyl-1,3,2-dioxaborolan-2-yl)benzyl]piperazine were conducted to get intermediates **11a**–**11i**, and then the SEM protecting group was removed to yield the desired products **12a**–**12i** (Scheme 1).

**Scheme 2.**
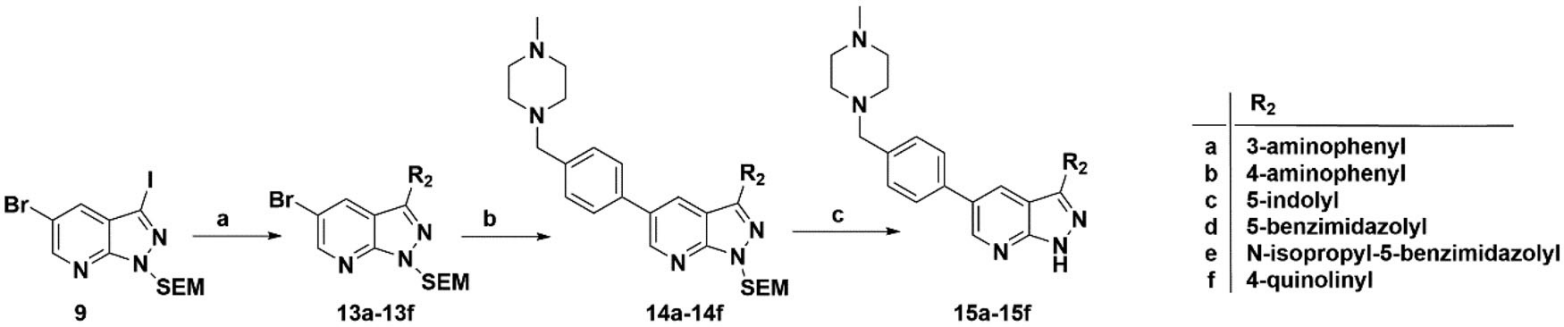
Synthesis of target compounds **15a**–**15f**. Reagents and conditions: (a) Arylboric acid, Pd(PPh_3_)_4_, Na_2_CO_3_, 1,4-dioxane: H_2_O = 4: 1, 80 °C, 6 h; (b) 1-Methyl-4-[4–(4,4,5,5-tetramethyl-1,3,2-dioxaborolan-2-yl)benzyl]piperazine, Pd(PPh_3_)_4_, Na_2_CO_3_, 1,4-dioxane: H_2_O = 4: 1, 80 °C, 6 h; and (c) 4 M HCl in 1,4-dioxane, r.t., 4 h.

Similarly, after two-step palladium-catalysed Suzuki reactions of intermediate **9** and the removal of the SEM group, the desired products **15a**–**15f** were obtained (Scheme 2).

**Scheme 3.**
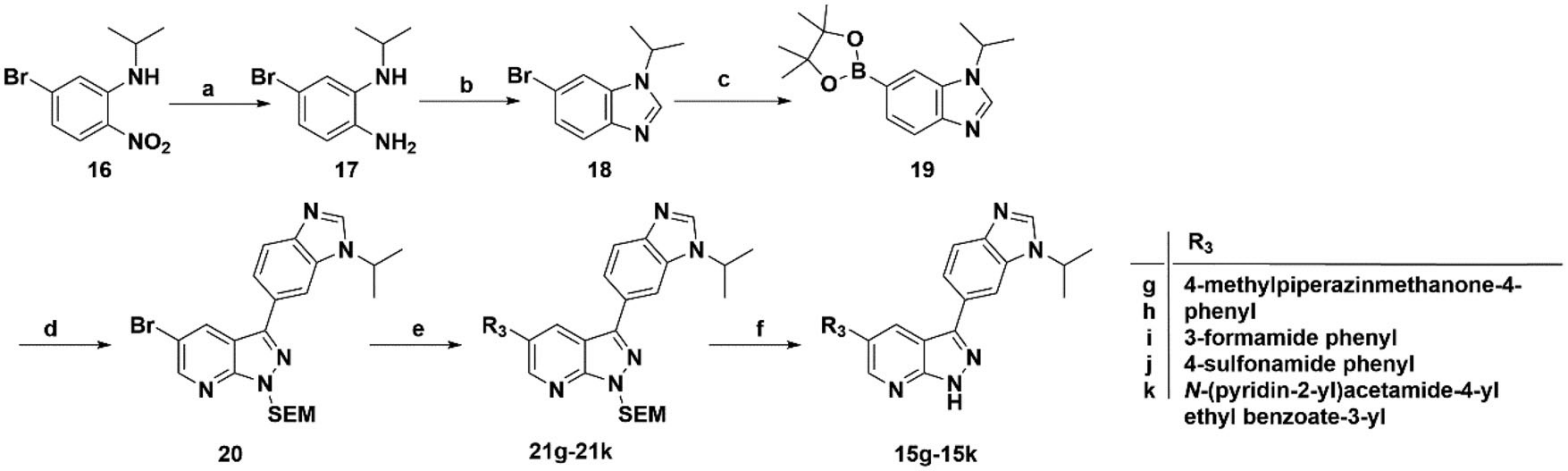
Synthesis of target compounds **15g**–**15k**. Reagents and conditions: (a) Fe, NH_4_Cl aq., 80 °C, 2 h; (b) HCOOH, reflux, 6 h; (c) Bis(pinacolato)diboron, Pd(dppf)Cl_2_, KOAc, 1,4-dioxane, 100 °C, 10 h; (d) Intermediate **9**, Pd(PPh_3_)_4_, Na_2_CO_3_, 1,4-dioxane: H_2_O = 4: 1, 80 °C, 6 h; (e) Arylboric acid, Pd(PPh_3_)_4_, Na_2_CO_3_, 1,4-dioxane: H_2_O = 4: 1, 80 °C, 6 h; and (f) 4 M HCl in 1,4-dioxane, r.t., 4 h.

For the synthesis of target compounds **15g**–**15k** (Scheme 3), 5-bromo-N-isopropyl-2-nitroaniline (**16**) was used as the starting material. Compound **16** was reduced to intermediate **17** by iron powder, followed by the retaining ring reaction with formic acid obtained intermediate **18**, which were converted to intermediate **19** through bis(pinacolato)diboron reaction. Treatment of intermediate **19** with two-step palladium-catalysed Suzuki reactions, followed removing the SEM protecting group to afford end-product **15g**–**15k.**

**Scheme 4.**
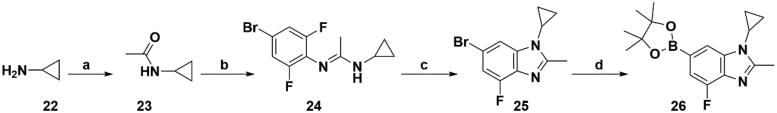
Synthesis of the intermediate **26**. Reagents and conditions: (a) i. TEA, Acetic anhydride, DCM, r.t., 8 h; ii. Et_2_O, K_2_CO_3_ r.t., 10 h; (b) 4-Bromo-2,6-difluoroaniline, POCl_3_, TEA, Toluene, reflux, 8 h; (c) *t*-BuOK, THF, 80 °C, 6 h; and (d) Bis(pinacolato)diboron, Pd(dppf)Cl_2_, KOAc, 1,4-dioxane, 100 °C, 10 h.

**Scheme 5.**
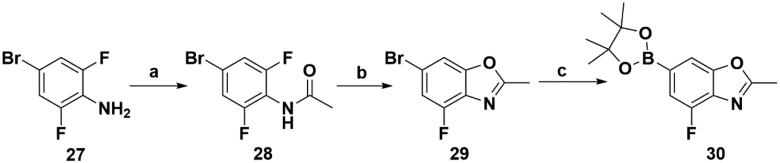
Synthesis of the intermediate **30**. Reagents and conditions: (a) Ac_2_O, AcOH, r.t., 5 h; (b) Cs_2_CO_3_, NMP, 150 °C, 10 h; and (c) Bis(pinacolato)diboron, Pd(dppf)Cl_2_, KOAc, 1,4-dioxane, 100 °C, 10 h.

**Scheme 6.**
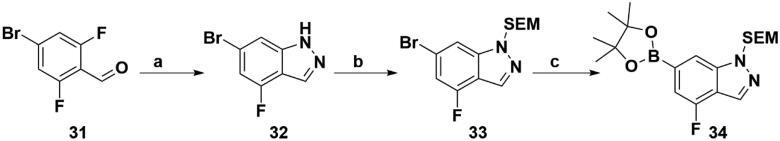
Synthesis of the intermediate **34**. Reagents and conditions: (a) N_2_H_4_·H_2_O, 1,4-dioxane, 90 °C, 5 h; (b) NaH, SEM-Cl, DMF, 0 °C–r.t., 10 h; and (c) Bis(pinacolato)diboron, Pd(dppf)Cl_2_, KOAc, 1,4-dioxane, 100 °C, 10 h.

**Scheme 7.**
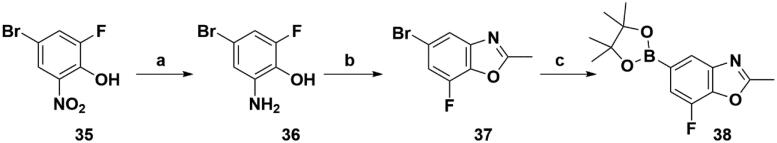
Synthesis of the intermediate **38**. Reagents and conditions: (a) Fe, NH_4_Cl aq., 80 °C, 2 h; (b) TMOA, 150 °C, 6 h; and (c) Bis(pinacolato)diboron, Pd(dppf)Cl_2_, KOAc, 1,4-dioxane, 100 °C, 10 h.

**Scheme 8.**
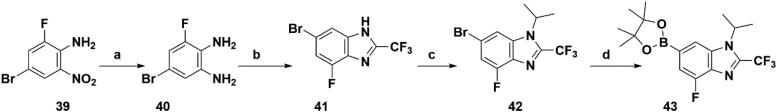
Synthesis of the intermediate **43**. Reagents and conditions: (a) Fe, NH_4_Cl aq., 80 °C, 2 h; (b) CF_3_COOH, 70 °C, 4 h; (c) NaH, SEM-Cl, DMF, 0 °C–r.t., 10 h; and (d) Bis(pinacolato)diboron, Pd(dppf)Cl_2_, KOAc, 1,4-dioxane, 100 °C, 10 h.

Intermediates **26, 30, 34, 38,** and **43** were prepared according to the procedures described in Schemes 4–8. Cyclopropylamine (**22**) was condensed with acetic anhydride to gain intermediate **23**, which was further substituted to get intermediate **24**. Compound **25** was synthesised by the cyclisation of intermediate **24** under basic conditions. Finally, the key intermediate **26** was prepared by Miyaura borylation reaction from the intermediate **25**. The intermediate **30** was afforded from 4-bromo-2,6-difluoroaniline (**27**) via acetylation, cyclisation, and Miyaura borylation reaction. Treatment of 4-bromo-2,6-difluorobenzaldehyde (**31**) with hydrazine hydrate afforded intermediate **32**, then **32** was protected with SEM group to provide intermediate **33**, which further reacted with bis(pinacolato)diboron to yield the key intermediate **34**. The key intermediate **38** was prepared by reduction, cyclisation, and Miyaura borylation reaction with the starting material 4-bromo-2-fluoro-6-nitrophenol (**35**). 4-Bromo-2-fluoro-6-nitroaniline (**39**) yielded the key intermediate **43** via reduction, cyclisation, isopropyl substitution, and Miyaura borylation reaction.

**Scheme 9.**
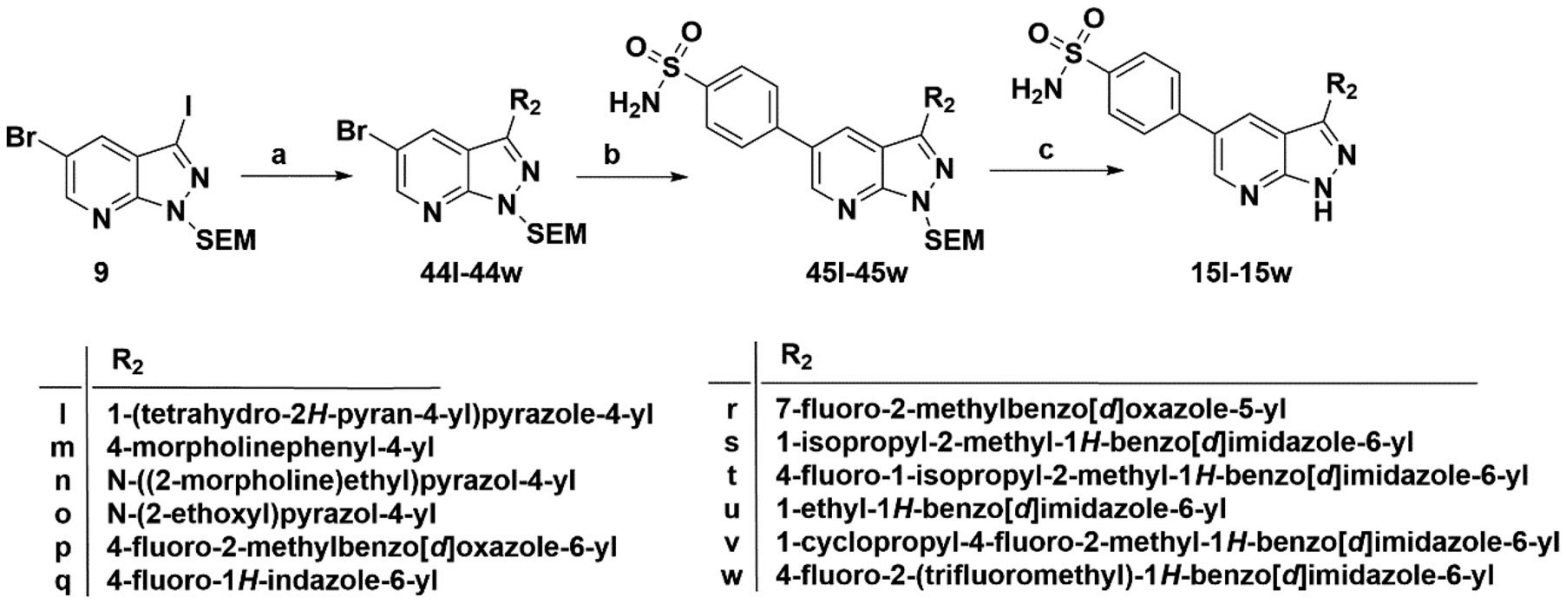
Synthesis of target compounds **15l**–**15w**. Reagents and conditions: (a) Arylboric acid, Pd(PPh_3_)_4_, Na_2_CO_3_, 1,4-dioxane: H_2_O = 4: 1, 80 °C, 6 h; (b) 4-(Aminosulfonyl) phenylboronic acid, Pd(PPh_3_)_4_, Na_2_CO_3_, 1,4-dioxane: H_2_O = 4: 1, 80 °C, 6 h; and (c) 4 M HCl in 1,4-dioxane, r.t., 4 h.

**Scheme 10.**
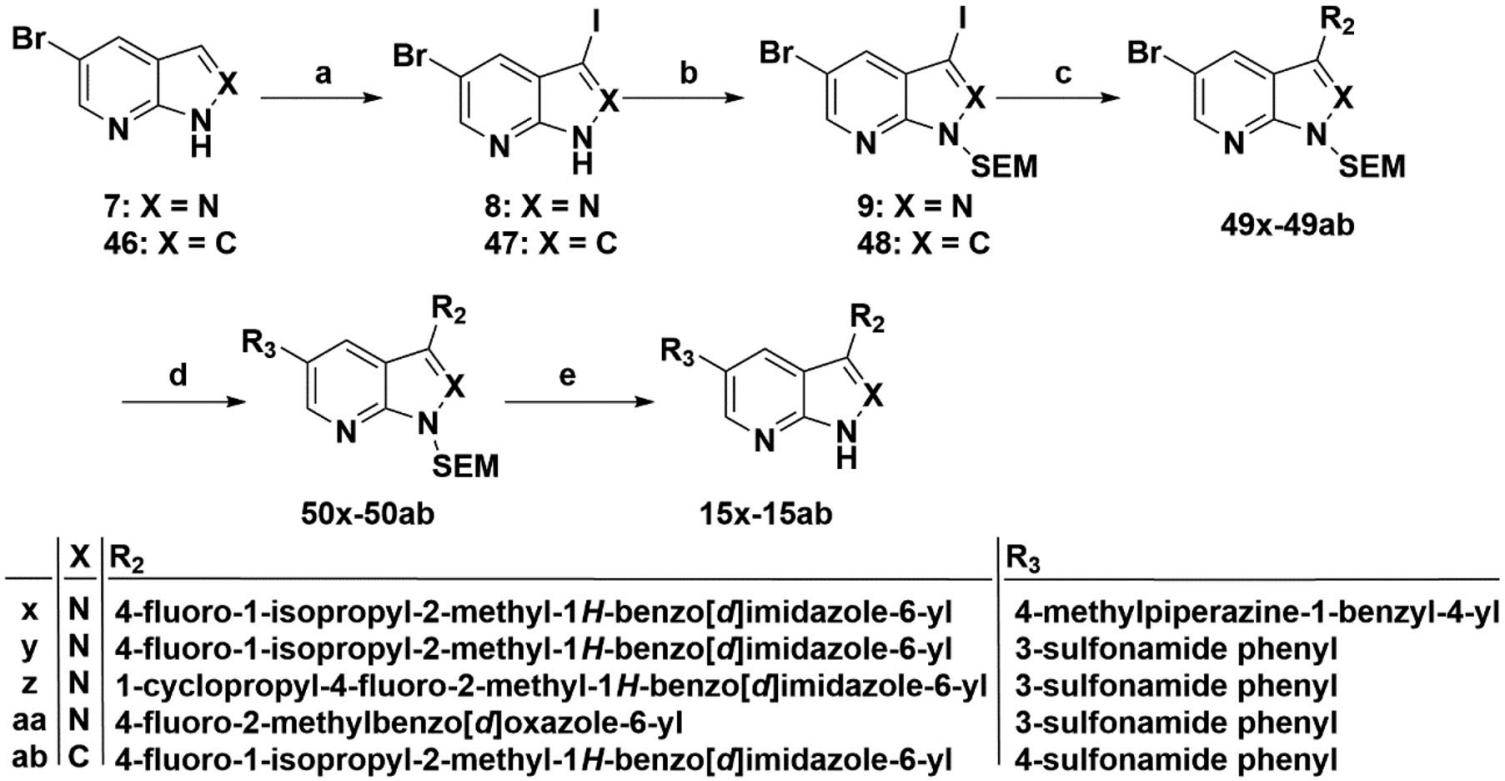
Synthesis of target compounds **15x**–**15ab**. Reagents and conditions: (a) NIS, DMF, 80 °C, 8 h; (b) NaH, SEM-Cl, DMF, 0 °C–r.t., 10 h; (c) Arylboric acid, Pd(PPh_3_)_4_, Na_2_CO_3_, 1,4-dioxane: H_2_O = 4: 1, 80 °C, 6 h; (d) Arylboric acid, Pd(PPh_3_)_4_, Na_2_CO_3_, 1,4-dioxane: H_2_O = 4: 1, 80 °C, 6 h; and (e) 4 M HCl in 1,4-dioxane, r.t., 4 h.

As depicted in Schemes 9 and 10, target compounds **15l**–**15ab** were prepared by using procedures similar to those described in Schemes 1 and 2, and for compound **15ab**, azaindole core (**46**) was used instead of pyrazolo[3,4-b]pyridine core (**7**).

### 3.2. Biological evaluation

#### 3.2.1. Target compounds design and in vitro activity against TBK1 kinase

The *in vitro* TBK1 inhibition activity of all the pyrazolo[3,4-b]pyridine derivatives was compared to the positive compounds **BX795** and **MRT67307**. The IC_50_ value of all compounds could not be tested until the inhibition rate reached 50% at the concentration of 1 µM. Under our experimental conditions, **BX795** and **MRT67307** exhibited TBK1 inhibition activity with IC_50_ values of 7.1 nM and 28.7 nM, respectively, which was similar to previously reported data.

**Figure 4.**
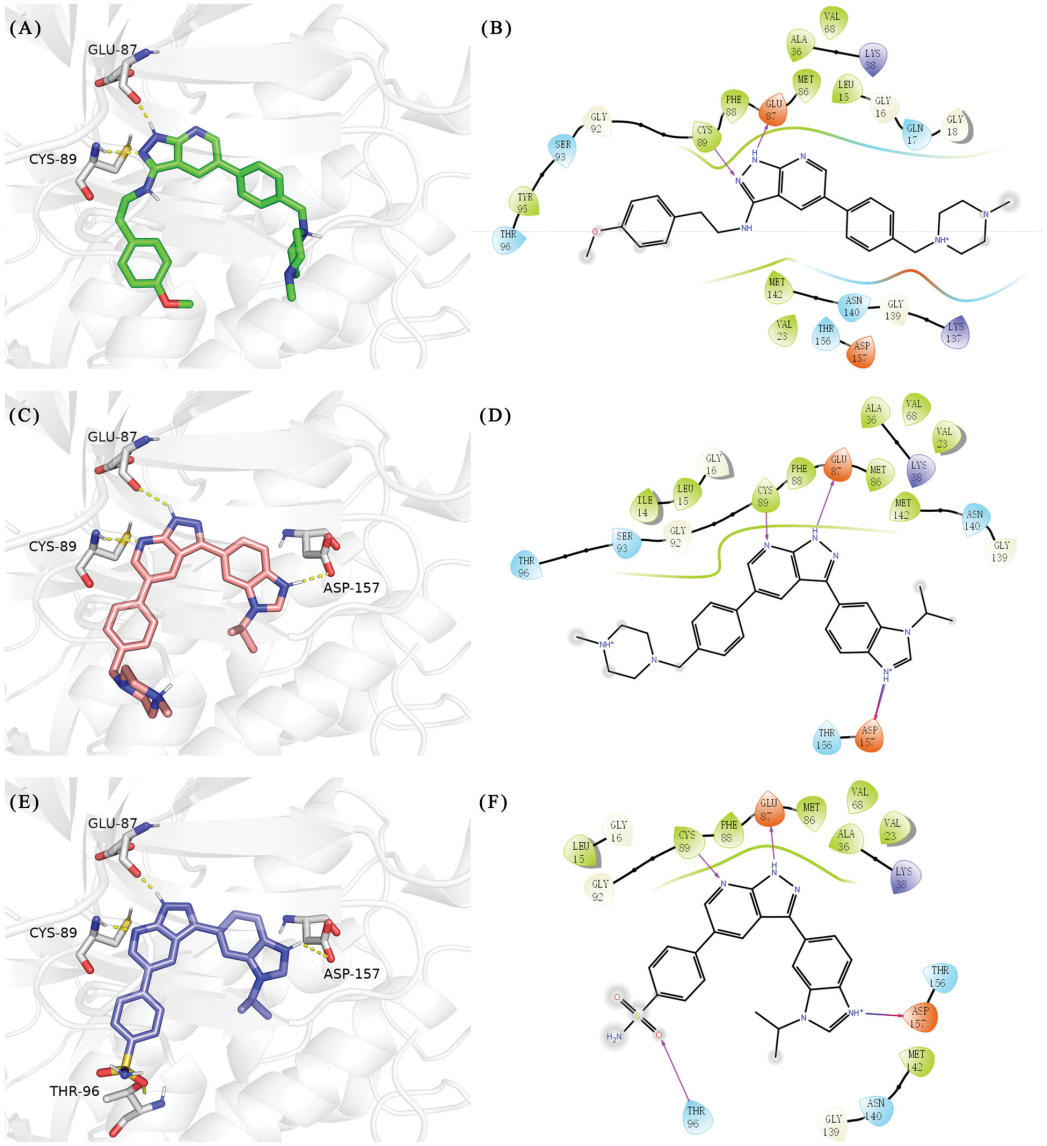
(A) The binding mode of **12f** (coloured by element with carbons in green) in the TBK1 active site. (B) 2D diagram of the interaction between compound **12f** and TBK1. (C) The binding mode of **15e** (coloured by element with carbons in salmon pink) in the TBK1 active site. (D) 2 D diagram of the interaction between compound **15e** and TBK1. (E) The binding mode of **15i** (coloured by element with carbons in slate) in the TBK1 active site. (F) 2D diagram of the interaction between compound **15i** and TBK1. The kinase was depicted in white cartoon, and interactions were illustrated with yellow dashed lines.

According to the binding mode of the above designed hit compound **6**（**15a**, 83.0% inhibition @ 10 µM) and TBK1, we first synthesised first-series nine pyrazolo[3,4-b]pyridine compounds **12a**–**12i** by fragment splicing strategy to introduce the alkylamino side chain of **BX795**, which were characterised by replacing indole ring with 1–3 carbon-length alkylamino fragments at R_2_ position. Unfortunately, as shown in Table 1, all the compounds designed above exhibited no significant inhibitory activity on TBK1 regardless of the electron-withdrawing or electron-donating substituents on the aromatic ring. We speculated the reason for the loss of their activities as follows: take compound **12f** as an example, the docking study indicated that N and NH of pyrazole moiety in **12f** replacing pyridine N and pyrazole NH of pyrazolopyridine moiety formed two hydrogen bonds with Glu87 and Cys89 in hinge region of the TBK1, which completely reversed the binding model between molecule and protein, thus making methylpiperazine extend to DFG region away from solvent region (Figure 4(A,B)).

**Table 1.** *In vitro* TBK1 kinase inhibitory activity of the **12a**–**12i**, **15a**–**15f**.^a^

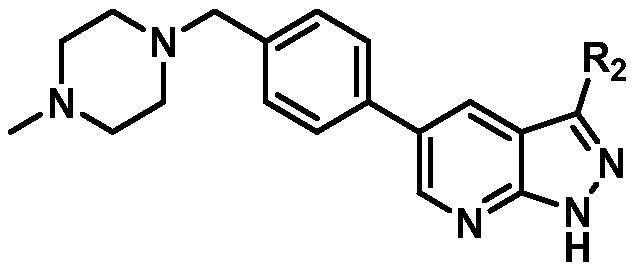						
Compound	R_2_	TBK1 inhibition (%)				IC_50_/nM
		10 μM	1 μM	100 nM	10 nM	
**12a**	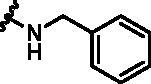	−8.0	−13.6	−14.5	N.T.^b^	N.T.
**12b**	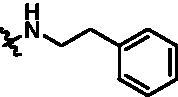	3.2	5.9	7.3	N.T.	N.T.
**12c**	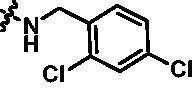	−2.0	−6.7	−30.1	N.T.	N.T.
**12d**	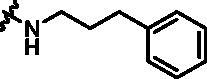	−4.7	−5.8	−5.6	N.T.	N.T.
**12e**	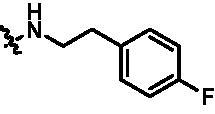	−5.0	−2.4	−1.4	N.T.	N.T.
**12f**	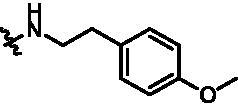	−0.5	−7.9	−9.7	N.T.	N.T.
**12g**	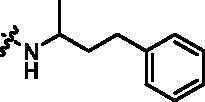	−4.9	−5.2	−6.2	N.T.	N.T.
**12h**	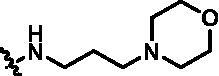	−5.2	−4.3	−7.8	N.T.	N.T.
**12i**	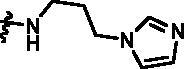	7.1	19.9	15.2	16.2	N.T.
**15a**	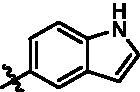	83.0	36.5	13.2	N.T.	N.T.
**15b**	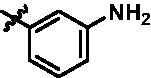	47.9	14.3	11.0	N.T.	N.T.
**15c**	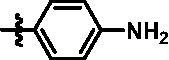	86.8	47.8	38.3	N.T.	N.T.
**15d**	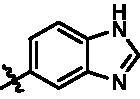	102.2	84.1	52.5	18.0	130.6
**15e**	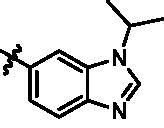	107.6	103.9	62.8	14.3	75.3
**15f**	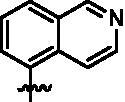	101.4	81.4	28.7	N.T.	290.4
**BX795**		N.T.	N.T.	99.6	72.9	7.1
**MRT67307**		N.T.	N.T.	92.8	29.6	28.7

^a^The IC_50_ values are shown as the mean (nM) values from two separate experiments.

^b^N.T. = not tested.

In addition, in order to explore the importance of hydrogen bond between Asp157 and indole of compound **6**, we also synthesised and tested the inhibitory activity of the second-series five pyrazolo[3,4-b]pyridine compounds **15b**–**15f**, and the result was shown in Table 1. Considering that Asp157 was an acidic amino acid, we expected to increase the basicity of N atom and form a salt bridge with Asp157 to further enhance the receptor-ligand interaction. The indole ring of compound **15a** was opened to obtain aniline (**15c**) which slightly improved the activity. However, the activity of compound **15b** decreased significantly, which might be explained by meta-aniline far away from Asp157. When indole was substituted by benzimidazole or isoquinoline with stronger basicity, the activities of **15d** and **15f** were obviously increased. And a potent lead compound **15e** (IC_50_ = 75.3 nM) was confirmed by introducing isopropyl to the N atom of benzimidazole. The docking study showed that imidazole of **15e** and Asp157 formed a salt bridge (Figure 4(C,D)). We concluded that Asp157 was an important amino acid for TBK1-dependent activity and it was also significant to introduce hydrophobic fragments into the hydrophobic cavity adjacent to the DFG motif.

After further studying the distribution of amino acids in the active cavity, we found that there were some amino acids such as Thr96, Ser93, and Leu15 in the solvent region that could form hydrogen bonds with ligands. Therefore, different hydrophilic fragments were introduced in R_3_ moiety of **15e** to obtain compounds **15g**–**15k** (Table 2). Except compound **15k** (IC_50_ = 287.7 nM), the IC_50_ value of other compounds was less than 100 nM, among which compound **15i** (IC_50_ = 8.5 nM) displayed potent inhibition activity and the IC_50_ value was nearly 10 times lower than compound **15e**. The docking study of compound **15i** was showed Figure 4(E,F), and the oxygen atom of sulphonamide could form hydrogen bond with Ser96, which might be the main reason of the further improvement of activity.

**Table 2.** *In vitro* TBK1 kinase inhibitory activity of the **15g**–**15k**.^a^

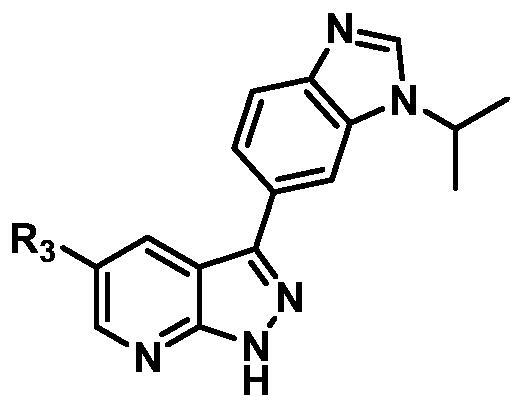							
Compound	R_3_	TBK1 inhibition（%）					IC_50_/nM
		10 μM	1 μM	100 nM	10 nM	1 nM	
**15g**	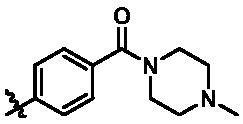	107.8	108.5	91.7	29.9	8.6	26.0
**15h**	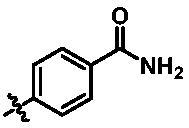	103.4	103.5	75.6	29.1	24.0	58.9
**15i**	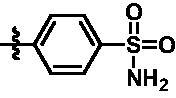	110.7	104.6	75.4	62.3	47.3	8.5
**15j**	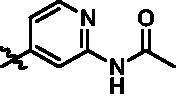	102.9	104.5	67.1	31.5	14.2	67.2
**15k**	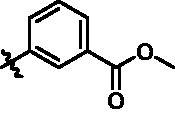	106.3	82.6	45.0	19.5	N.T.^b^	287.7
**BX795**		N.T.	N.T.	99.6	72.9	37.7	7.1
**MRT67307**		N.T.	N.T.	92.8	29.6	2.4	28.7

^a^The IC_50_ values are shown as the mean (nM) values from two separate experiments.

^b^N.T. = not tested.

**Figure 5.**
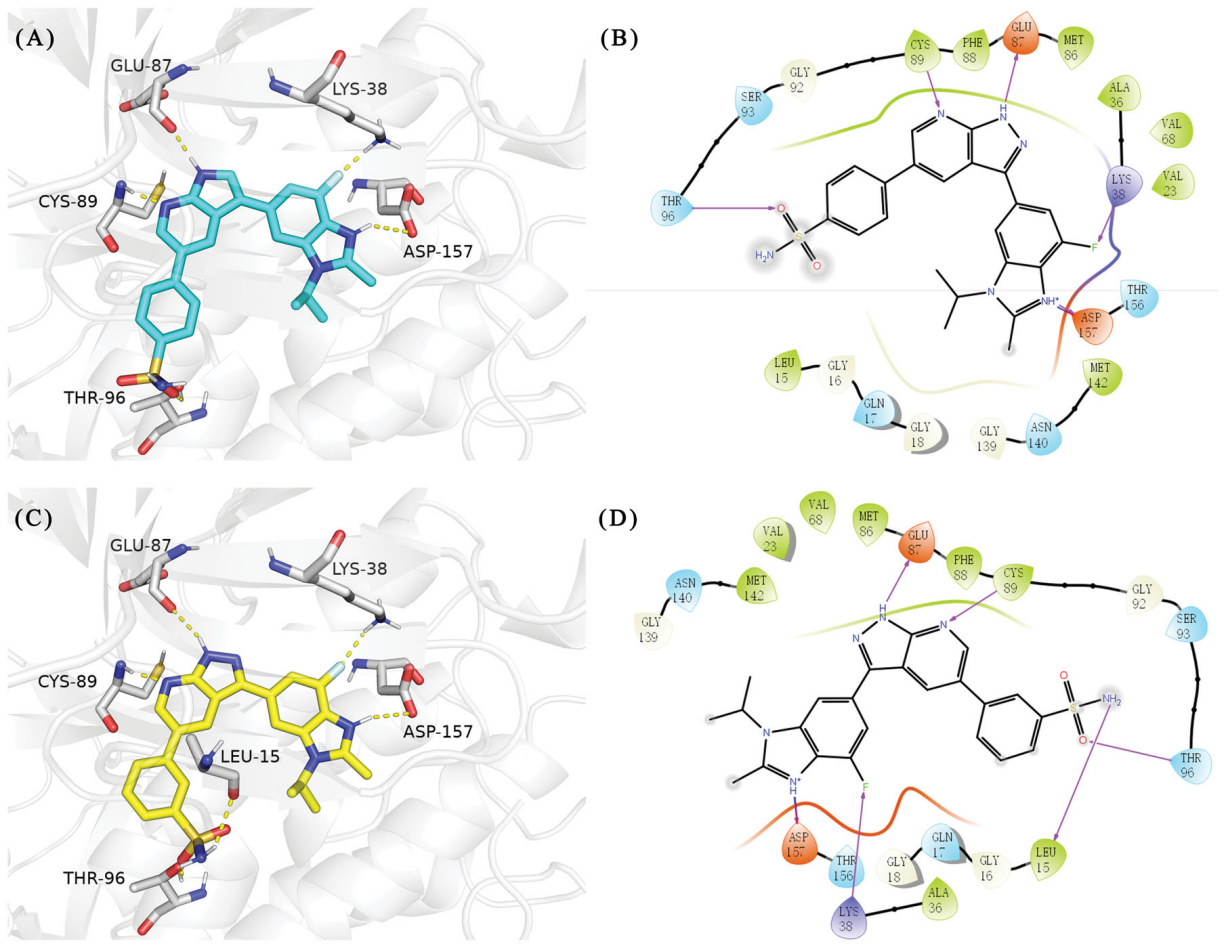
(A) The binding mode of **15t** (coloured by element with carbons in cyans) in the TBK1 active site. (B) 2D diagram of the interaction between compound **15t** and TBK1. (C) The binding mode of **15y** (coloured by element with carbons in yellow) in the TBK1 active site. (D) 2D diagram of the interaction between compound **15y** and TBK1. The kinase was depicted in white cartoon, and interactions were illustrated with yellow dashed lines.

For further exploring the SARs, we fixed the benzene sulphonamide fragment of R_3_ and investigated the influence of R_2_ on the activity according to the following three points: (1) Substitution of benzopyrazole ring by monocyclic or other bicyclic rings; (2) introducing substituents into benzimidazole ring; and (3) replacing isopropyl with other alkyl fragments. The *in vitro* kinase assays with compounds **15l**–**15w** were illustrated in Table 3. Compounds **15l**–**15r** including different substituted rings that did not form hydrogen bond with Asp157 decreased obviously, which further confirmed that Asp157 was an important amino acid in maintaining TBK1 activity. Compound **15s** replaced hydrogen atom with methyl at imidazole 2 position, and its activity remained unchanged. The activity of compound **15t** was further enhanced (IC_50_ = 0.8 nM) by introducing fluorine atom into benzene ring and was increased by 10 times compared with compound **15i**. The potency improvement of compound **15t** might be explained by the fluorine atom could form a hydrogen bond with Lys38 (Figure 5(A,B)). Compared to compounds **15i** and **15t**, introduction of ethyl and cyclopropyl (**15u** and **15v**) at the nitrogen atom of pyrazole moiety resulted in a slight decline in activity, indicating that isopropyl was the more suitable to occupy the hydrophobic cavity beside DFG motif. Compound **15w** with trifluoromethyl replacing methyl almost lost TBK1 activity. Based on the above results, we have gained a potent TBK1 inhibitor **15t** superior to positive compounds.

**Table 3.** *In vitro* TBK1 kinase inhibitory activity of the **15l**–**15w**.^a^

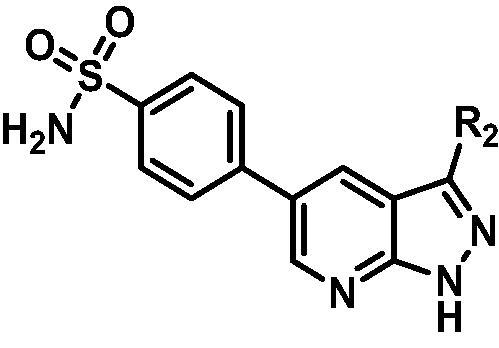								
Compound	R_2_	TBK1 inhibition（%）						IC_50_/nM
		10 μM	1 μM	100 nM	10 nM	1 nM	0.1 nM	
**15l**	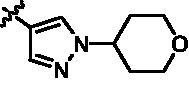	21.9	−0.6	−7.2	N.T.^b^	N.T.	N.T.	N.T.
**15m**	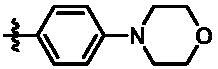	−11.0	−6.0	−8.9	N.T.	N.T.	N.T.	N.T.
**15n**	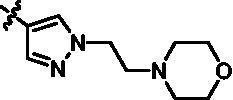	−12.5	−15.0	−8.9	N.T.	N.T.	N.T.	N.T.
**15o**	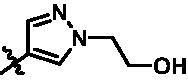	23.3	−7.4	−2.7	N.T.	N.T.	N.T.	N.T.
**15p**	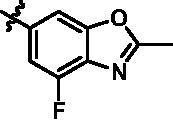	41.1	37.1	30.6	32.0	N.T.	N.T.	N.T.
**15q**	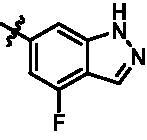	83.9	25.5	12.7	4.5	N.T.	N.T.	N.T.
**15r**	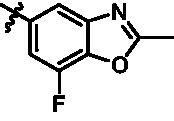	56.8	5.7	10.8	−2.6	N.T.	N.T.	N.T.
**15s**	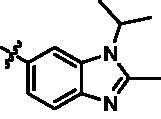	N.T.	97.2	76.9	68.6	45.5	N.T.	6.2
**15t**	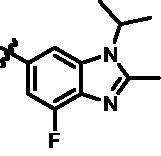	N.T.	99.1	79.5	78.0	61.6	43.1	0.8
**15u**	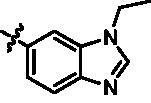	N.T.	89.6	78.1	30.0	25.1	12.1	33.3
**15v**	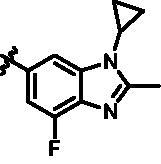	N.T.	87.4	70.3	49.7	42.5	37.7	31.2
**15w**	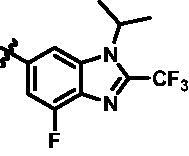	N.T.	N.T.	27.1	10.7	N.T.	N.T.	N.T.
**BX795**		N.T.	N.T.	99.6	72.9	37.7	0.18	7.1
**MRT67307**		N.T.	N.T.	92.8	29.6	2.4	<0	28.7

^a^The IC_50_ values are shown as the mean (nM) values from two separate experiments.

^b^N.T. = not tested.

In the last round of structural modification, guided by molecular docking, we discovered that the ligand could form hydrogen bonds with Thr96 and Leu15 simultaneously in solvent region after para-sulphonamide was transferred to meta-position. Compared with compound **15t,** the activity of compound **15y** (IC_50_ = 0.2 nM) further increased by four times (Table 4), and the docking result was shown in Figure 5(C,D). Protonated N of benzimidazole ring formed a salt bridge with Asp157 of DFG motif, and fluorine atom formed a hydrogen bond with Lys38; Two hydrogen bonds were formed between NH and N of pyrazolopyridine and hinge region residues Glu87 and Cys89, respectively; NH and O of benzene sulphonamide formed two hydrogen bonds with solvent region residues Leu15 and Thr96, respectively, helping to explain the high affinity imparted by this moiety. Furthermore, we also verified that benzene sulphonamide fragment (**15y**) in solvent region was superior to methylpiperazine (**15x**), isopropyl on nitrogen atom was superior to cyclopropyl (**15z**), and trifluoromethyl substitution (**15aa**) was unfavourable for activity. Consistent with the previous analysis, when the N atom was replaced by C atom, compound **15ab** showed 8-fold reduced activity against TBK1 compared to **15t**. Finally, the most potent TBK1 inhibitor **15y** was selected for further biological evaluation.

**Table 4.** *In vitro* TBK1 kinase inhibitory activity of the **15x**–**15ab**.^a^ 
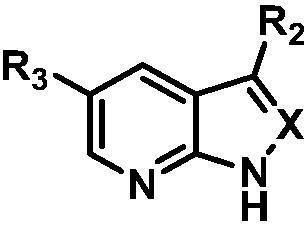

Compd.	X	R_2_	R_3_	TBK1 inhibition（%）					IC_50_/nM
				1 μM	100 nM	10 nM	1 nM	0.1 nM	
**15x**	N	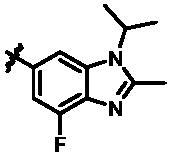	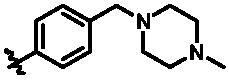	75.8	80.5	34.1	18.6	N.T.	22.0
**15y**	N	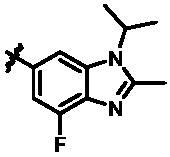	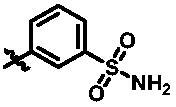	53.9	102.4	93.8	88.8	33.6	0.2
**15z**	N	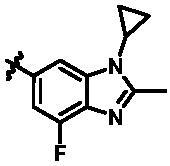	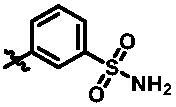	79.5	84.4	48.4	32.7	N.T.	24.4
**15aa**	N	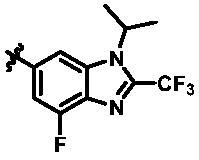	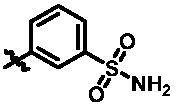	48.3	36.8	14.10	N.T.^b^	N.T.	N.T.
**15ab**	C	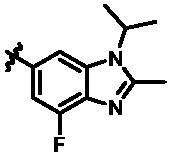	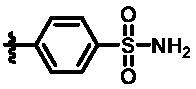	90.3	72.9	70.9	60.1	43.1	3.6
**BX795**				N.T.	99.6	72.9	37.7	0.18	7.1
**MRT67307**				N.T.	92.8	29.6	2.4	<0	28.7

^a^The IC_50_ values are shown as the mean (nM) values from two separate experiments.

^b^N.T. = not tested.

#### 3.2.2. Structure–activity relationships

**Figure 6.**
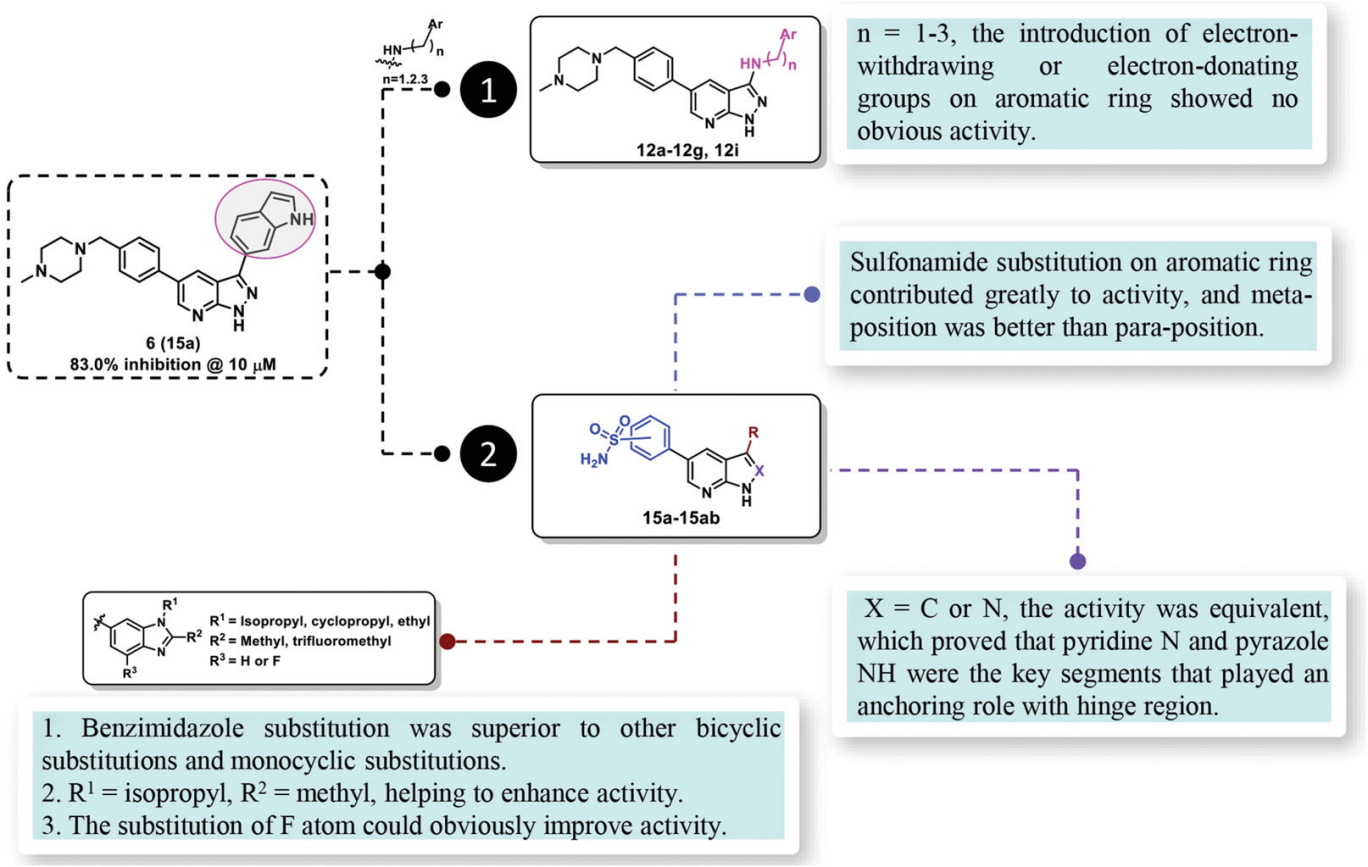
The structure–activity relationships of the designed novel compounds.

The SARs study of the novel compounds was represented in Figure 6. Starting from hit compound **6**, two-series compounds were obtained based on rational design. In the first series, when indole ring was replaced by aryl aliphatic amines with 1–3 carbon length, no matter whether the aromatic ring was an electron-withdrawing or electron-donating substituent, these compounds did not show obvious TBK1 inhibitory activity. In the second series, benzene sulphonamide substitution on pyridine ring contributed greatly to the activity, and meta-sulphonamide was better than para-sulphonamide. The benzimidazole substitution in the R part of pyrazole ring was superior to other bicyclic and monocyclic substitutions. And when R^3^ = isopropyl, R^4^ = methyl, TBK1 could be strongly inhibited. When R^5^ was a fluorine atom, the inhibitory activity of compounds on TBK1 could be significantly enhanced. When the nucleus skeleton was 1*H*-pyrazolo[3,4-b]pyridine or 1*H*-pyrrolo[2,3-b]pyridine, the inhibitory activity against TBK1 was equivalent, which proved that pyridine N and pyrazole NH, rather than pyrazole N and NH, played an anchoring role in the hinge region.

#### 3.2.3. Kinase selectivity profile

**Figure 7.**
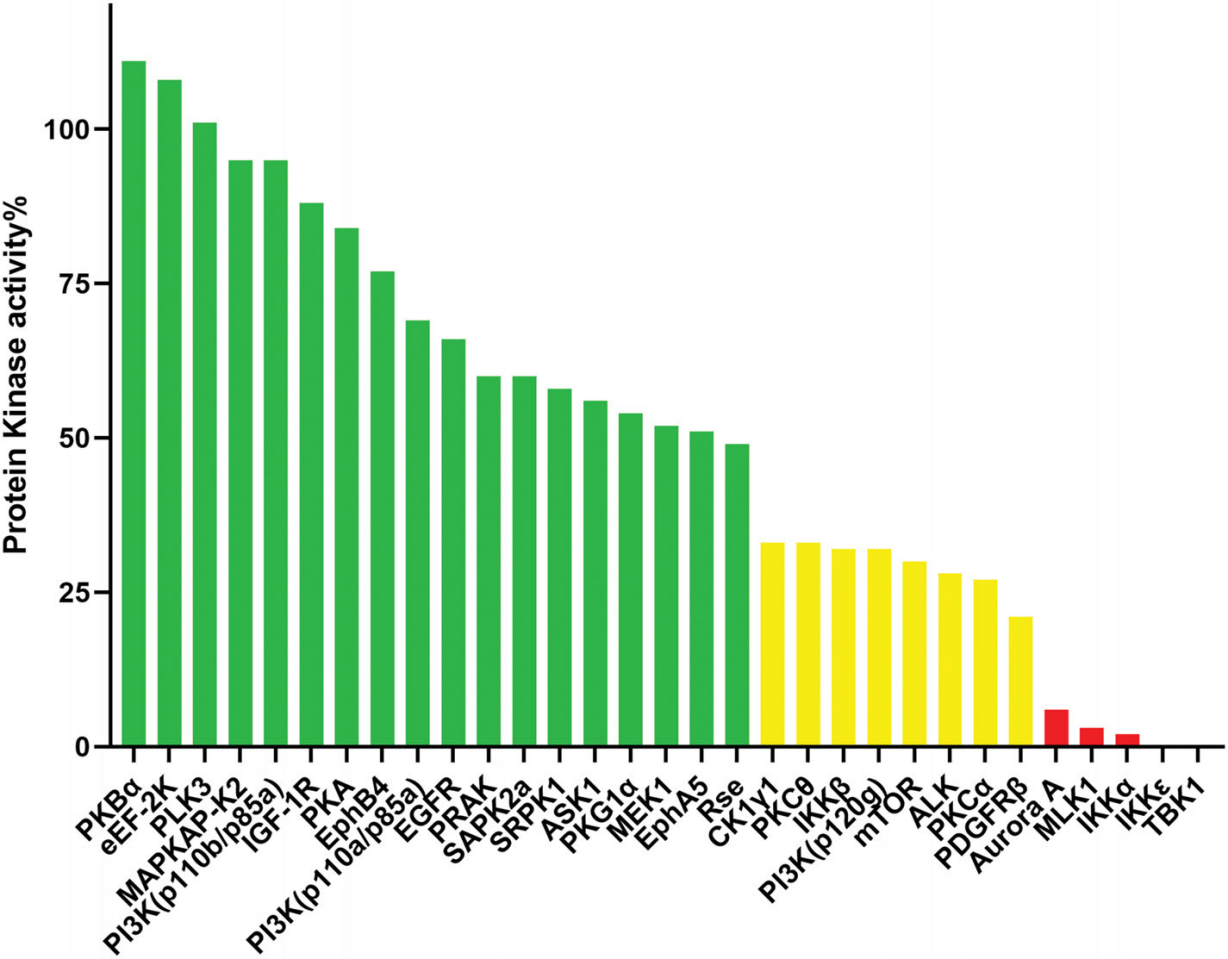
Selectivity profile of compound **15y** measured at a concentration of 1.0 μM in a pannel of 31 kinases (red columns denote <20%, yellow columns denote between 20% and 40% and green columns denote >40%).

The potent compound **15y** was subjected to kinase selectivity profiling against a panel of 31 kinases at a concentration of 1.0 µM to further evaluate the selectivity of this series, and the enzyme activity results were given in Figure 7 and Table 5. We could see that TBK1, IKKε, IKKα, MLK1, and Aurora A were produced less than 20% kinase activity (red columns in Figure 6) by compound **15y**, the activity of eight kinases (CK1γ1, PKCθ, IKKβ, PI3K(p120g), mTOR, ALK, PKCα, and PDGFRβ) ranged from 20% to 40% (yellow columns in Figure 7), and the other 18 kinases activity was greater than 40% (green columns in Figure 7).

**Table 5.** Kinase selectivity profile of compound **15y**.^a^

Kinases	%kinase activity @ 1.0 μM	Kinases	%kinase activity @ 1.0 μM	Kinases	%kinase activity @ 1.0 μM
PKBα	111	SAPK2a	60	mTOR	30
eEF-2K	108	SRPK1	58	ALK	28
PLK3	101	ASK1	56	PKCα	27
MAPKAP-K2	95	PKG1α	54	PDGFRβ	21
PI3K(p110b/p85a)	95	MEK1	52	Aurora A	6
IGF-1R	88	EphA5	51	MLK1	3
PKA	84	Rse	49	IKKα	2
EphB4	77	CK1γ1	33	IKKε	−1
PI3K(p110a/p85a)	69	PKCθ	33	TBK1	−2
EGFR	66	IKKβ	32		
PRAK	60	PI3K(p120g)	32		

Profiling Service from Life Technologies. The results represent the mean of three independent experiments performed in triplicate.

^a^Selectivity profile of compound **15y** was measured at a concentration of 1.0 μM in a panel of 31 kinases generated with the SelectScreen®.

#### 3.2.4. mRNA detection of TBK1 downstream genes

**Figure 8.**
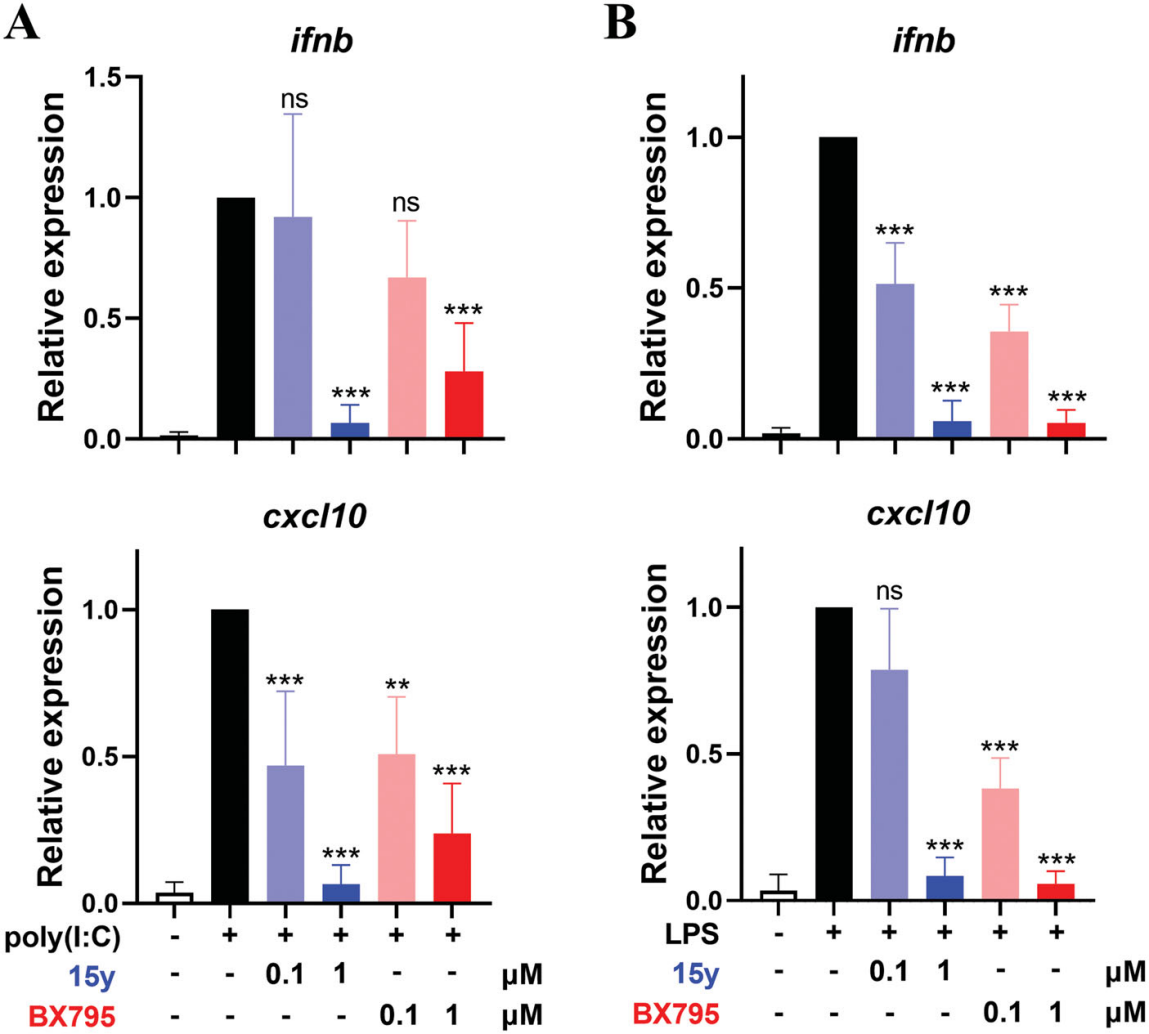
Compound **15y** inhibited the expression of *ifnb* and *cxcl10* genes expression in THP-1 cells (A) and RAW264.7 cells (B), stimulated by poly(I:C) or LPS, respectively. Data are representative of at least 3 independent experiments and are shown in mean ± SD value. The significance of the differences between poly(I:C) or LPS stimulation-only group and the stimulation plus compound treated groups were determined by One-Way ANOVA test. **p < .01; ***p < .001; ns, not significant.

Based on the potent kinase inhibitory activity and the acceptable selectivity of compound **15y**, we subsequently analysed its cellular TBK1 inhibitory activity. As it is well recognised that innate immune stimuli poly(I:C) and LPS are enable of activating TBK1-IRF3 pathway and therefore triggering a boosted expression of several IFN gene expression, such as *ifnb* and *cxcl10*^3^, we examined the activities of compound **15y** on the expression of *ifnb* and *cxcl10* stimulated by poly(I:C) or LPS in THP-1 and RAW264.7 monocytes. As shown in Figure 8, *ifnb* and *cxcl10* gene expression were both activated in these cells. As expected, compound **15y** treatment inhibited this robustly increased *ifnb* and *cxcl10* expression in THP-1 cells in a dose-dependent manner (Figure 8(A)), and achieved an almost complete inhibition at the concentration of 1 µM (both >93% inhibition), while **BX795** exhibited a much weaker effect at the same concentration. Similar results were also observed in LPS-stimulated murine RAW264.7 cells with a profound activity of compound **15y** (Figure 8(B)). These results confirmed that compound **15y** effectively inhibited TBK1 downstream IFN signalling in cells.

#### 3.2.5. Antiproliferative activity of compound 15y

**Figure 9.**
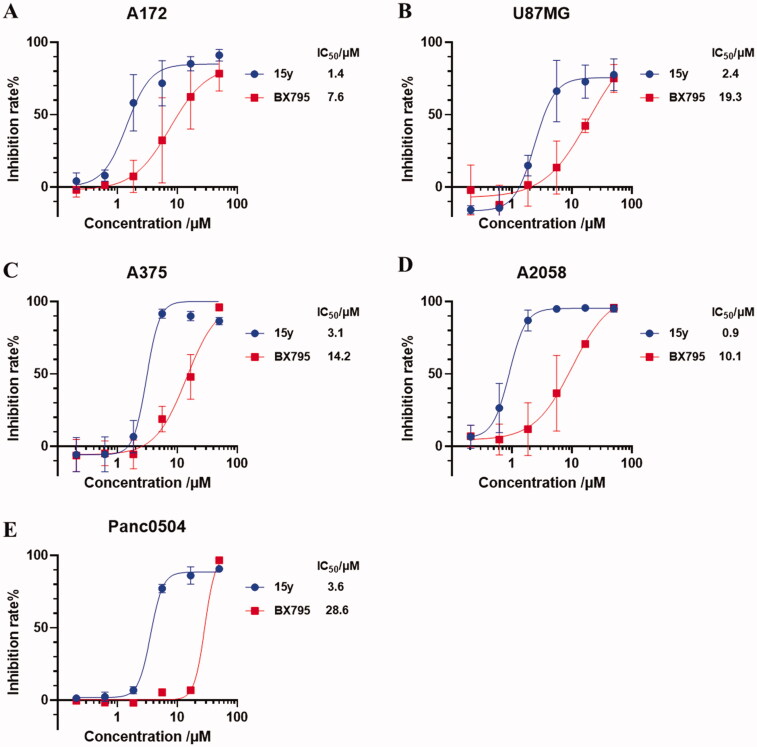
Compound **15y** inhibited the viability of two glioma cell lines A172 and U87MG (A, B), two melanoma cell lines A375 and A2058 (C, D), and one pancreatic cell line Panc0504 (E). Cell viability were measured by SRB staining method 72 h after cells treated by increasing doses of compound **15y** and **BX795**. Data are representative of three independent experiments and are shown in mean ± SD value. Nolin fit four-parameters model were used to fit the curve and to calculate IC_50_ values by GraphPad 8.0.

TBK1 is central to multiple biological processes in cancer progression, and pharmacological targeting TBK1 has been reported to induced a context-selective impairment of tumourigenesis in glioma,^15^ melanoma,^16^ pancreatic cancer,^17–18^ and other cancers. We therefore explored the anti-tumour proliferation effect of compound **15y** in different types of cancer cells, including two glioma cell lines (A172 and U87MG), two melanoma cell lines (A375 and A2058), and one pancreatic cell line (Panc0504). As demonstrated in Figure 9(A,B), compound **15y** exhibited a profound antiproliferative effect in both glioma cell lines with average IC_50_ values of 1.4 µM in A172 cells and 2.4 µM in U87MG cells. It also effectively inhibited the proliferation of A375, A2058 and Panc0504 cell lines, with IC_50_ values of 3.1 µM, 0.9 µM, and 3.6 µM, respectively (Figure 9(C–E)). In contrast, **BX795** only modestly impaired the viability of these cancer cells with IC_50_ values ranging between 7.6 µM and 28.6 µM, showing much weaker effects than compound **15y** (Figure 9(A–E)). It is worth mentioned that the antiproliferative activity of compound **15y** may not fully resulted from TBK1 inhibition due to its modest selectivity (see Figure 7 and Table 5); however, these results did suggest compound **15y** could serve as a potent anti-tumour agent. Moreover, human umbilical vein endothelial cells (HUVECs) were used to evaluate the cytotoxicity of compound **15y** to normal cells (Figure S1). The result indicated that compound **15y** showed weak toxicity to HUVECs with an IC_50_ value of 21.2 µM.

## 4. Conclusion

By analysing the binding modes of compound **URMC-099** and TBK1, we utilised the bioisostere strategy to obtained a series of 1*H*-pyrazolo[3,4-b]pyridine derivatives based on computer-aided drug design (CADD). The *in vitro* enzyme activity assays suggested the optimised compound **15y** exhibited picomolar inhibitory activity against TBK1 with an IC_50_ value of 0.2 nM. And the kinase selectivity profiling of compound **15y** presented good kinase selectivity. Subsequently, we proved that compound **15y** sufficiently inhibited the mRNA expression of TBK1-downstream genes in stimulated THP-1 and RAW264.7 cells. Also, compound **15y** exhibited a profound antiproliferation effect on A172, U87MG, A375, A2058, and Panc0504 cell lines with the IC_50_ of micromole level, which was significantly effective than **BX795**. These results indicate that compound **15y** is a novel, highly potent TBK1 inhibitor with predominant bioactivity and is predicted to be a promising tool compound that is helpful to understand functions of targeting TBK1 in immune response and cancer therapy.

## Supplementary Material

Supplemental Material
