# Supplementary material for: Identification of 1H-pyrazolo[3,4-b]pyridine derivatives as novel and potent TBK1 inhibitors: design, synthesis, biological evaluation, and molecular docking study
Source: J Enzyme Inhib Med Chem. 2022 May 19;37(1):1411–25. doi: 10.1080/14756366.2022.2076674 (PMC9132415; doi:10.1080/14756366.2022.2076674)
Supplement: Supplemental Material [file IENZ_A_2076674_SM5168.pdf]

<u>**Supporting Information**</u>

Identification of 1*H*-pyrazolo[3,4-b]pyridine derivatives as novel and potent TBK1 inhibitors: Design, synthesis, biological evaluation, and molecular docking study

Yin Sun[a,b]†, Haotian Tang[b,c]†, Xiaoyan Wang[a,b]†, Fang Feng[b], Tiantian Fan[b,c], Dongmei Zhao[a]*, Bing Xiong[b,c]*, Hua Xie[b,c,d]*, and Tongchao Liu[b]*

[a] Key Laboratory of Structure-Based Drug Design and Discovery of Ministry of Education, Shenyang Pharmaceutical University, 103 Wenhua Lu, Shenyang 110016, P. R. China [b] Shanghai Institute of Materia Medica, Chinese Academy of Sciences, 555 Zuchongzhi Road, Shanghai 201203; P. R. China [c] University of Chinese Academy of Sciences, No.19A Yuquan Road, Beijing 100049, P. R. China. [d] Zhongshan Institute for Drug Discovery, Shanghai Institute of Materia Medica, Chinese Academy of Sciences, 555 Zuchongzhi Road, Shanghai 201203

Corresponding authors: Dongmei Zhao *medchemzhao@163.com* Shenyang Pharmaceutical University, 103 Wenhua Lu, Shenyang 110016, P. R. China; Bing Xiong *bxiong@simm.ac.cn* Shanghai Institute of Materia Medica, Chinese Academy of Sciences, 555 Zuchongzhi Road, Shanghai 201203; P. R. China; Hua Xie *hxie@simm.ac.cn* Shanghai Institute of Materia Medica, Chinese Academy of Sciences, 555 Zuchongzhi Road, Shanghai 201203; P. R. China; Tongchao Liu *tongchao_liu@simm.ac.cn* Shanghai Institute of Materia Medica, Chinese Academy of Sciences, 555 Zuchongzhi Road, Shanghai 201203; P. R. China

†These three authors contributed equally to this work.

Contents

1. **The cytotoxicity of 15y on HUVECs**………..…….……………………..….S2
2. **Chemistry**………...…………………………………………………….S3 – S19
3. **Copies of analytical data for selected compounds**………………….S20 – S94

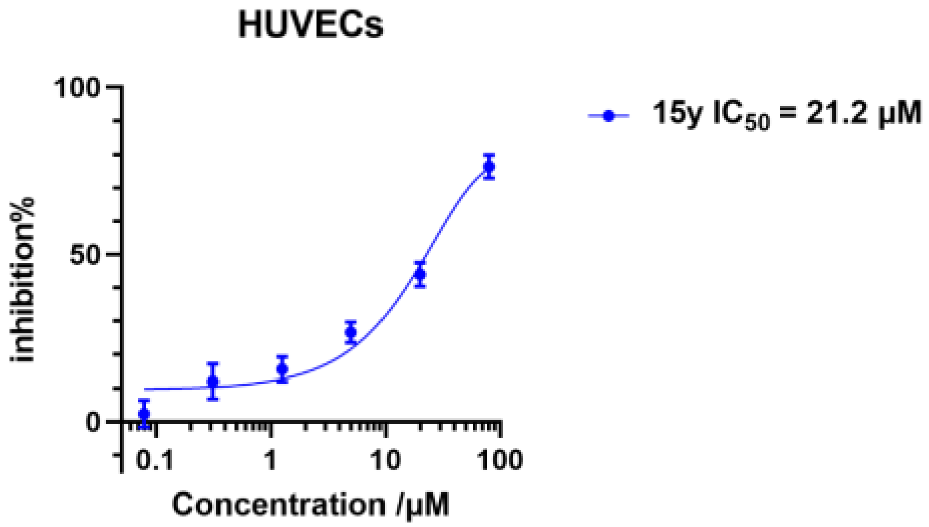

Figure S1. The cytotoxicity of compound **15y** on Human Umbilical Vein Endothelial Cells (HUVECs). Data are representative of 3 independent experiments and are shown in mean ± SD value. Nolin fit 4-parameters model were used to fit the curve and to calculate IC_{50} values by GraphPad 8.0.

*General procedure for the synthesis of intermediates (**10a** - **10i**)*

To a solution of intermediate **9** (1 equiv) and corresponding amines (1 equiv) in anhydrous 1.4-dioxane was added t-BuONa (2 equiv), $Pd_2(dba)_3$ (0.1 equiv) and Xtanphos (0.1 equiv). The mixture was bubbled with Ar for 5 min. The reaction solution was heated at 80 °C for 10 h before being concentrated and purified by column chromatography on silica gel (dichloromethane/methanol (v/v) = 200 : l) to afford the intermediate **10a** - **10i**.

*General procedure for the synthesis of intermediates (**11a** - **11i**)*

Intermediates **10a** - **10i** (1 equiv), 1-Methyl-4-[4-(4,4,5,5-tetramethyl-1,3,2-dioxaborolan-2-yl)benzyl]piperazine (1 equiv), Na_2CO_3 (2 equiv), $Pd(PPh_3)$ (0.1 equiv) were added to 1,4-dioxane and H_2O (4 : 1). Under an argon atmosphere, the reaction solution was heated at 80 °C for 6 h before being concentrated and purified by column chromatography on silica gel (dichloromethane/methanol (v/v) = 100 : l) to afford the intermediate **11a** - **11i**.

*General procedure for the synthesis of target compounds (**12a** - **12i**)*

Intermediates **10a-10i** were dissolved in a solution of HCl/1,4-dioxane (4 N). The mixture was stirred for 4 h at room temperature. The reaction was monitored with TLC. The mixture was filtered to afford compounds **12a** - **12i** as a white solid.

*N-benzyl-5-(4-((4-methylpiperazin-1-yl)methyl)phenyl)-1H-pyrazolo[3,4-b]pyridin-3-amine(**12a**)*

White solid. Yield: 62%. ^{1}H NMR (400 MHz, CD_3OD) δ 8.66 (s, 1H), 8.42 (s, 1H), 7.62 (s, 2H), 7.46 (d, *J* = 7.1 Hz, 4H), 7.33 (d, *J* = 7.7 Hz, 2H), 7.26 (s, 1H), 4.58 (s, 2H), 3.60 (d, *J* = 5.1 Hz, 2H), 2.57 (s, 8H), 2.33 (s, 3H). ^{13}C NMR (126 MHz, DMSO-d_6) δ 152.14, 148.90, 147.69, 140.43, 137.10, 129.63, 129.40 (2C), 129.31 (2C), 128.15, 127.86 (2C), 127.43 (2C), 126.63, 126.25, 106.09, 61.37, 54.19 (2C), 51.71, 46.29 (2C), 44.82. HRMS (ESI): m/z $[M + H]^+$ calcd for $C_{25}H_{28}N_6$ 413.2454, found 413.2452. Retention time 2.294 min, HPLC purity = 95.854%.

*5-(4-((4-methylpiperazin-1-yl)methyl)phenyl)-N-phenethyl-1H-pyrazolo[3,4-b]pyridin-3-amine(**12b**)*

White solid. Yield: 59%. ^{1}H NMR (400 MHz, CD_3OD) δ 8.63 (d, *J* = 1.8 Hz, 1H), 8.34 (d, *J* = 1.8 Hz, 1H), 7.62 (d, *J* = 8.1 Hz, 2H), 7.45 (d, *J* = 8.0 Hz, 2H), 7.27 (d, *J* = 4.0 Hz, 4H), 7.18 (dd, *J* = 8.4, 4.3 Hz, 1H), 3.65 (d, *J* = 3.0 Hz, 2H), 3.60 (d, *J* = 7.8 Hz, 2H), 3.03 – 2.98 (m, 2H), 2.85 (s, 4H), 2.66 (s, 4H), 2.54 (s, 3H). ^{13}C NMR

(126 MHz, DMSO-d_6) δ 152.09, 148.96, 147.67, 140.12, 137.18, 129.68, 129.62 (2C), 128.67 (2C), 128.28, 127.11, 126.31 (2C), 126.09, 125.94 (2C), 106.13, 61.12, 53.75 (2C), 51.03, 44.40 (2C), 44.14, 35.16. HRMS (ESI): m/z $[M + H]^+$ calcd for $C_{26}H_{30}N_6$ 427.2610, found 427.2605. Retention time 2.349 min, HPLC purity = 98.261%.

*N-(2,4-dichlorobenzyl)-5-(4-((4-methylpiperazin-1-yl)methyl)phenyl)-1H-pyrazolo[3,4-b]pyridin-3-amine(**12c**)*

White solid. Yield: 54%. ^1H NMR (400 MHz, CD_3OD) δ 8.68 (d, *J* = 2.1 Hz, 1H), 8.44 (d, *J* = 2.1 Hz, 1H), 7.65 (d, *J* = 8.1 Hz, 2H), 7.52 (d, *J* = 8.3 Hz, 2H), 7.47 (d, *J* = 5.9 Hz, 2H), 7.29 (dd, *J* = 8.3, 2.1 Hz, 1H), 4.66 (s, 2H), 3.64 (s, 2H), 2.71 (m, 8H), 2.45 (s, 3H). ^{13}C NMR (126 MHz, DMSO-d_6) δ 152.11, 148.31, 147.88, 136.82, 136.57, 136.19, 133.19, 131.87, 130.35 (2C), 129.62, 128.46 (2C), 127.15, 126.89, 126.37 (2C), 105.89, 61.45, 54.36 (2C), 51.98, 45.10, 43.44 (2C). HRMS (ESI): m/z $[M + H]^+$ calcd for $C_{25}H_{26}Cl_2N_6$ 481.1674, found 481.1671. Retention time 2.379 min, HPLC purity = 98.462%.

*5-(4-((4-methylpiperazin-1-yl)methyl)phenyl)-N-(3-phenylpropyl)-1H-pyrazolo[3,4-b]pyridin-3-amine(**12d**)*

White solid. Yield: 54%. ^1H NMR (400 MHz, CD_3OD) δ 8.66 (d, *J* = 2.1 Hz, 1H), 8.40 (d, *J* = 2.1 Hz, 1H), 7.65 (d, *J* = 8.1 Hz, 2H), 7.48 (d, *J* = 8.1 Hz, 2H), 7.26 (d, *J* = 6.4 Hz, 4H), 7.17 (d, *J* = 6.2 Hz, 1H), 3.68 (s, 2H), 3.42 (d, *J* = 7.0 Hz, 2H), 2.91 (s, 4H), 2.80 (s, 2H), 2.70 (s, 4H), 2.59 (s, 3H), 2.07 – 2.03 (m, 2H). ^{13}C NMR (126 MHz, DMSO-d_6) δ 152.59, 149.70, 148.09, 142.47, 137.70, 136.81, 130.17 (2C), 128.79 (2C), 128.75, 127.70 (2C), 126.80, 126.52, 126.15 (2C), 106.65, 61.58, 54.13 (2C), 51.43, 44.55, 42.67 (2C), 33.28, 31.27. HRMS (ESI): m/z $[M + H]^+$ calcd for $C_{27}H_{32}N_6$ 441.2767, found 441.2766. Retention time 2.420 min, HPLC purity = 99.182%.

*N-(4-fluorophenethyl)-5-(4-((4-methylpiperazin-1-yl)methyl)phenyl)-1H-pyrazolo[3,4-b]pyridin-3-amine(**12e**)*

White solid. Yield: 50%. ^1H NMR (400 MHz, CD_3OD) δ 8.66 (d, *J* = 2.1 Hz, 1H), 8.37 (d, *J* = 2.1 Hz, 1H), 7.65 – 7.63 (m, 2H), 7.48 (d, *J* = 8.2 Hz, 2H), 7.32 – 7.29 (m, 2H), 7.04 – 7.00 (m, 2H), 3.66 (s, 2H), 3.62 (t, *J* = 7.4 Hz, 2H), 3.01 (t, *J* = 7.3 Hz, 2H), 2.96-2.64 (m, 8H), 2.53 (s, 3H). ^{13}C NMR (126 MHz, DMSO-d_6) δ 161.25 (d, *J* = 241.9 Hz), 152.57, 149.42, 148.17, 137.61, 136.74 (2C), 130.91 (2C), 130.14 (2C), 127.60, 126.79 (2C), 126.61, 115.48, 115.32, 106.60, 61.73, 54.43 (2C), 51.87, 44.98, 44.86 (2C), 34.68. HRMS (ESI): m/z $[M + H]^+$ calcd for $C_{26}H_{29}FN_6$ 445.2516,

found 445.2513. Retention time 2.416 min, HPLC purity = 99.656%.

*N-(4-methoxyphenethyl)-5-(4-((4-methylpiperazin-1-yl)methyl)phenyl)-1H-pyrazolo[3,4-b]pyridin-3-amine(**12f**)*

White solid. Yield: 58%. ^{1}H NMR (400 MHz, CD_3OD) δ 8.66 (d, *J* = 2.1 Hz, 1H), 8.36 (d, *J* = 2.1 Hz, 1H), 7.64 (d, *J* = 8.2 Hz, 2H), 7.47 (d, *J* = 8.2 Hz, 2H), 7.21 (d, *J* = 8.6 Hz, 2H), 6.87 – 6.84 (m, 2H), 3.77 (s, 3H), 3.67 (s, 2H), 3.58 (d, *J* = 7.7 Hz, 2H), 2.96 (s, 2H), 2.89-2.69 (m, 8H), 2.58 (s, 3H). HRMS (ESI): m/z $[M + H]^+$ calcd for $C_{27}H_{32}N_6O$ 457.2716, found 457.2716. Retention time 2.336 min, HPLC purity = 98.351%.

*5-(4-((4-methylpiperazin-1-yl)methyl)phenyl)-N-(3-phenylbutyl)-1H-pyrazolo[3,4-b]pyridin-3-amine(**12g**)*

White solid. Yield: 57%. ^{1}H NMR (500 MHz, DMSO-d_6) δ 12.00 (s, 1H), 8.68 (d, *J* = 2.2 Hz, 1H), 8.50 (d, *J* = 2.2 Hz, 1H), 7.66 (d, *J* = 8.2 Hz, 2H), 7.42 (d, *J* = 8.1 Hz, 2H), 7.26 (d, *J* = 7.2 Hz, 2H), 7.23 – 7.21 (m, 2H), 7.17 (d, *J* = 7.2 Hz, 1H), 6.12 (d, *J* = 7.8 Hz, 1H), 3.80 (s, 1H), 3.56 (s, 2H), 2.71 (s, 8H), 2.40 (s, 3H), 1.27 (s, 3H), 1.24 (s, 2H), 0.86 (s, 2H). ^{13}C NMR (126 MHz, DMSO-d_6) δ 152.51, 149.06, 148.02, 142.74, 137.69, 130.14 (2C), 128.72, 128.71 (3C), 127.75, 126.76 (2C), 126.47 (2C), 126.07, 106.89, 61.65, 54.30 (2C), 51.66, 48.00 (2C), 44.79, 38.62, 32.41, 21.08. HRMS (ESI): m/z $[M + H]^+$ calcd for $C_{28}H_{34}N_6$ 455.2923, found 455.2916. Retention time 2.474 min, HPLC purity = 97.641%.

*5-(4-((4-methylpiperazin-1-yl)methyl)phenyl)-N-(3-morpholinopropyl)-1H-pyrazolo[3,4-b]pyridin-3-amine(**12h**)*

White solid. Yield: 60%. ^{1}H NMR (500 MHz, DMSO- d_6) δ 12.04 (s, 1H), 8.68 (d, *J* = 2.2 Hz, 1H), 8.43 (d, *J* = 2.2 Hz, 1H), 7.65 (d, *J* = 8.2 Hz, 2H), 7.43 (d, *J* = 8.0 Hz, 2H), 6.27 (s, 1H), 3.60 (s, 4H), 3.56 (s, 2H), 2.50 – 2.45 (m, 5H), 2.44 – 2.33 (m, 8H), 2.03 (d, *J* = 7.4 Hz, 2H), 1.99 (d, *J* = 5.8 Hz, 2H), 1.85 – 1.80 (m, 2H), 1.46 (dd, *J* = 13.6, 6.6 Hz, 2H). HRMS (ESI): m/z $[M + H]^+$ calcd for $C_{25}H_{35}N_7O$ 450.2981, found 450.2975. Retention time 2.124 min, HPLC purity = 99.326%.

*N-(3-(1H-imidazol-1-yl)propyl)-5-(4-((4-methylpiperazin-1-yl)methyl)phenyl)-1H-pyrazolo[3,4-b]pyridin-3-amine(**12i**)*

White solid. Yield: 58%. ^{1}H NMR (500 MHz, DMSO-d_6) δ 12.09 (s, 1H), 9.39

(s, 1H), 9.10 (s, 1H), 8.87 (d, J = 2.0 Hz, 1H), 7.94 (s, 1H), 7.82 (s, 4H), 7.71 (s, 1H), 4.43 (s, 2H), 3.72 – 3.53 (m, 8H), 3.47 (s, 2H), 3.35 (t, J = 6.1 Hz, 2H), 2.82 (s, 3H), 2.27 – 2.20 (m, 2H). ^{13}C NMR (126 MHz, DMSO-d_6) δ 151.54, 148.85 (2C), 139.06, 135.88 (2C), 132.84, 128.61, 127.16 (2C), 126.24, 122.58, 120.26, 116.01, 107.04, 58.39, 49.83 (2C), 48.17, 46.84 (2C), 42.65, 29.48 (2C). HRMS (ESI): m/z [M + H]$^+$ calcd for $C_{24}H_{30}N_8$ 431.2672, found 431.2665. Retention time 1.813 min, HPLC purity = 96.311%.

*General procedure for the synthesis of target compounds (**15a** - **15f**)*

The synthetic procedures of compounds **13a** - **13f** and **14a** - **14f** were similar to those used for compounds **11a** - **11i.** The synthetic procedures of compounds **15a** - **15f** were similar to those used for compounds **12a** - **12i.**

*3-(1H-indol-5-yl)-5-(4-((4-methylpiperazin-1-yl)methyl)phenyl)-1H-pyrazolo[3,4-b]pyridine(**15a**)*

Yellow solid. Yield: 62%. ^{1}H NMR (500 MHz, DMSO-d_6) δ 13.67 (s, 1H), 11.22 (s, 1H), 8.85 (d, J = 2.0 Hz, 1H), 8.70 (d, J = 2.0 Hz, 1H), 8.28 (s, 1H), 7.84 (dd, J = 8.5, 1.5 Hz, 1H), 7.80 (d, J = 8.1 Hz, 2H), 7.55 (d, J = 8.5 Hz, 1H), 7.45 (d, J = 8.2 Hz, 2H), 7.43 – 7.40 (m, 1H), 6.60 – 6.56 (m, 1H), 3.55 (s, 2H), 2.51 (d, J = 1.8 Hz, 8H), 2.27 (s, 3H). ^{13}C NMR (126 MHz, DMSO-d_6) δ 147.97, 141.24, 138.89, 136.87, 135.87, 131.06, 129.64, 129.59 (2C), 129.40, 128.02, 127.90 (2C), 127.19, 126.04, 120.34, 118.71, 112.22, 111.94, 101.83, 61.48, 54.37 (2C), 51.98, 45.14 (2C). HRMS (ESI): m/z [M + H]$^+$ calcd for $C_{26}H_{26}N_6$ 423.2297, found 423.2296. Retention time 2.355 min, HPLC purity = 95.266%.

*3-(5-(4-((4-methylpiperazin-1-yl)methyl)phenyl)-1H-pyrazolo[3,4-b]pyridin-3-yl)aniline(**15b**)*

Yellow solid. Yield: 68%. ^{1}H NMR (400 MHz, DMSO-d_6) δ 12.00 (s, 1H), 8.98 (d, J = 2.0 Hz, 1H), 8.87 (d, J = 2.0 Hz, 1H), 8.28 (s, 1H), 8.19 (d, J = 7.9 Hz, 1H), 8.04 (d, J = 8.2 Hz, 2H), 7.85 (d, J = 8.2 Hz, 2H), 7.67 (t, J = 7.9 Hz, 1H), 7.48 (s, 1H), 4.53 (s, 2H), 3.99 (s, 8H), 3.49 (s, 2H), 2.83 (s, 3H). ^{13}C NMR (126 MHz, DMSO-d_6) δ 152.99, 148.93, 142.24, 139.25, 134.73, 133.20, 130.98 (2C), 129.74 (2C), 129.51, 128.25 (2C), 128.15, 126.44, 123.42, 121.82, 112.55, 58.43, 49.82 (2C), 48.25 (2C), 42.63. HRMS (ESI): m/z [M + H]$^+$ calcd for $C_{24}H_{26}N_6$ 399.2297, found 399.2289. Retention time 1.938 min, HPLC purity = 98.289%.

*4-(5-(4-((4-methylpiperazin-1-yl)methyl)phenyl)-1H-pyrazolo[3,4-b]pyridin-3-yl)aniline(**15c**)*

Yellow solid. Yield: 68%. ^{1}H NMR (400 MHz, DMSO-d_6) δ 11.95 (s, 1H), 8.95 (s, 1H), 8.80 (s, 1H), 8.26 (d, J = 8.3 Hz, 2H), 7.99 (d, J = 7.7 Hz, 2H), 7.85 (d, J = 8.1 Hz, 2H), 7.59 (d, J = 8.5 Hz, 2H), 4.53 (s, 2H), 3.65 (d, J = 19.5 Hz, 8H), 3.50 (s, 2H), 2.84 (s, 3H). ^{13}C NMR (126 MHz, DMSO-d_6) δ 152.95, 148.83, 142.52, 139.40, 133.04, 132.54, 132.22, 131.92, 129.64 (2C), 128.82 (2C), 128.52 (2C), 128.34, 128.14, 124.37, 112.56, 58.63, 48.96 (2C), 48.13 (2C), 42.67. HRMS (ESI): m/z $[M + H]^+$ calcd for $C_{24}H_{26}N_6$ 399.2297, found 399.2290. Retention time 1.899 min, HPLC purity = 97.869%.

*3-(1H-benzo[d]imidazol-5-yl)-5-(4-((4-methylpiperazin-1-yl)methyl)phenyl)-1H-pyrazolo[3,4-b]pyridine(**15d**)*

Yellow solid. Yield: 62%. ^{1}H NMR (400 MHz, DMSO-d_6) δ 14.14 (s, 1H), 12.10 (s, 1H), 9.68 (s, 1H), 8.87 (d, J = 62.5 Hz, 2H), 8.42 (d, J = 65.1 Hz, 2H), 7.96 (s, 2H), 7.85 (s, 2H), 7.43 (s, 1H), 6.81 (s, 1H), 4.40 (d, J = 99.0 Hz, 8H), 3.37 (s, 2H), 2.82 (s, 3H). ^{13}C NMR (126 MHz, DMSO-d_6) δ 159.08, 152.98, 148.93, 142.61, 141.46, 139.32, 134.90, 133.45, 132.72, 131.91, 131.65, 129.68, 128.95, 128.21, 127.81, 125.53, 115.99, 115.57, 112.54, 63.26, 58.57, 49.74 (2C), 47.99 (2C). HRMS (ESI): m/z $[M + H]^+$ calcd for $C_{25}H_{25}N_7$ 424.2250, found 424.2244. Retention time 1.98 min, HPLC purity = 98.548%.

*3-(1-isopropyl-1H-benzo[d]imidazol-6-yl)-5-(4-((4-methylpiperazin-1-yl)methyl)phenyl)-1H-pyrazolo[3,4-b]pyridine(**15e**)*

Yellow solid. Yield: 61%. ^{1}H NMR (400 MHz, DMSO-d_6) δ 13.87 (s, 1H), 8.88 (s, 1H), 8.72 (s, 1H), 8.44 (s, 1H), 8.22 (s, 1H), 7.96 (d, J = 8.6 Hz, 1H), 7.83 – 7.76 (m, 3H), 7.43 (d, J = 8.0 Hz, 2H), 5.00 – 4.91 (m, 1H), 3.51 (s, 2H), 2.38 (s, 8H), 2.16 (s, 3H), 1.59 (d, J = 6.7 Hz, 6H). ^{13}C NMR (126 MHz, DMSO-d_6) δ152.46, 148.23, 143.91, 143.68, 142.40, 137.60, 136.69, 133.72, 129.65 (2C), 129.61, 127.82, 127.39 (2C), 127.14, 121.06, 119.91, 112.24, 108.87, 61.64, 54.69 (2C), 52.50, 46.90 (2C), 45.66, 22.34 (2C). HRMS (ESI): m/z $[M + H]^+$ calcd for $C_{28}H_{31}N_7$ 466.2719, found 466.2715. Retention time 2.159 min, HPLC purity = 100%.

*6-(5-(4-((4-methylpiperazin-1-yl)methyl)phenyl)-1H-pyrazolo[3,4-b]pyridin-3-yl)isoquinoline(**15f**)*

Yellow solid. Yield: 50%. ^{1}H NMR (400 MHz, DMSO-d_6) δ 12.06 (s, 1H), 10.08 (s, 1H), 9.03 (d, J = 2.0 Hz, 1H), 9.00 (d, J = 6.7 Hz, 1H), 8.75 (d, J = 6.8 Hz, 1H), 8.69 (t, J = 7.9 Hz, 2H), 8.60 (d, J = 2.0 Hz, 1H), 8.21 (d, J = 7.8 Hz, 1H), 7.94 (d, J = 8.2 Hz, 2H), 7.83 (d, J = 8.1 Hz, 2H), 4.53 – 4.46 (m, 2H), 3.63 (d, J = 24.2 Hz, 8H), 2.82 (s, 3H). ^{13}C NMR (126 MHz, DMSO-d_6) δ 152.63, 149.41, 148.27, 141.43, 139.22, 137.60, 136.39, 133.33, 132.70, 131.27, 131.02, 130.04, 129.79 (2C), 129.01, 128.64, 128.21 (2C), 128.09, 123.73, 114.17, 58.43, 49.78 (2C), 48.14 (2C), 42.73. HRMS (ESI): m/z $[M + H]^+$ calcd for $C_{27}H_{26}N_6$ 435.2297, found 435.2294. Retention time 1.973 min, HPLC purity = 98.027%.

*5-bromo-N-isopropylbenzene-1,2-diamine(**17**)*

A well stirred mixture of **16** (4.64 g, 18 mol), reduced iron powder (6.05 g, 108 mmol), and ammonium chloride (2.91 g, 54 mmol) in ethanol and water (30 mL, v/v=3/l) was heated to 80 °C for 2 h. After cooling to room temperature, it was filtered on Celite, the filtrate was concentrated under reduced pressure to give a brown oil in 93% yield, which was used in the next step without further purification.

*6-bromo-1-isopropyl-1H-benzo[d]imidazole (**18**)*

Compound **17** (1.50 g, 6.57 mmol) was added into formic acid (50 mL), and the reaction was refluxed for 6 h. After cooling to room temperature, formic acid was removed under reduce pressure, the pH of the residue was adjusted to about 7 with saturated solution of sodium bicarbonate, extracted with dichloromethane (50×3). The organic layer was combined, washed with brine, dried over anhydrous sodium sulfate. The filtrate was concentrated under reduced pressure, and the filtrate was separated on column chromatography (dichloromethane/methanol (v/v) = 50 : l) to afford a brown-yellow solid in 92% yield.

*isopropyl-6-(4,4,5,5-tetramethyl-1,3,2-dioxaborolan-2-yl)-1H-benzo[d]imidazole(**19**)*

Under nitrogen protection, Pd(dppf)Cl_2 (74 mg, 0.10 mmol) was added into the mixture of Compound **18** (600 mg, 2.10 mmol), bis(pinacolato)diboron (2.20 g, 8.86 mmol) and potassium acetate (1.70 g, 17.72 mmol) in anhydrous 1,4-dioxane (20 mL), and the reaction was performed at 100 °C for 10 h. After cooling to room temperature, the reaction was quenched by adding water, extracted with ethyl acetate (100 mL×3), the organic layer was combined, washed with brine, dried over anhydrous sodium sulfate, concentrated under reduced pressure, and the filtrate was

separated on column chromatography (petroleum ether/ethyl acetate (v/v) = 2 : 1), to afford a yellow oil in 67% yield.

*General procedure for the synthesis of target compounds (**15g** - **15k**)*

The synthetic procedures of compounds **20** and **21g** - **21k** were similar to those used for compounds **11a** - **11i.** The synthetic procedures of compounds **15g** - **15k** were similar to those used for compounds **12a** - **12i.**

*(4-(3-(1-isopropyl-1H-benzo[d]imidazol-6-yl)-1H-pyrazolo[3,4-b]pyridin-5-yl)phenyl)(4-methylpiperazin-1-yl)methanone(**15g**)*

Yellow solid. Yield: 46%. ^{1}H NMR (400 MHz, DMSO-d_6) δ 13.91 (s, 1H), 8.92 (s, 1H), 8.79 (s, 1H), 8.45 (s, 1H), 8.23 (s, 1H), 8.00 – 7.92 (m, 3H), 7.81 (d, J = 8.5 Hz, 1H), 7.54 (d, J = 8.0 Hz, 2H), 4.99 – 4.93 (m, 1H), 3.64 (s, 4H), 2.33 (s, 4H), 2.21 (s, 3H), 1.59 (d, J = 6.6 Hz, 6H). ^{13}C NMR (126 MHz, DMSO-d_6) δ 168.69, 152.57, 148.26, 144.13, 143.72, 142.43, 139.15, 134.85, 133.73, 128.99, 128.36 (2C), 127.71 (2C), 127.34, 121.09, 119.91, 114.84, 112.23, 108.89, 54.54, 46.90 (2C), 45.58 (2C), 35.08, 22.35 (2C). HRMS (ESI): m/z [M + H]$^+$ calcd for $C_{28}H_{29}N_7O$ 480.2512, found 480.2509. Retention time 2.178 min, HPLC purity = 99.516%.

*3-(3-(1-isopropyl-1H-benzo[d]imidazol-6-yl)-1H-pyrazolo[3,4-b]pyridin-5-yl)benzamide(**15h**)*

Yellow solid. Yield: 50%. ^{1}H NMR (400 MHz, DMSO-d_6) δ 14.18 (s, 1H), 9.85 (s, 1H), 9.00 (d, J = 1.9 Hz, 1H), 8.87 (d, J = 2.0 Hz, 1H), 8.63 (s, 1H), 8.38 (d, J = 8.6 Hz, 1H), 8.12 (s, 1H), 8.05 (t, J = 7.8 Hz, 3H), 7.97 (d, J = 8.4 Hz, 2H), 7.46 (s, 1H), 5.33 – 5.26 (m, 1H), 1.68 (d, J = 6.6 Hz, 6H). ^{13}C NMR (126 MHz, DMSO-d_6) δ 167.92, 153.09, 149.16, 142.97, 141.06, 140.82, 133.59, 131.84, 131.68, 131.33, 129.77 (2C), 128.79 (2C), 127.65, 126.75, 126.23, 116.11, 112.63, 111.69, 50.39, 22.31 (2C). HRMS (ESI): m/z [M + H]$^+$ calcd for $C_{23}H_{20}N_6O$ 397.1777, found 397.1775. Retention time 2.344 min, HPLC purity = 96.851%.

*4-(3-(1-isopropyl-1H-benzo[d]imidazol-6-yl)-1H-pyrazolo[3,4-b]pyridin-5-yl)benzenesulfonamide(**15i**)*

Yellow solid. Yield: 43%. ^{1}H NMR (400 MHz, DMSO-d_6) δ 13.96 (s, 1H), 8.96 (d, J = 1.9 Hz, 1H), 8.83 (d, J = 1.9 Hz, 1H), 8.48 (s, 1H), 8.25 (s, 1H), 8.07 (d, J = 8.5 Hz, 2H), 7.98 (d, J = 9.8 Hz, 1H), 7.96 (t, J = 5.3 Hz, 2H), 7.83 (d, J = 8.3 Hz, 1H), 7.46 (s, 2H), 5.03 – 4.92 (m, 1H), 1.60 (d, J = 6.7 Hz, 6H). ^{13}C NMR (126 MHz, DMSO-d_6) δ 154.59, 150.23, 146.15, 144.85, 144.36, 143.29, 135.58, 131.53,

130.73, 130.30, 129.71 (2C), 129.25, 128.22 (2C), 123.14, 121.78, 114.16, 110.96, 48.92, 24.24 (2C). HRMS (ESI): m/z $[M + H]^+$ calcd for $C_{22}H_{20}N_6O_2S$ 433.1447, found 433.1446. Retention time 2.348 min, HPLC purity = 100%.

*N-(6-(3-(1-isopropyl-1H-benzo[d]imidazol-6-yl)-1H-pyrazolo[3,4-b]pyridin-5-yl)pyridin-2-yl)acetamide(**15j**)*

Yellow solid. Yield: 42%. ^{1}H NMR (400 MHz, DMSO-d_6) δ 14.38 (s, 1H), 11.56 (s, 1H), 9.91 (d, J = 3.7 Hz, 1H), 8.98 (d, J = 2.0 Hz, 1H), 8.94 (d, J = 2.0 Hz, 1H), 8.62 (s, 1H), 8.46 (d, J = 5.2 Hz, 2H), 8.34 (dd, J = 8.6, 1.2 Hz, 1H), 8.04 (d, J = 8.6 Hz, 1H), 7.84 (d, J = 5.6 Hz, 1H), 5.34 – 5.28 (m, 1H), 2.21 (s, 3H), 1.68 (d, J = 6.6 Hz, 6H). ^{13}C NMR (126 MHz, DMSO-d_6) δ 170.41, 153.13, 151.14, 149.38, 148.27, 145.30, 142.92, 140.39, 131.14, 131.04, 130.67, 129.30, 126.66, 125.73, 117.78, 115.61, 112.09, 111.61, 111.36, 50.01, 23.98, 21.83 (2C). HRMS (ESI): m/z $[M + H]^+$ calcd for $C_{23}H_{21}N_7O$ 412.1886, found 412.1874. Retention time 2.146 min, HPLC purity = 95.569%.

*Ethyl-6-(3-(1-isopropyl-1H-benzo[d]imidazol-6-yl)-1H-pyrazolo[3,4-b]pyridin-5-yl)picolinate(**15k**)*

Yellow solid. Yield: 43%. ^{1}H NMR (400 MHz, DMSO-d_6) δ 14.20 (s, 1H), 9.81 (s, 1H), 8.96 (d, J = 1.9 Hz, 1H), 8.85 (d, J = 1.9 Hz, 1H), 8.62 (s, 1H), 8.35 (d, J = 4.5 Hz, 2H), 8.16 (d, J = 7.9 Hz, 1H), 8.05 – 8.01 (m, 2H), 7.71 (t, J = 7.7 Hz, 1H), 5.31 – 5.25 (m, 1H), 3.91 (s, 3H), 1.68 (d, J = 6.6 Hz, 6H). ^{13}C NMR (126 MHz, DMSO-d_6) δ 166.12, 152.58, 148.58, 142.54, 140.42, 138.54, 132.35, 131.51, 131.27, 130.85, 130.50, 129.61, 129.16, 128.43, 128.17, 127.90, 125.57, 115.76, 112.11, 111.17, 52.30, 49.82, 21.78 (2C). HRMS (ESI): m/z $[M + H]^+$ calcd for $C_{24}H_{21}N_5O_2$ 412.1773, found 412.1770. Retention time 2.816 min, HPLC purity = 96.032%.

*N-cyclopropylacetamide(**23**)*

In an ice bath, acetic anhydride (16 mL) was slowly added into the solution of triethylamine (23 mL) and compund **22** (9 g) in dichloromethane (100 mL). After the addition was completed, the reaction was stirred overnight at r.t.. The dichloromethane was removed under reduced pressure. Diethyl ether was added, followed by a large amount of potassium carbonate, and the mixture was stirred 10 h. The solid was filtered, and the filtrate was concentrated under reduced pressure to give a colorless clear oil in 95% yield.

*(E)-N'-(4-bromo-2,6-difluorophenyl)-N-cyclopropylacetimidamide (**24**)*

At room temperature, phosphorus oxychloride (6.70 mL) was added into the solution of 4-bromo-2,6-difluoroaniline (10.0 g, 48.00 mmol) and compound **23** (9.50 g, 96.00 mmol), triethylamine (10 mL) in toluene (150 mL), and the reaction mixture was stirred under reflux for 3 hours. After cooling to room temperature, the reaction solvent was removed under reduced pressure. The crude was dissolved into 150 mL dichloromethane, washed with saturated sodium bicarbonate for three times, and dried over anhydrous sodium sulfate. The filtrate was concentrated under reduced pressure, and the filtrate was separated on column chromatography (petroleum ether/ethyl acetate (v/v) = 5 : l), to afford a white solid. Yield: 86%.

*6-bromo-1-cyclopropyl-4-fluoro-2-methyl-1H-benzo[d]imidazole(**25**)*

Under nitrogen protection, potassium tert-butoxide (7.00 g, 62.5 mmol) was added into compound **24** (12 g, 41.7 mmol) in anhydrous tetrahydrofuran (100 mL), and the reaction was reacted at 80 °C for 6 h. After cooling to room temperature, it was filtered on Celite, and the filter cake was washed with dichloromethane. The filtrate was washed with brine, dried over anhydrous sodium sulfate. The filtrate was concentrated under reduced pressure, and the filtrate was separated on column chromatography (petroleum ether/ethyl acetate (v/v) = 3 : 1), to afford a pale yellow solid. Yield: 73%.

*cyclopropyl-4-fluoro-2-methyl-6-(4,4,5,5-tetramethyl-1,3,2-dioxaborolan-2-yl)-1H-benzo[d]imidazole(**26**)*

The synthesis of intermediate **26** was similar to that of compound **19**. ^{1}H NMR (400 MHz, $CDCl_3$) δ 7.71 (s, 1H), 7.35 (d, *J* = 10.7 Hz, 1H), 4.71 (dd, *J* = 13.9, 6.9 Hz, 1H), 2.68 (s, 3H), 1.68 (d, *J* = 6.6 Hz, 4H), 1.39 (s, 12H).

*N-(4-bromo-2,6-difluorophenyl)acetamide(**28**)*

At room temperature, acetic anhydride (9 mL) was added dropwise to compound **27** (3.00 g, 14.42 mmol) in glacial acetic acid (30 mL), and the reaction was reacted at room temperature for 5 h. It was added into ice-water, the solid was filtered, the filter cake was dissolved in dichloromethane, washed with saturated solution of sodium bicarbonate and brine, dried over anhydrous sodium sulfate, concentrated under reduced pressure to afford a yellow solid in 81% yield.

*6-bromo-4-fluoro-2-methylbenzo[d]oxazole(**29**)*

Cesium carbonate (8.00 g) was added into compound **28** (3.00 g) in anhydrous NMP (19 mL), and the reaction was refluxed at 150 °C for 10 h. After cooling to room temperature, the reaction was quenched by adding water, extracted with ethyl acetate (50 mL×3). The combined organic layer was washed with water and brine, dried over anhydrous sodium sulfate, concentrated under reduced pressure, and the concentrate was separated by column chromatography (petroleum ether/ethyl acetate (v/v) = 9 : l) to afford a pale yellow solid in 45% yield.

*4-fluoro-2-methyl-6-(4,4,5,5-tetramethyl-1,3,2-dioxaborolan-2-yl)benzo[d]oxazole (**30**)*

The synthesis of intermediate **30** was similar to that of compound **19**. ^{1}H NMR (400 MHz, $CDCl_3$) δ 7.96 (s, 1H), 7.71 (d, J = 10.7 Hz, 1H), 2.72 (s, 3H), 1.33 (s, 12H).

*6-bromo-4-fluoro-lH-indazole(**32**)*

At room temperature, hydrazine hydrate (10 mL) was added into the compound **31** (5.00 g, 22.60 mmol) in 1,4-dioxane (10 mL), and the reaction was reacted at 90 °C for 5 h. After cooling to room temperature, it was added into the mixture of ice and water, extracted with ethyl acetate (50 mL×3). The organic layer was combined, dried over anhydrous sodium sulfate, and concentrated under reduced pressure to afford a yellow solid in 62% yield.

*6-bromo-4-fluoro-1-((2-(trimethylsilyl)ethoxy)methyl)-1H-indazole(**33**)*

In an ice bath, NaH (1.00 g, 41.86 mmol) was slowly in batches added into the solution of intermediate **32** (3.00 g, 13.95 mmol) in dry DMF (20 mL). After the addition was completed, the reaction was stirred 15 min. And then SEM-Cl (2.79 g, 16.74 mmol) was added followed by stirred 10 h. After cooling to room temperature, it was added into water (200 mL) and A large number of white precipitates were precipitated. The solid was filtered to afford a white solid in 92% yield.

*4-fluoro-6-(4,4,5,5-tetramethyl-1,3,2-dioxaborolan-2-yl)-1-((2-(trimethylsilyl)ethoxy)methyl)-1H-indazole(**34**)*

The synthesis of intermediate **34** was similar to that of compound **19**. ^{1}H NMR (400 MHz, $CDCl_3$) δ 8.16 (d, *J* = 0.7 Hz, 1H), 7.70 (t, *J* = 1.1 Hz, 1H), 6.86 (dd, *J* = 9.5, 1.2 Hz, 1H), 5.69 (s, 2H), 3.65 – 3.60 (m, 2H), 1.37 (s, 12H), 0.94 (dd, *J* = 8.9, 7.7 Hz, 2H), -0.03 (s, 9H).

*2-amino-4-bromo-6-fluorophenol(**36**)*

The synthesis of intermediate **36** was similar to that of compound **17**.

*5-bromo-7-fluoro-2-methylbenzo[d]oxazole(**37**)*

Compound **36** (700 mg, 3.41mmol) was added into triethyl orthoacetate (20 mL), and the reaction was refluxed at 150 °C for 6 h. After cooling to room temperature, the reaction was quenched by adding saturated solution of sodium bicarbonate, extracted with ethyl acetate (50 mL×3), the combined organic layer was washed with water and brine, dried over anhydrous sodium sulfate, concentrated under reduced pressure, and the concentrate was separated by column chromatography (petroleum ether/ethyl acetate (v/v) = 1 : 1), to afford a pale yellow solid in 68% yield.

*7-fluoro-2-methyl-5-(4,4,5,5-tetramethyl-1,3,2-dioxaborolan-2-yl)benzo[d]oxazole(**38**)*

The synthesis of intermediate **38** was similar to that of compound **19**. ^{1}H NMR (400 MHz, $CDCl_3$) δ 7.87 (s, 1H), 7.49 (d, *J* = 10.2 Hz, 1H), 2.67 (s, 3H), 1.36 (s, 12H).

*5-bromo-3-fluorobenzene-1,2-diamine(**40**)*

The synthesis of intermediate **40** was similar to that of compound **17**.

*6-bromo-4-fluoro-2-(trifluoromethyl)-1H-benzo[d]imidazole(**41**)*

Compound **40** (920mg, 4.01 mmol) was added into trifluoroacetic acid (20 mL), and the reaction was reacted at 70 °C for 4 h. After cooling to room temperature,

trifluoroacetic acid was removed under reduce pressure, the pH of the residue was adjusted to about 7 with saturated solution of sodium bicarbonate, extracted with dichloromethane (50×3). The organic layer was combined, washed with brine, dried over anhydrous sodium sulfate. The filtrate was concentrated under reduced pressure, and the filtrate was separated on column chromatography (dichloromethane/methanol (v/v) = 20 : l) to afford a pale yellow solid in 70% yield.

*6-bromo-4-fluoro-1-isopropyl-2-(trifluoromethyl)-1H-benzo[d]imidazole(**42**)*

The synthesis of intermediate **42** was similar to that of compound **33**.

*4-fluoro-1-isopropyl-6-(4,4,5,5-tetramethyl-1,3,2-dioxaborolan-2-yl)-2-(trifluoromethyl)-1H-benzo[d]imidazole(**43**)*

The synthesis of intermediate **43** was similar to that of compound **19**. ^{1}H NMR (400 MHz, $CDCl_3$) δ 7.88 (s, 1H), 7.44 (d, *J* = 10.5 Hz, 1H), 4.98 – 4.90 (m, 1H), 1.73 (d, *J* = 6.9 Hz, 6H), 1.35 (s, 12H).

*General procedure for the synthesis of target compounds (**15l** - **15ab**)*

The synthetic procedures of compounds **44l** - **44w, 45l** - **45w, 49x** - **49ab and 50x** - **50ab** were similar to those used for compounds **11a** - **11i.** The synthetic procedures of compounds **15l** - **15ab** were similar to those used for compounds **12a** - **12i.**

*4-(3-(1-(tetrahydro-2H-pyran-4-yl)-1H-pyrazol-4-yl)-1H-pyrazolo[3,4-b]pyridin-5-yl)benzenesulfonamide(**15l**)*

Yellow solid. Yield: 38%. ^{1}H NMR (400 MHz, DMSO-*d*$_6$) δ 8.92 (d, *J* = 2.1 Hz, 1H), 8.81 (d, *J* = 2.1 Hz, 1H), 8.62 (s, 1H), 8.16 (s, 1H), 8.06 (d, *J* = 8.5 Hz, 2H), 7.95 (d, *J* = 8.4 Hz, 2H), 7.47 (s, 2H), 4.51 (dd, *J* = 10.3, 5.1 Hz, 1H), 4.01 (d, *J* = 11.0 Hz, 2H), 3.51 (td, *J* = 11.4, 3.2 Hz, 2H), 2.12 – 2.03 (m, 4H). ^{13}C NMR (126 MHz, DMSO-*d*$_6$) δ 152.62, 148.70, 143.39, 141.70, 138.32, 136.83, 128.86, 128.40, 128.23 (2C), 126.72 (2C), 126.55, 114.61, 112.46, 66.47 (2C), 58.00, 33.42 (2C). HRMS (ESI): m/z $[M + H]^+$ calcd for $C_{20}H_{20}N_6O_3S$ 425.1396, found 425.1394. Retention time 2.501 min, HPLC purity = 95.175%.

*4-(3-(4-morpholinophenyl)-1H-pyrazolo[3,4-b]pyridin-5-yl)benzenesulfonamide(**15m**)*

Yellow solid. Yield: 37%. ^{1}H NMR (400 MHz, DMSO-d_6) δ 8.94 (d, J = 2.0 Hz, 1H), 8.79 (d, J = 2.0 Hz, 1H), 8.06 (dd, J = 8.5, 4.5 Hz, 4H), 7.95 (d, J = 8.5 Hz, 2H), 7.47 (s, 2H), 7.27 (d, J = 8.3 Hz, 2H), 3.86 – 3.83 (m, 4H), 3.30 (d, J = 4.3 Hz, 4H). ^{13}C NMR (126 MHz, DMSO-d_6) δ 153.06, 148.63, 143.66, 143.42, 141.70, 132.53, 131.95 (2C), 129.23 (2C), 128.65 (2C), 128.20 (2C), 128.05, 126.76, 116.74, 112.52, 66.12 (2C), 49.50 (2C). HRMS (ESI): m/z [M + H]$^+$ calcd for $C_{22}H_{21}N_5O_3S$ 436.1443, found 436.1441. Retention time 2.133 min, HPLC purity = 96.233%.

*4-(3-(1-(2-morpholinoethyl)-1H-pyrazol-4-yl)-1H-pyrazolo[3,4-b]pyridin-5-yl)benzenesulfonamide(**15n**)*

Yellow solid. Yield: 38%. ^{1}H NMR (400 MHz, DMSO-d_6) δ 8.92 (d, J = 2.1 Hz, 1H), 8.75 (d, J = 2.0 Hz, 1H), 8.58 (s, 1H), 8.14 (s, 1H), 8.06 (d, J = 8.3 Hz, 2H), 7.95 (d, J = 8.5 Hz, 2H), 7.70 (dd, J = 22.2, 5.7 Hz, 1H), 7.24 (s, 2H), 4.33 (t, J = 6.6 Hz, 2H), 4.23 (t, J = 6.5 Hz, 1H), 3.57 – 3.55 (m, 4H), 2.80 (t, J = 6.6 Hz, 2H), 2.45 (s, 4H). ^{13}C NMR (126 MHz, DMSO-d_6) δ 152.70, 148.73, 143.37, 141.70, 138.38, 137.14, 129.13 (2C), 128.64 (2C), 128.35, 128.18, 126.72, 114.55, 112.44, 66.84 (2C), 60.52, 54.68, 49.02 (2C). HRMS (ESI): m/z [M + H]$^+$ calcd for $C_{21}H_{23}N_7O_3S$ 454.1661, found 454.1656. Retention time 2.287 min, HPLC purity = 98.364%.

*4-(3-(1-(2-hydroxyethyl)-1H-pyrazol-4-yl)-1H-pyrazolo[3,4-b]pyridin-5-yl)benzenesulfonamide(**15o**)*

Yellow solid. Yield: 39%. ^{1}H NMR (400 MHz, DMSO-d_6) δ 8.92 (d, J = 2.0 Hz, 1H), 8.78 (d, J = 2.0 Hz, 1H), 8.55 (s, 1H), 8.17 (s, 1H), 8.07 (d, J = 8.4 Hz, 2H), 7.95 (d, J = 8.4 Hz, 2H), 7.46 (s, 2H), 4.26 (t, J = 5.6 Hz, 2H), 3.83 (s, 2H). ^{13}C NMR (126 MHz, DMSO-d_6) δ 152.70, 148.73, 143.37, 141.70, 138.38, 137.14, 129.13 (2C), 128.64 (2C), 128.35, 128.18, 126.72, 114.55, 112.44, 60.52, 54.68. HRMS (ESI): m/z [M + H]$^+$ calcd for $C_{17}H_{16}N_6O_3S$ 385.1083, found 385.1073. Retention time 2.225 min, HPLC purity = 98.209%.

*4-(3-(4-fluoro-2-methylbenzo[d]oxazol-6-yl)-1H-pyrazolo[3,4-b]pyridin-5-yl)benzenesulfonamide(**15p**)*

Yellow solid. Yield: 40%. ^{1}H NMR (400 MHz, DMSO-d_6) δ 8.97 (d, J = 1.9 Hz, 1H), 8.88 (s, 1H), 8.12 (d, J = 8.0 Hz, 2H), 7.97 (t, J = 12.0 Hz, 3H), 7.84 (s, 1H), 7.48 (s, 2H), 2.68 (s, 3H). HRMS (ESI): m/z [M + H]$^+$ calcd for $C_{21}H_{15}N_4O_3S$ 423.0927, found 423.0925. Retention time 2.121 min, HPLC purity = 96.315%.

*4-(3-(4-fluoro-1H-indazol-6-yl)-1H-pyrazolo[3,4-b]pyridin-5-yl)benzenesulfonamide(**15q**)*

Yellow solid. Yield: 35%. ^{1}H NMR (400 MHz, DMSO-d_6) δ 14.11 (s, 1H), 8.98 (d, J = 1.9 Hz, 1H), 8.88 (d, J = 2.0 Hz, 1H), 8.26 (s, 1H), 8.11 (d, J = 7.0 Hz, 2H), 8.08 (s, 1H), 7.97 (d, J = 8.4 Hz, 2H), 7.64 (d, J = 11.4 Hz, 1H), 7.48 (s, 2H). ^{13}C NMR (126 MHz, DMSO-d_6) δ 155.51 (d, J = 249.5 Hz), 153.14, 149.02, 143.56, 143.10, 141.65, 132.50, 130.16, 129.16 (2C), 128.34 (3C), 126.78, 113.15, 112.64, 105.45, 104.08, 103.92. HRMS (ESI): m/z [M + H]$^+$ calcd for $C_{19}H_{13}FN_6O_2S$ 409.0883, found 409.0882. Retention time 2.696 min, HPLC purity = 98.347%.

*4-(3-(7-fluoro-2-methylbenzo[d]oxazol-5-yl)-1H-pyrazolo[3,4-b]pyridin-5-yl)benzenesulfonamide(**15r**)*

Yellow solid. Yield: 36%. ^{1}H NMR (400 MHz, DMSO-d_6) δ 8.97 (d, J = 1.9 Hz, 1H), 8.88 (s, 1H), 8.12 (d, J = 8.0 Hz, 2H), 7.97 (t, J = 12.0 Hz, 3H), 7.84 (s, 1H), 7.48 (s, 2H), 2.68 (s, 3H). HRMS (ESI): m/z [M + H]$^+$ calcd for $C_{21}H_{15}N_4O_3S$ 423.0927, found 423.0924. Retention time 2.201 min, HPLC purity = 96.634%.

*4-(3-(1-isopropyl-2-methyl-1H-benzo[d]imidazol-6-yl)-1H-pyrazolo[3,4-b]pyridin-5-yl)benzenesulfonamide(**15s**)*

Yellow solid. Yield: 39%. ^{1}H NMR (400 MHz, DMSO-d_6) δ 8.98 (d, J = 2.0 Hz, 1H), 8.80 (d, J = 2.0 Hz, 1H), 8.30 (s, 1H), 8.15 – 8.09 (m, 3H), 8.05 (s, 1H), 7.96 (d, J = 8.5 Hz, 2H), 7.48 (s, 2H), 5.11 – 5.05 (m, 1H), 2.90 (s, 3H), 1.70 (d, J = 6.9 Hz, 6H). ^{13}C NMR (126 MHz, DMSO-d_6) δ 154.39, 152.35, 149.40, 142.97, 142.63, 137.72, 130.14, 129.48 (2C), 127.83 (2C), 126.82, 126.20, 125.75, 117.94, 115.46, 106.47, 106.33, 105.96, 48.19, 21.36(2C), 15.14. HRMS (ESI): m/z [M + H]+ calcd for $C_{23}H_{22}N_6O_2S$ 447.1603, found 447.1613. Retention time 2.411 min, HPLC purity = 96.286%.

*4-(3-(4-fluoro-1-isopropyl-2-methyl-1H-benzo[d]imidazol-6-yl)-1H-pyrazolo[3,4-b]pyridin-5-yl)benzenesulfonamide(**15t**)*

Yellow solid. Yield: 45%. ^{1}H NMR (400 MHz, DMSO-d_6) δ 8.99 (d, *J* = 2.0 Hz, 1H), 8.82 (d, *J* = 2.0 Hz, 1H), 8.31 (s, 1H), 8.10 (d, *J* = 4.4 Hz, 2H), 8.07 (s, 1H), 7.97 (d, *J* = 8.5 Hz, 2H), 7.49 (s, 2H), 5.11 – 5.05 (m, 1H), 2.90 (s, 3H), 1.70 (d, *J* = 6.9 Hz, 6H). ^{13}C NMR (126 MHz, DMSO-d_6) δ 153.41, 153.12, 151.35, 149.38(d, *J* = 248.2 Hz), 149.18, 143.57, 142.56, 141.66, 134.77, 130.85, 129.24, 129.10 (2C), 128.31 (2C), 126.80, 112.54, 109.94, 107.95, 50.41, 20.90 (2C), 13.57. HRMS (ESI): m/z [M + H]$^+$ calcd for $C_{23}H_{21}FN_6O_2S$ 465.1509, found 465.1501. Retention time 2.416 min, HPLC purity = 100%.

*4-(3-(1-ethyl-1H-benzo[d]imidazol-6-yl)-1H-pyrazolo[3,4-b]pyridin-5-yl)benzenesulfonamide(**15u**)*

Yellow solid. Yield: 48%. ^{1}H NMR (400 MHz, DMSO-d_6) δ 9.79 (s, 1H), 8.99 (d, *J* = 2.1 Hz, 1H), 8.91 (d, *J* = 2.1 Hz, 1H), 8.61 (d, *J* = 0.6 Hz, 1H), 8.37 (d, *J* = 1.4 Hz, 1H), 8.09 (d, *J* = 1.9 Hz, 1H), 8.07 (d, *J* = 1.9 Hz, 2H), 7.98 (s, 2H), 7.50 – 7.45 (m, 2H), 4.68 (d, *J* = 7.3 Hz, 2H), 1.61 (t, *J* = 7.3 Hz, 3H). ^{13}C NMR (126 MHz, DMSO-d_6) δ 153.12, 149.12, 143.54, 142.96, 142.16, 141.64, 141.53, 131.97, 131.37, 129.22 (2C), 128.30 (2C), 126.77, 126.11, 116.03, 114.35, 112.56, 111.50, 42.31, 14.84. HRMS (ESI): m/z [M + H]$^+$ calcd for $C_{21}H_{18}N_6O_2S$ 419.1290, found 419.1289. Retention time 2.287 min, HPLC purity = 98.364%.

*4-(3-(1-cyclopropyl-4-fluoro-2-methyl-1H-benzo[d]imidazol-6-yl)-1H-pyrazolo[3,4-b]pyridin-5-yl)benzenesulfonamide(**15v**)*

Yellow solid. Yield: 44%. ^{1}H NMR (400 MHz, DMSO-d_6) δ 8.97 (d, *J* = 2.0 Hz, 1H), 8.82 (d, *J* = 2.1 Hz, 1H), 8.09 (d, *J* = 8.5 Hz, 2H), 8.02 (d, *J* = 1.3 Hz, 1H), 7.96 (d, *J* = 8.6 Hz, 2H), 7.74 (s, 1H), 7.44 (s, 2H), 3.45 (s, 1H), 2.65 (d, *J* = 5.1 Hz, 3H), 1.49 – 1.43 (m, 4H). ^{13}C NMR (126 MHz, DMSO-d_6) δ 156.34, 153.07, 149.01(d, *J* = 239.4 Hz), 145.48, 142.21, 139.05, 137.11, 131.95, 131.18, 130.26, 129.45 (2C), 129.19, 128.78 (2C), 124.95, 112.50, 109.89, 107.07, 26.66, 13.55, 6.77 (2C). HRMS (ESI): m/z [M + H]$^+$ calcd for $C_{23}H_{19}N_6O_2S$ 463.1352, found 463.1351 min. Retention time 2.399, HPLC purity = 98.685%.

*4-(3-(4-fluoro-1-isopropyl-2-(trifluoromethyl)-1H-benzo[d]imidazol-6-yl)-1H-pyrazolo[3,4-b]pyridin-5-yl)benzenesulfonamide(**15w**)*

Yellow solid. Yield: 46%. ^{1}H NMR (400 MHz, DMSO-d_6) δ 14.18 (s, 1H), 9.03 (d, *J* = 2.0 Hz, 1H), 8.84 (d, *J* = 2.0 Hz, 1H), 8.40 (s, 1H), 8.31 (s, 1H), 8.25 (d, *J* = 7.9 Hz, 1H), 7.96 (dd, *J* = 12.3, 9.9 Hz, 2H), 7.82 (t, *J* = 7.8 Hz, 1H), 7.21 (s, 2H), 5.01 (dt, *J* = 13.7, 6.8 Hz, 1H), 1.74 (d, *J* = 6.9 Hz, 6H). ^{13}C NMR (126 MHz, DMSO-d_6) δ 153.13, 149.02 (d, *J* = 236.9 Hz), 143.55, 142.89, 141.89, 141.59, 139.51,

130.92, 129.10, 128.29 (2C), 126.76 (2C), 120.23, 118.08 (d, J = 270.9 Hz), 112.56, 109.52, 108.86, 108.19, 50.84, 24.91 (2C). HRMS (ESI): m/z $[M + H]^+$ calcd for $C_{23}H_{18}F_4N_6O_2S$ 519.1226, found 519.1222. Retention time 2.309 min, HPLC purity = 97.620%.

3-(4-fluoro-1-isopropyl-2-methyl-1H-benzo[d]imidazol-6-yl)-5-(4-((4-methylpiperazin-1-yl)methyl)phenyl)-1H-pyrazolo[3,4-b]pyridine ***(15x)***

Yellow solid. Yield: 42%. ^{1}H NMR (400 MHz, DMSO-d_6) δ 8.93 (s, 1H), 8.72 (s, 1H), 8.23 (s, 1H), 7.98 (d, J = 11.2 Hz, 1H), 7.95 (d, J = 7.8 Hz, 2H), 7.85 (d, J = 6.4 Hz, 2H), 5.07 – 5.01 (m, 1H), 4.54 (s, 2H), 3.64 – 3.56 (m, 8H), 3.48 (s, 3H), 2.87 (s, 3H), 1.67 (d, J = 6.6 Hz, 6H). ^{13}C NMR (126 MHz, DMSO-d_6) δ 153.42, 152.92, 151.68, 149.70 (d, J = 249.5 Hz), 149.05, 142.49, 139.40, 135.03, 132.65, 130.43, 129.68, 129.45 (2C), 128.80 (2C), 128.12, 112.52, 109.48, 107.66, 58.85, 50.14, 48.69 (2C), 46.43 (2C), 42.33, 21.00 (2C), 13.80. HRMS (ESI): m/z $[M + H]^+$ calcd for $C_{29}H_{32}FN_7$ 498.2781, found 498.2781. Retention time 2.121 min, HPLC purity = 98.862%.

*3-(3-(4-fluoro-1-isopropyl-2-methyl-1H-benzo[d]imidazol-6-yl)-1H-pyrazolo[3,4-b]pyridin-5-yl)benzenesulfonamide(****15y****)*

Yellow solid. Yield: 49%. ^{1}H NMR (400 MHz, DMSO-d_6) δ 8.98 (d, J = 2.0 Hz, 1H), 8.81 (d, J = 2.0 Hz, 1H), 8.36 (s, 1H), 8.32 (s, 1H), 8.15 – 8.11 (m, 2H), 7.88 (d, J = 7.8 Hz, 1H), 7.74 (t, J = 7.8 Hz, 1H), 7.49 (s, 2H), 5.13 – 5.09 (m, 1H), 2.94 (s, 3H), 1.71 (d, J = 6.9 Hz, 6H). ^{13}C NMR (126 MHz, DMSO-d_6) δ 153.43, 153.07, 150.97, 149.02 (d, J = 240.7 Hz), 145.51, 142.41, 139.10, 134.41, 131.21 (2C), 130.25, 129.38 (2C), 128.91, 124.94 (2C), 112.61, 110.32, 108.30, 50.71, 20.85 (2C), 13.34. HRMS (ESI): m/z $[M + H]^+$ calcd for $C_{23}H_{21}FN_6O_2S$ 465.1509, found 465.1509. Retention time 2.471 min, HPLC purity = 100%.

*3-(3-(1-cyclopropyl-4-fluoro-2-methyl-1H-benzo[d]imidazol-6-yl)-1H-pyrazolo[3,4-b]pyridin-5-yl)benzenesulfonamide(****15z****)*

Yellow solid. Yield: 50%. ^{1}H NMR (400 MHz, DMSO-d_6) δ 8.97 (s, 1H), 8.86 (s, 1H), 8.33 (s, 1H), 8.24 (s, 1H), 8.14 – 8.10 (m, 2H), 7.88 (d, J = 7.6 Hz, 1H), 7.75 (s, 1H), 7.50 (s, 2H), 3.64 (s, 1H), 2.89 (s, 3H), 1.40 – 1.28 (m, 4H). ^{13}C NMR (126 MHz, DMSO-d_6) δ 156.34, 153.07, 149.01 (d, J = 238.1 Hz), 145.48, 142.21, 139.05, 137.11, 131.95 (2C), 131.18, 130.26, 129.45 (2C), 129.19, 128.78, 124.95, 112.50, 109.89, 107.07, 26.66, 13.55, 6.77 (2C). HRMS (ESI): m/z $[M + H]^+$ calcd for $C_{23}H_{19}N_6O_2S$ 463.1352, found 463.1350. Retention time 2.408 min, HPLC purity = 100%.

*3-(3-(4-fluoro-1-isopropyl-2-(trifluoromethyl)-1H-benzo[d]imidazol-6-yl)-1H-pyrazolo[3,4-b]pyridin-5-yl)benzenesulfonamide(**15aa**)*

Yellow solid. Yield: 46%. ^{1}H NMR (600 MHz, DMSO-d_6) δ 14.18 (s, 1H), 9.03 (d, *J* = 2.0 Hz, 1H), 8.84 (d, *J* = 2.0 Hz, 1H), 8.40 (s, 1H), 8.31 (s, 1H), 8.25 (d, *J* = 7.9 Hz, 1H), 7.96 (dd, *J* = 12.3, 9.9 Hz, 2H), 7.82 (t, *J* = 7.8 Hz, 1H), 7.21 (s, 2H), 5.01 (dt, *J* = 13.7, 6.8 Hz, 1H), 1.74 (d, *J* = 6.9 Hz, 6H). ^{13}C NMR (126 MHz, DMSO-d_6) δ 153.10, 148.89 (d, *J* = 236.9 Hz), 145.45, 142.86, 141.91, 139.14, 132.50, 131.99, 131.27, 130.22, 129.84, 129.40, 129.27, 128.77, 124.88, 120.22, 118.07 (d, *J* = 270.9 Hz), 112.57, 108.26, 50.64, 25.38 (2C). HRMS (ESI): m/z $[M + H]^+$ calcd for $C_{23}H_{18}F_4N_6O_2S$ 519.1226, found 519.1224. Retention time 2.302 min, HPLC purity = 98.256%.

*4-(3-(4-fluoro-1-isopropyl-2-(trifluoromethyl)-1H-benzo[d]imidazol-6-yl)-1H-pyrrolo[2,3-b]pyridin-5-yl)benzenesulfonamide(**15ab**)*

Yellow solid. Yield: 50%. ^{1}H NMR (400 MHz, DMSO-d_6) δ 12.14 (s, 1H), 8.66 (d, *J* = 1.9 Hz, 1H), 8.49 (d, *J* = 1.7 Hz, 1H), 8.03 (s, 1H), 8.00 (d, *J* = 8.4 Hz, 2H), 7.93 (d, *J* = 8.4 Hz, 2H), 7.75 (s, 1H), 7.42 (s, 2H), 7.38 (s, 1H), 4.86-4.80 (m, 1H), 2.61 (s, 3H), 1.63 (d, *J* = 6.9 Hz, 6H). ^{13}C NMR (126 MHz, DMSO-d_6) δ 153.41, 153.12, 151.35, 149.38 (d, *J* = 248.2 Hz), 149.18, 143.57, 142.56, 141.66, 134.77, 130.85, 129.24, 129.10 (2C), 128.31 (2C), 126.80, 125.23, 112.54, 109.94, 107.95, 50.41, 20.90 (2C), 13.57. HRMS (ESI): m/z $[M + H]^+$ calcd for $C_{24}H_{22}FN_5O_2S$ 464.1556, found 464.1552. Retention time 2.411 min, HPLC purity = 99.686%.

^{1}H NMR spectrum of **26**

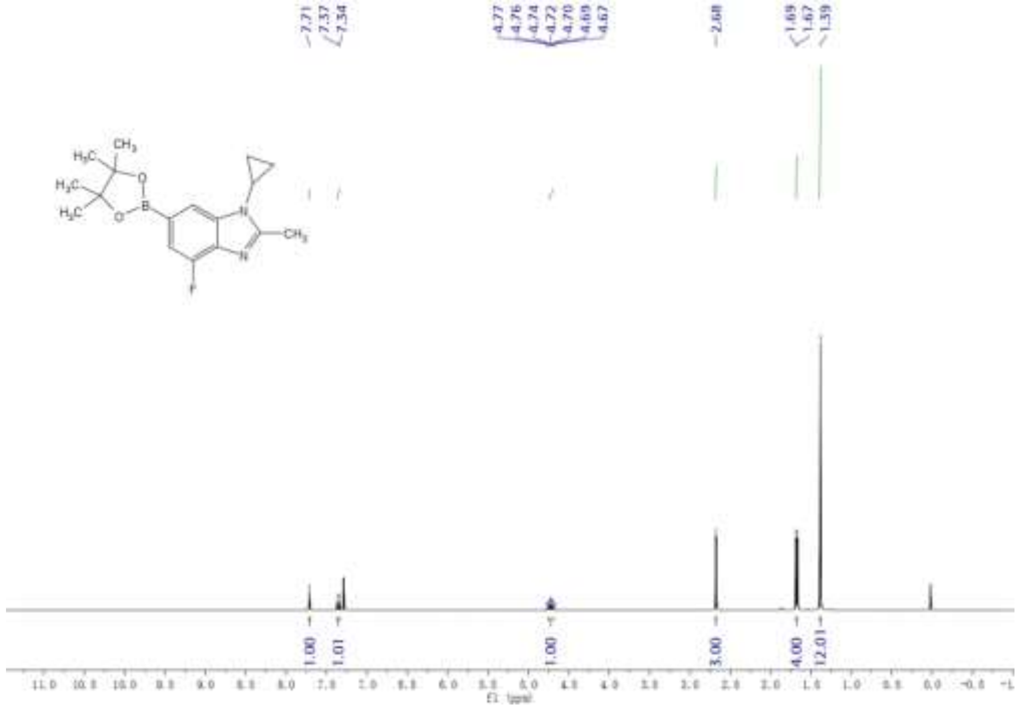

^{1}H NMR spectrum of **30**

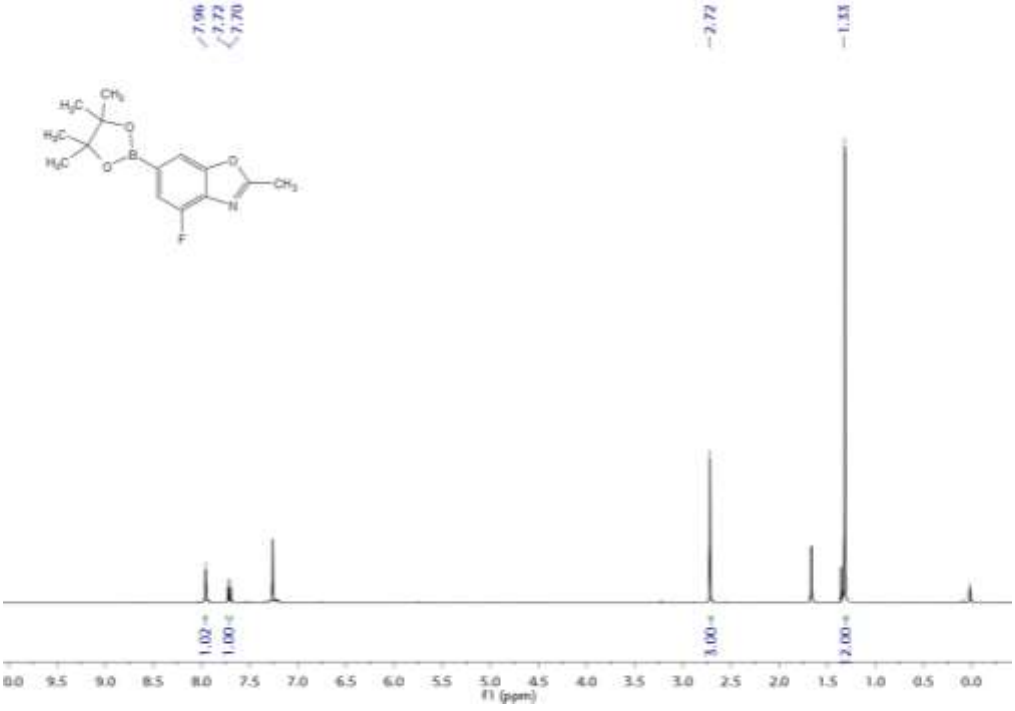

^{1}H NMR spectrum of **34**

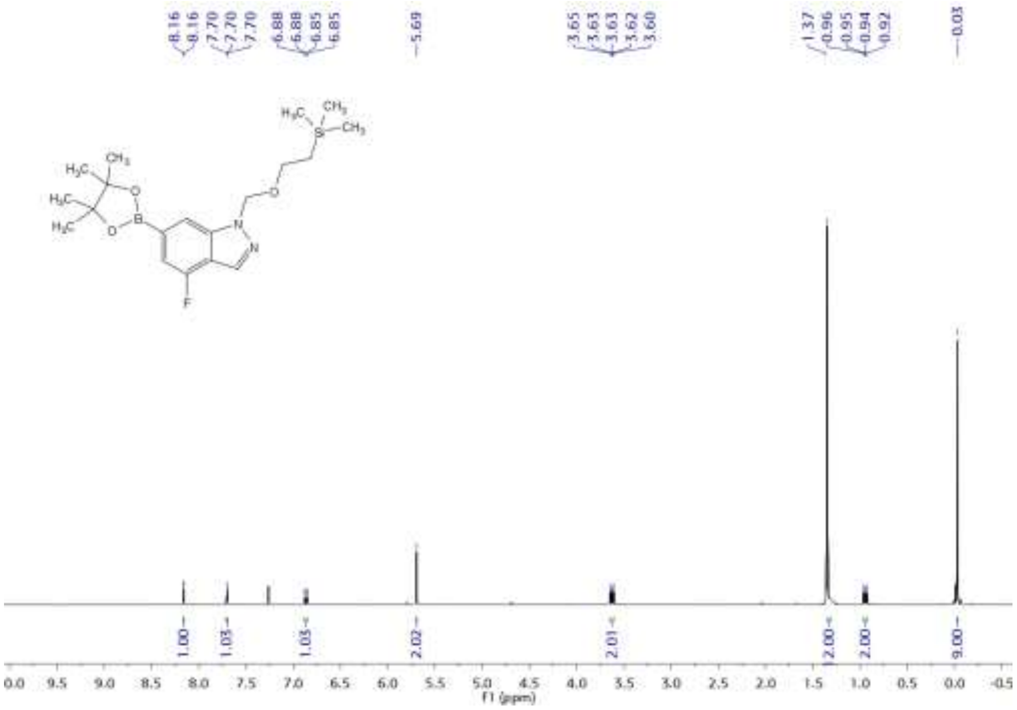

^{1}H NMR spectrum of **38**

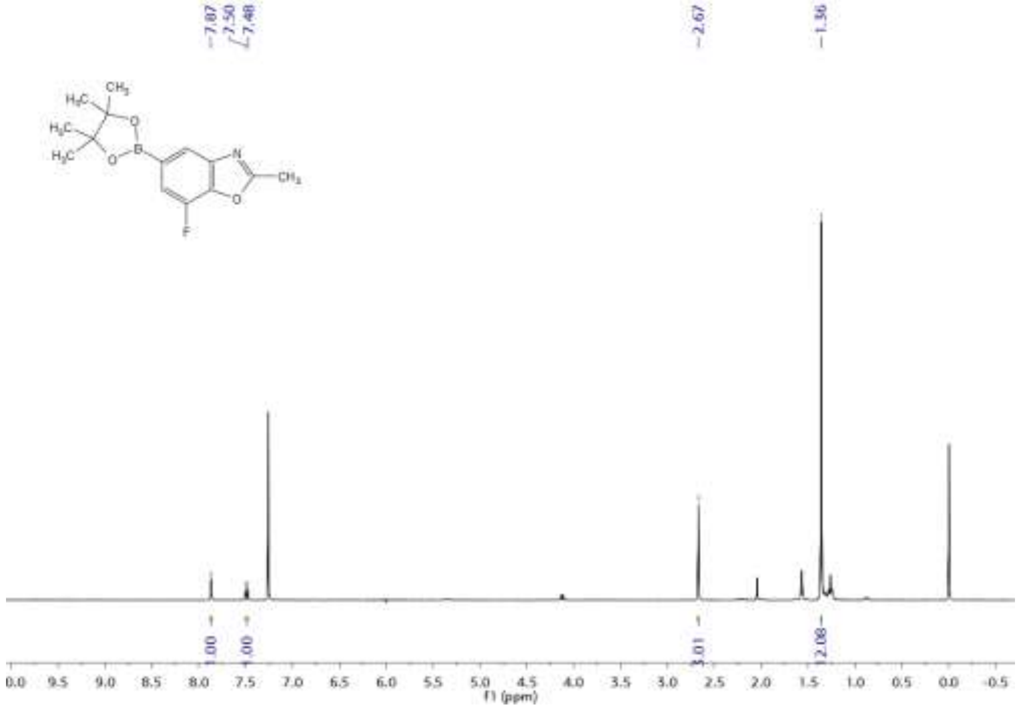

^{1}H NMR spectrum of **43**

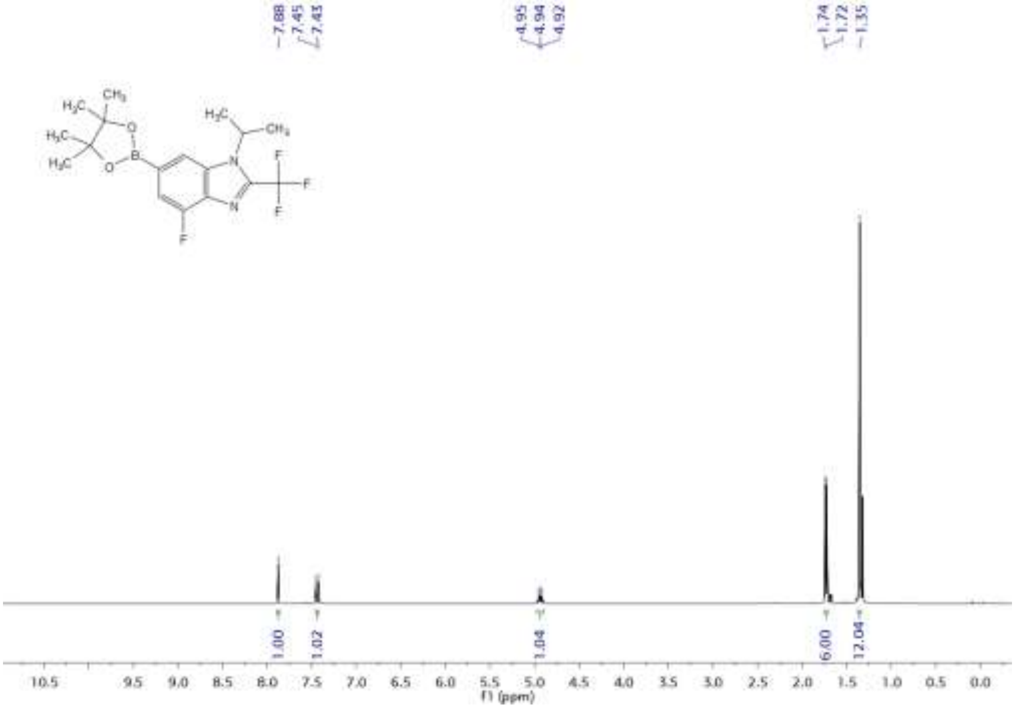

^{1}H NMR, ^{13}C NMR, HRMS and HPLC spectra of **12a**

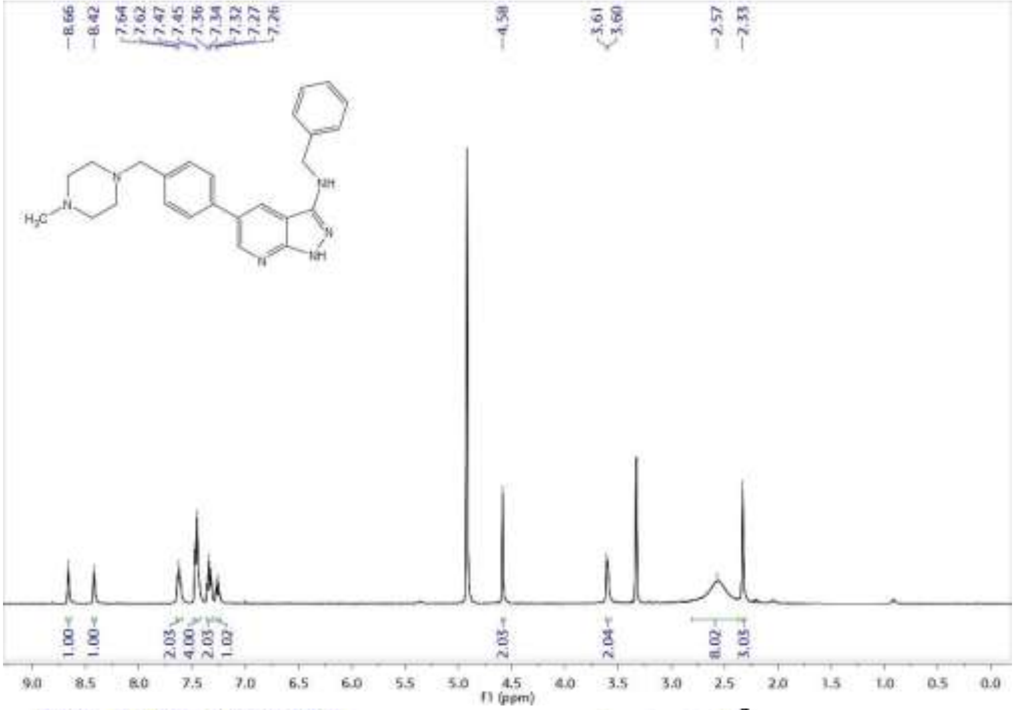

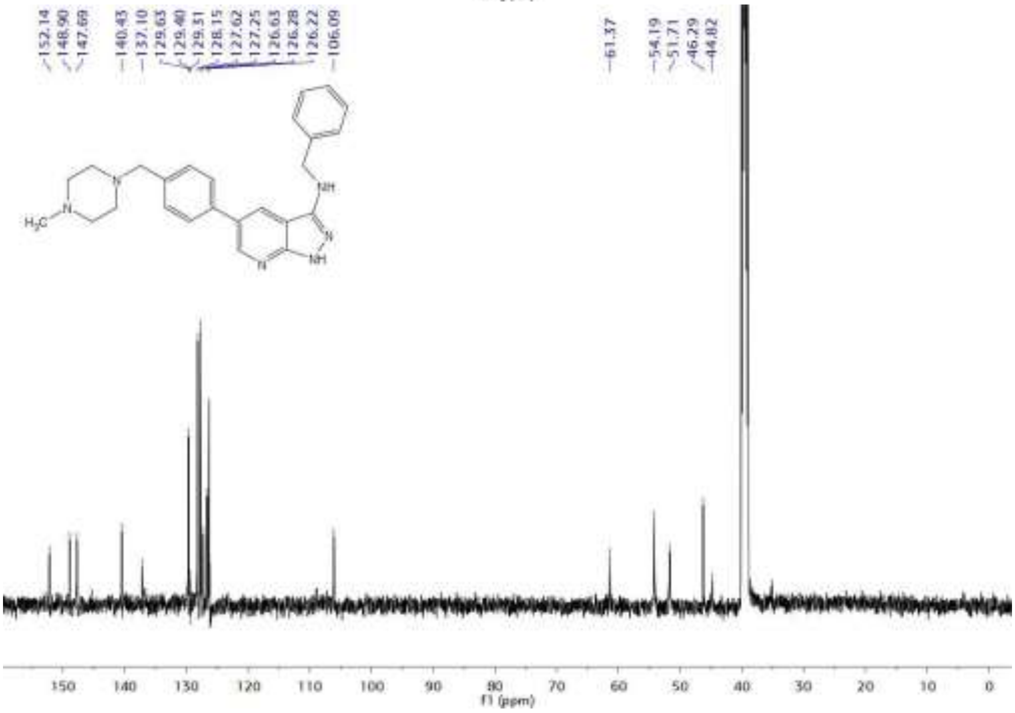

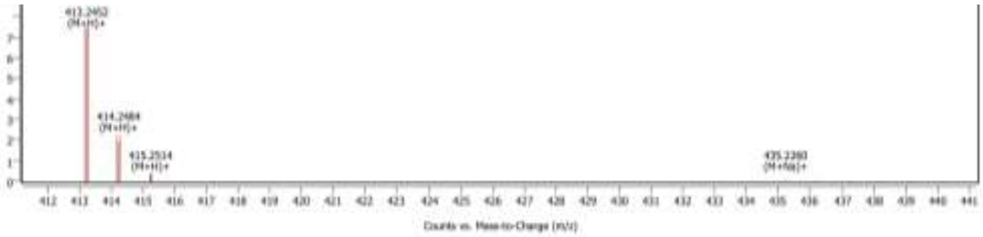

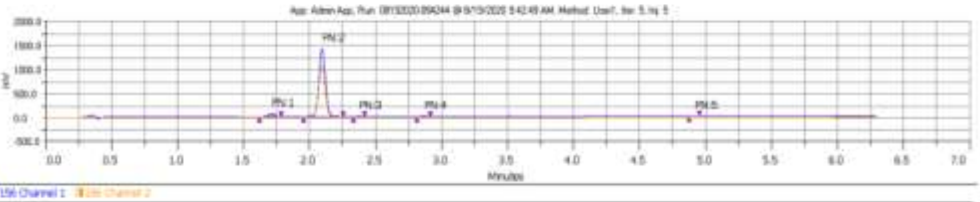

Sample Table

Injection Number	Sample Name	Sample Location	Peak Name	Retention Time (min)	Area (µV·min x1000)	Area %	Height (mV)	Plate Number	
5	[illegible]	Sample Zone->4	1	1.713	[illegible]	[illegible]	[illegible]	[illegible]	
5	[illegible]	Sample Zone->4	2	2.097	[illegible]	[illegible]	[illegible]	[illegible]	
5	[illegible]	Sample Zone->4	3	2.375	[illegible]	[illegible]	[illegible]	[illegible]	
5	[illegible]	Sample Zone->4	4	[illegible]	[illegible]	[illegible]	[illegible]	[illegible]	
5	[illegible]	Sample Zone->4	5	[illegible]	[illegible]	[illegible]	[illegible]	[illegible]	

^{1}H NMR, ^{13}C NMR, HRMS and HPLC spectra of **12b**

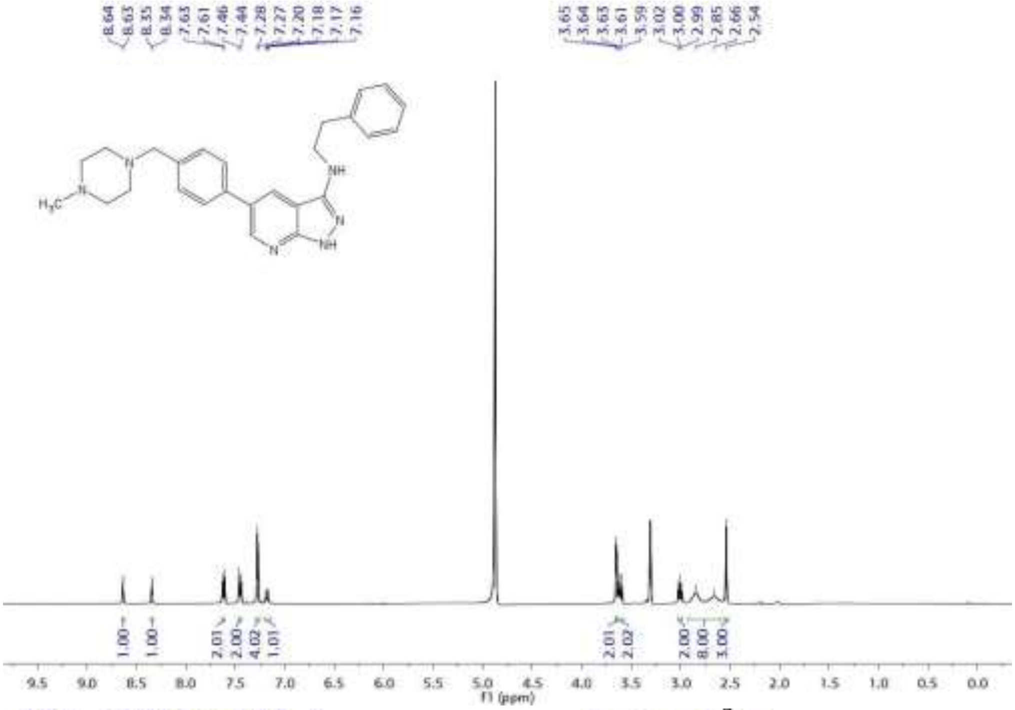

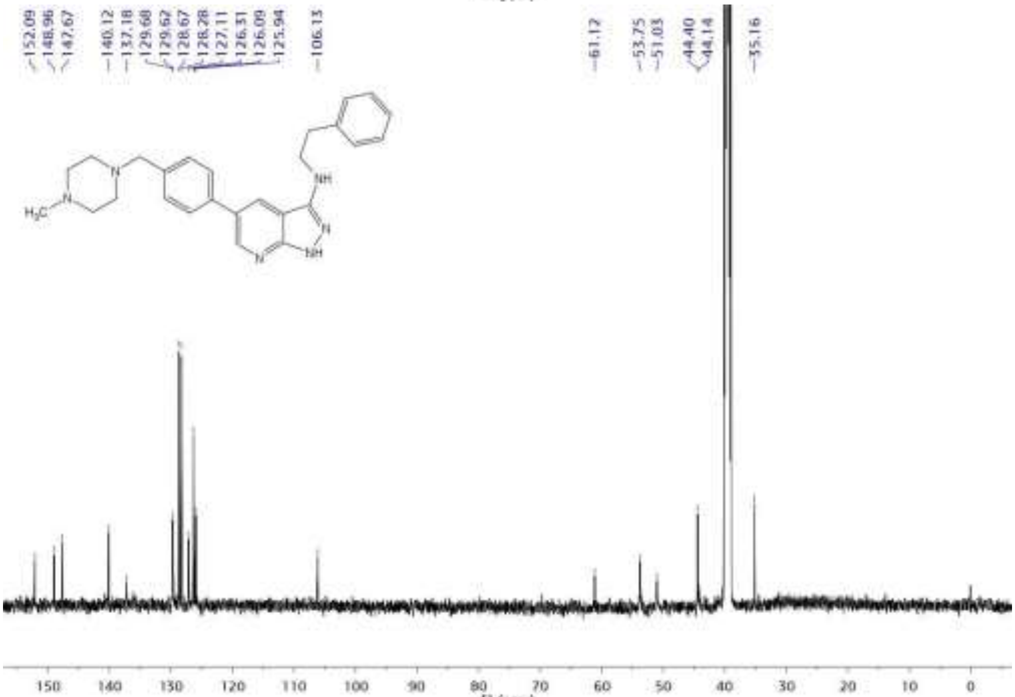

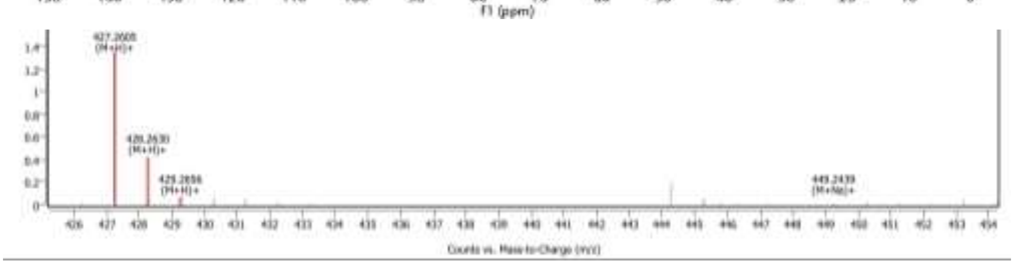

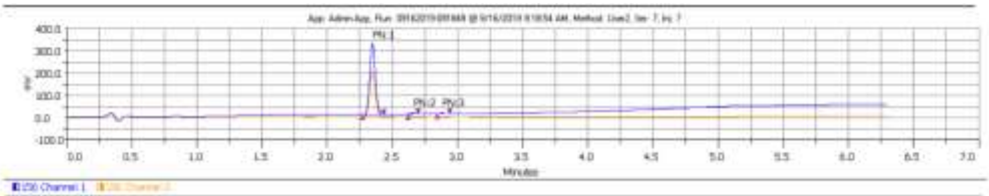

Sample Table

Injection Number	Sample Name	Sample Location	Peak Name	Retention Time (min)	Area [illegible]	Area %	Height (mV)	Plate Number
7	[illegible]	[illegible]	1	[illegible]	[illegible]	[illegible]	[illegible]	[illegible]
7	[illegible]	[illegible]	2	[illegible]	[illegible]	[illegible]	[illegible]	[illegible]
7	[illegible]	[illegible]	3	[illegible]	[illegible]	[illegible]	[illegible]	[illegible]

^{1}H NMR, ^{13}C NMR, HRMS and HPLC spectra of **12c**

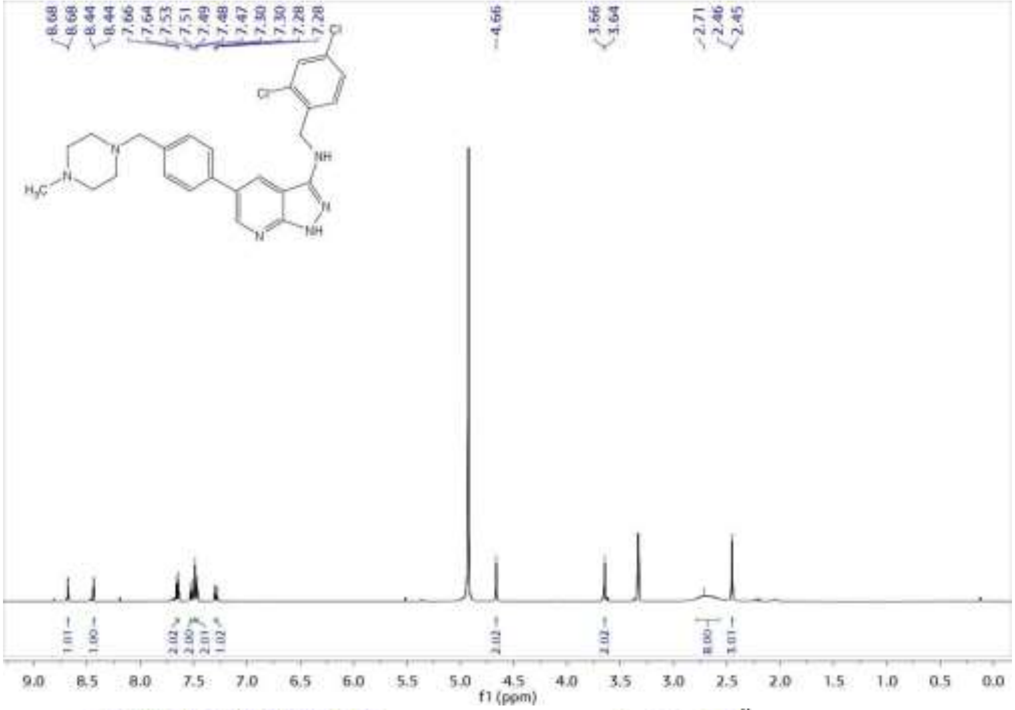

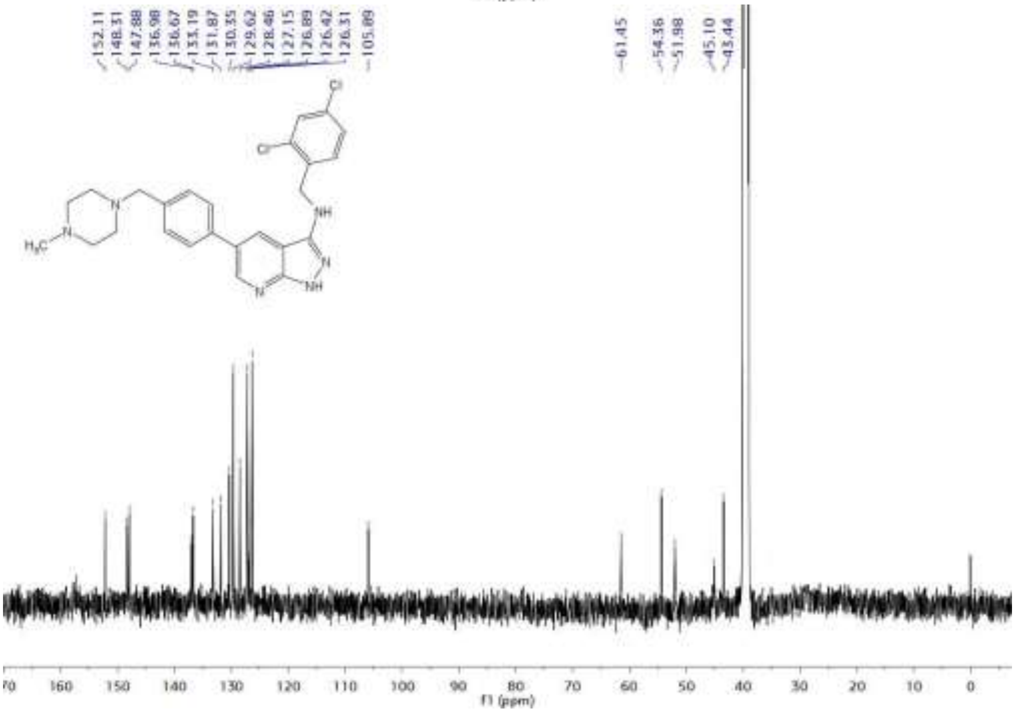

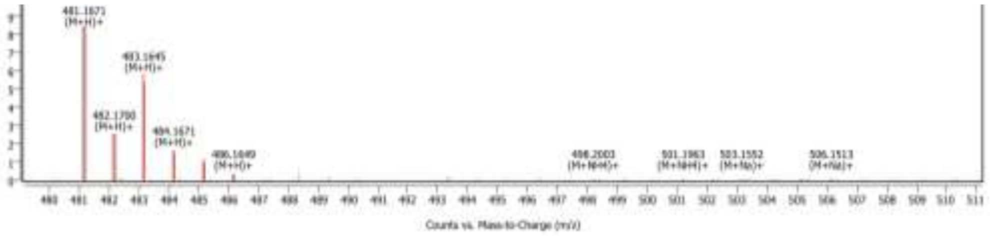

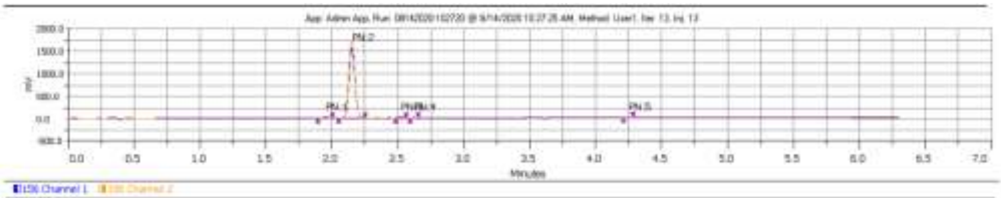

Sample Table

Injection Number	Sample Name	Sample Location	Peak Name	Retention Time (min)	Area [illegible]	Area %	Height [illegible]	Plate Number
[illegible]	[illegible]	[illegible]	1	[illegible]	[illegible]	[illegible]	[illegible]	[illegible]
[illegible]	[illegible]	[illegible]	2	2.154	[illegible]	[illegible]	[illegible]	[illegible]
[illegible]	[illegible]	[illegible]	3	[illegible]	[illegible]	[illegible]	[illegible]	[illegible]
[illegible]	[illegible]	[illegible]	4	[illegible]	[illegible]	[illegible]	[illegible]	[illegible]
[illegible]	[illegible]	[illegible]	5	4.254	[illegible]	[illegible]	[illegible]	[illegible]

^{1}H NMR, ^{13}C NMR, HRMS and HPLC spectra of **12d**

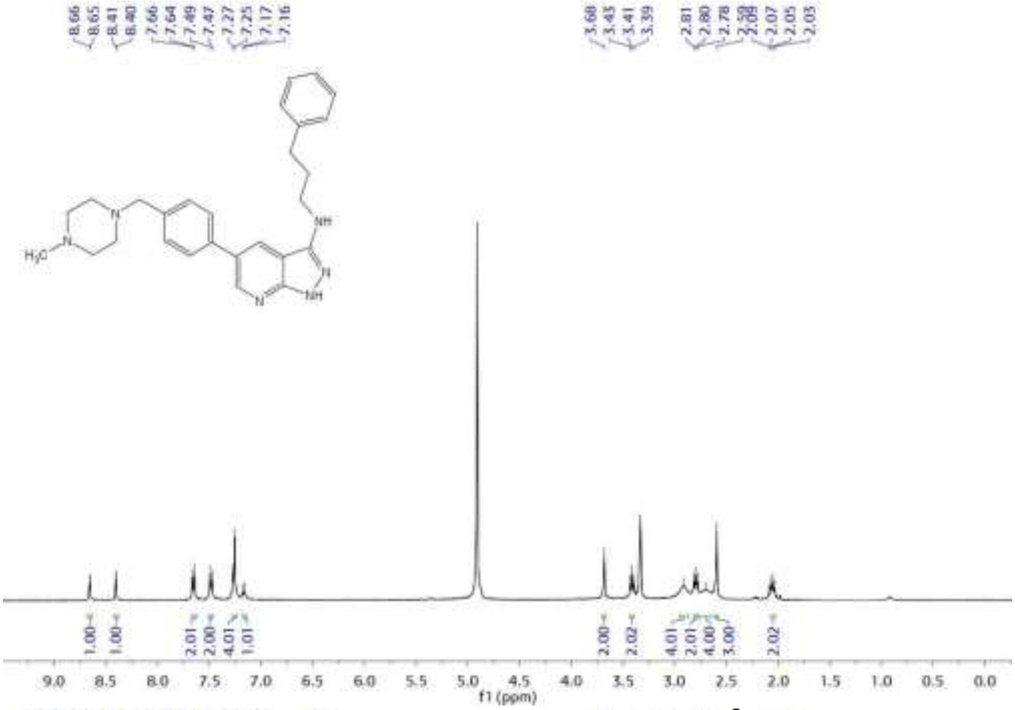

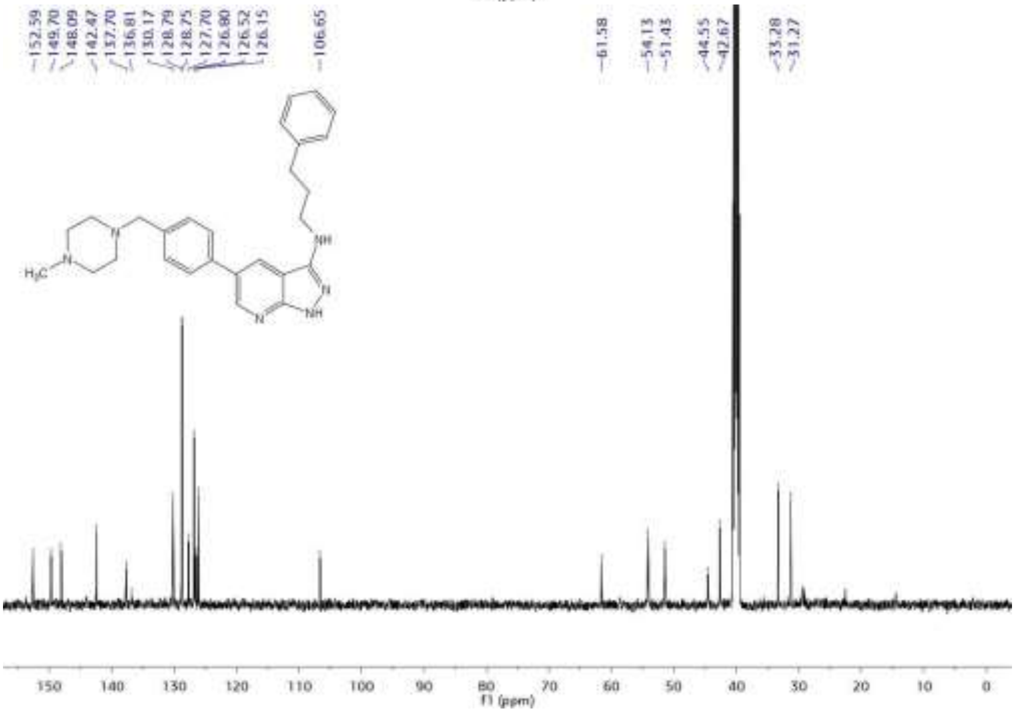

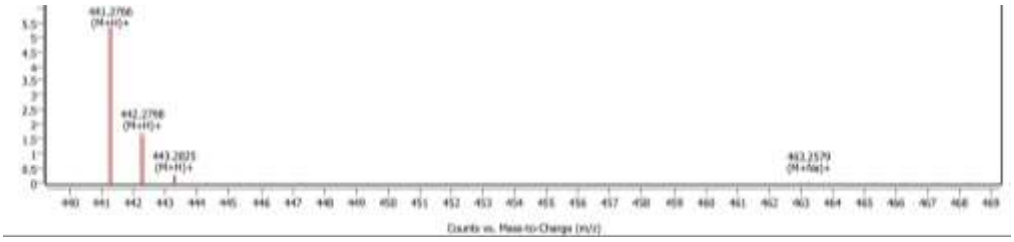

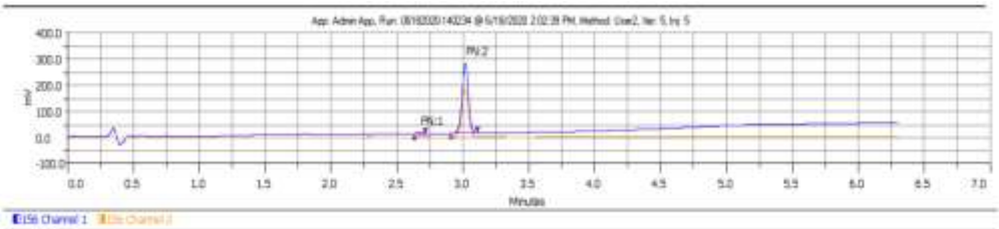

Sample Table

Injection Number	Sample Name	Sample Location	Peak Name	Retention Time (min)	Area (uV*min x1000)	Area %	Height (mV)	Plate Number
1	[illegible]	[illegible]	1	[illegible]	[illegible]	1.12	[illegible]	[illegible]
1	[illegible]	[illegible]	2	[illegible]	[illegible]	[illegible]	[illegible]	[illegible]

^{1}H NMR, ^{13}C NMR, HRMS and HPLC spectra of **12e**

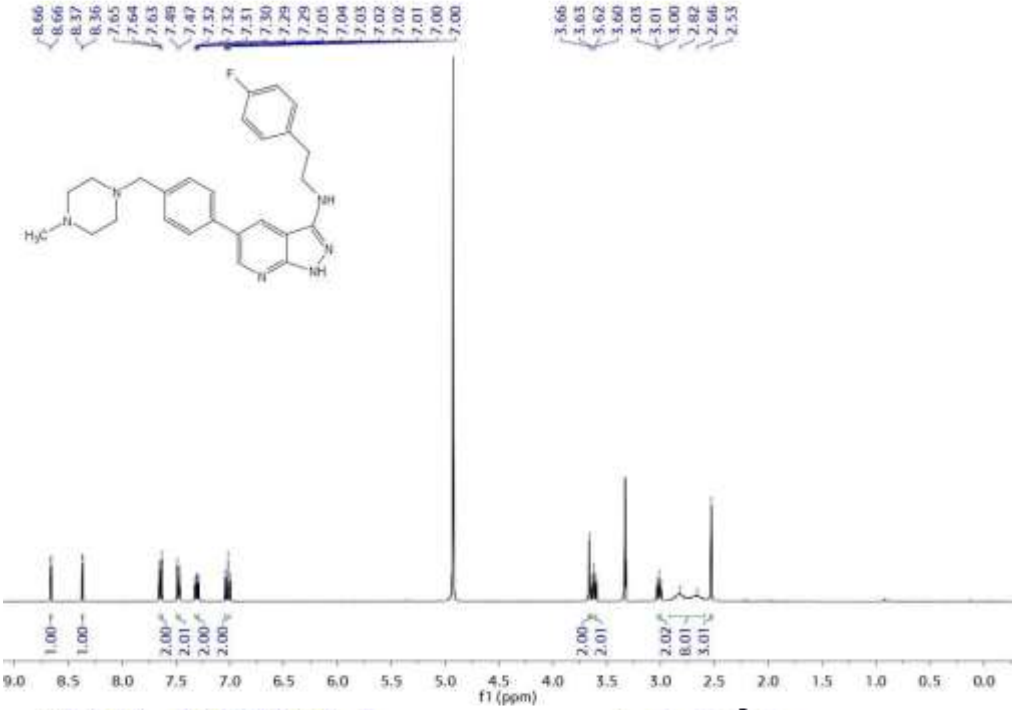

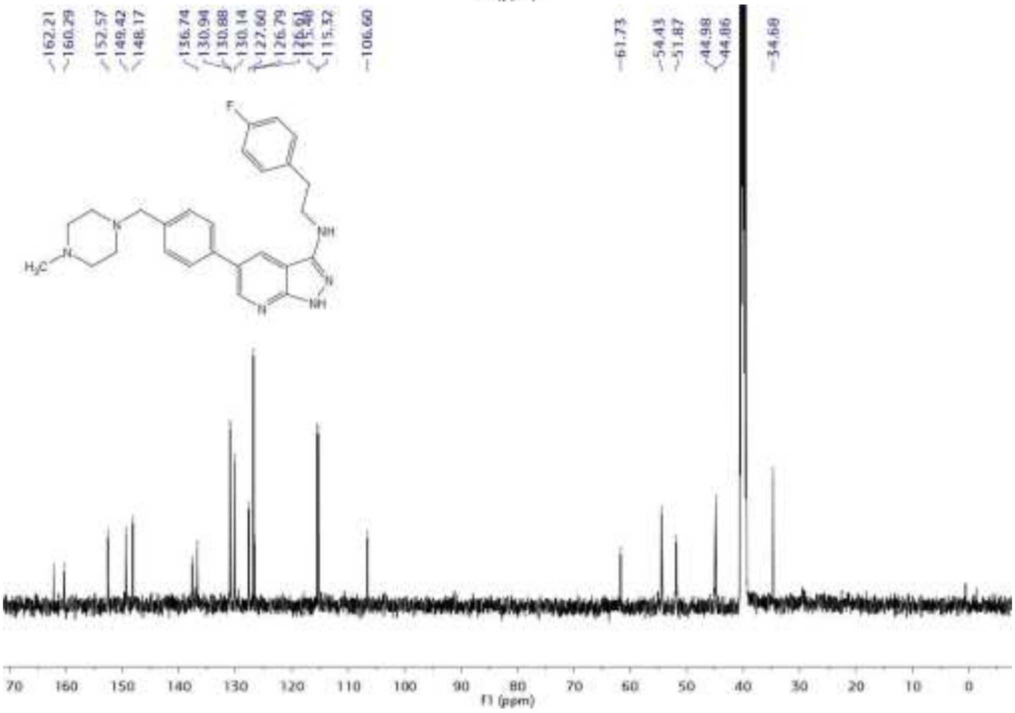

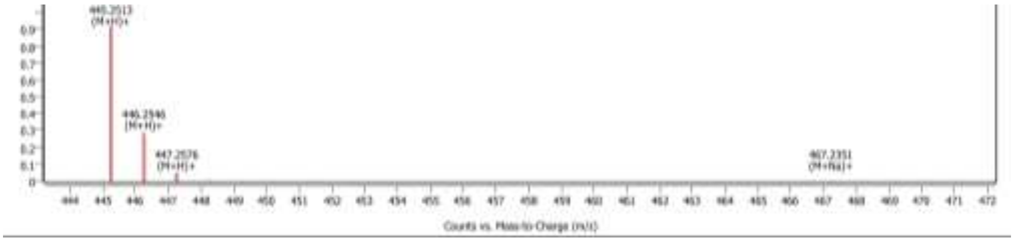

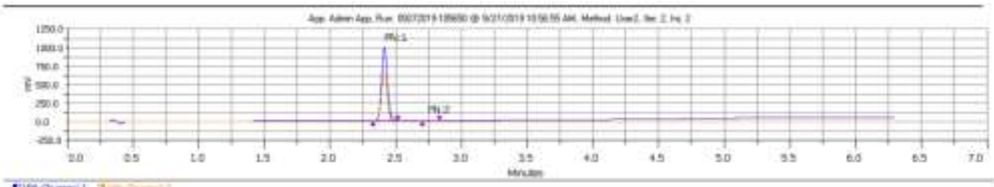

Sample Table

Injection Number	Sample Name	Sample Location	Peak Name	Retention Time (min)	Area (uV*min x100)	Area %	Height (mV)	Plate Number	
[illegible]	[illegible]	[illegible]	1	[illegible]	[illegible]	[illegible]	[illegible]	[illegible]	
[illegible]	[illegible]	[illegible]	2	[illegible]	[illegible]	[illegible]	[illegible]	[illegible]	

^{1}H NMR, ^{13}C NMR, HRMS and HPLC spectra of **12f**

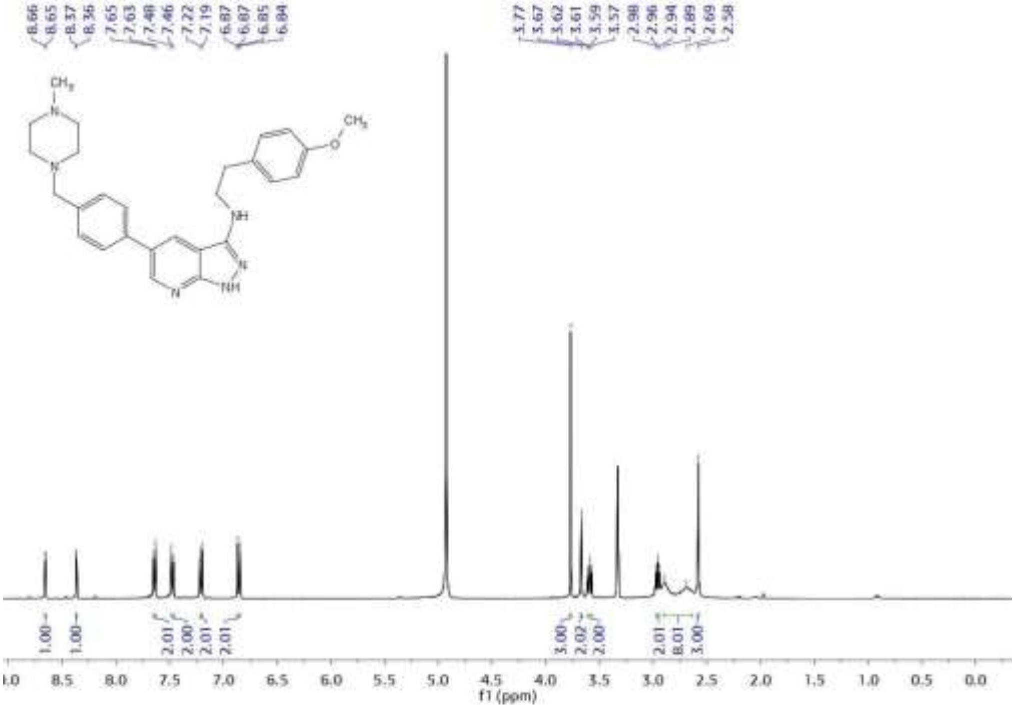

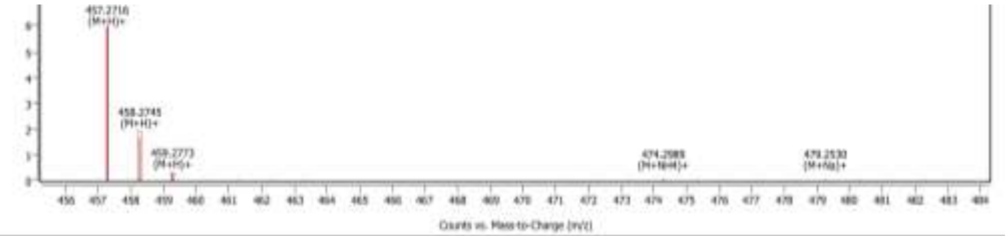

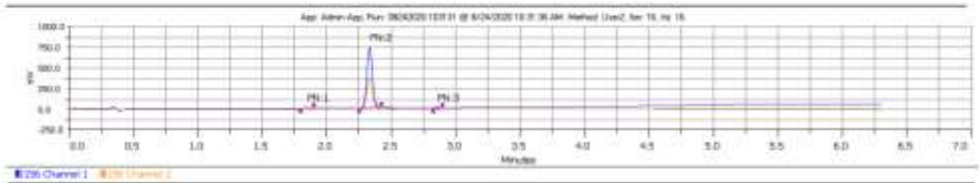

Sample Table

Injection Number	Sample Name	Sample Location	Peak Name	Retention Time (min)	Area [illegible]	Area %	Height (mV)	Plate Number
[illegible]	[illegible]	[illegible]	1	[illegible]	[illegible]	[illegible]	[illegible]	[illegible]
[illegible]	[illegible]	[illegible]	2	[illegible]	[illegible]	[illegible]	[illegible]	[illegible]
[illegible]	[illegible]	[illegible]	3	[illegible]	[illegible]	[illegible]	[illegible]	[illegible]

^{1}H NMR, ^{13}C NMR, HRMS and HPLC spectra of **12g**

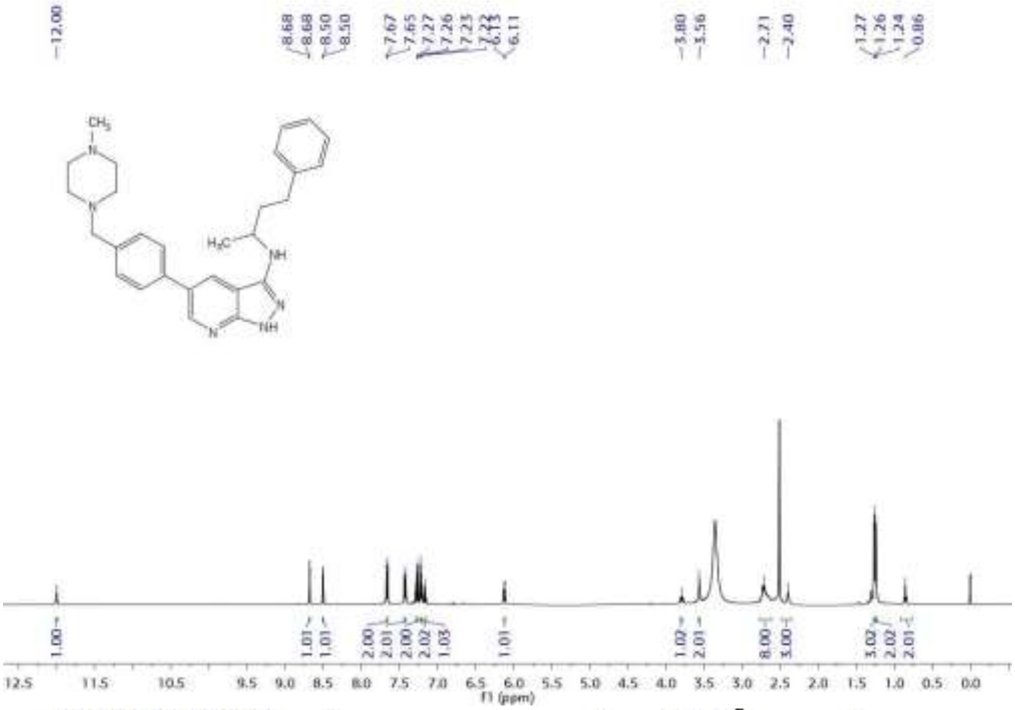

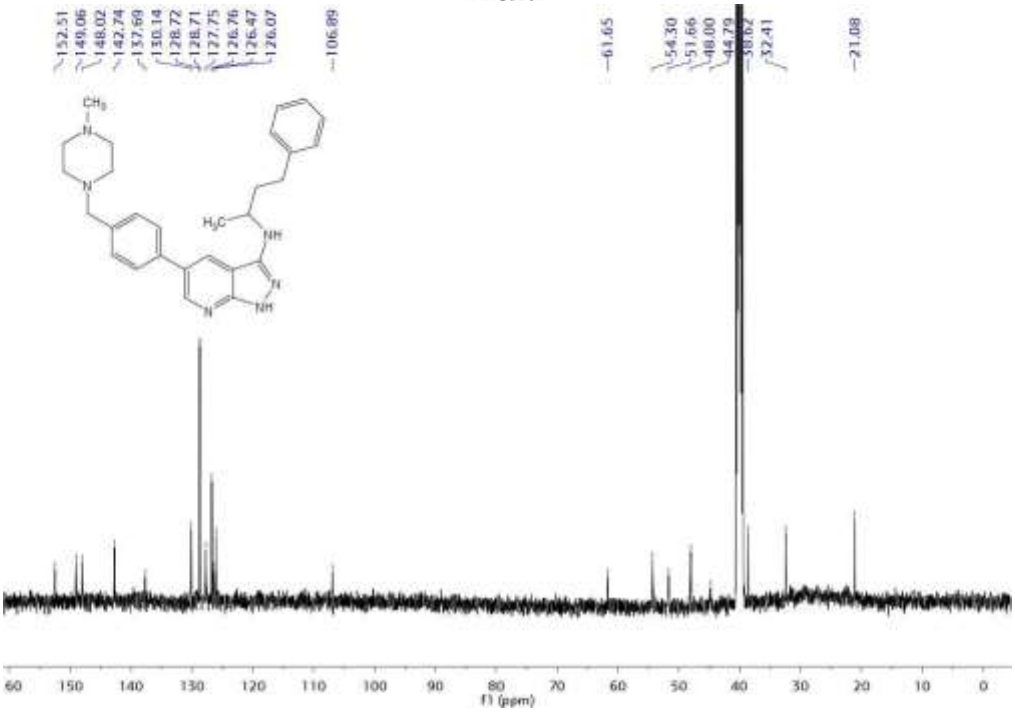

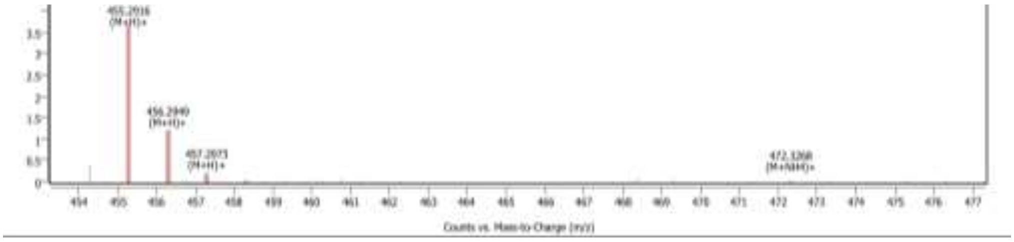

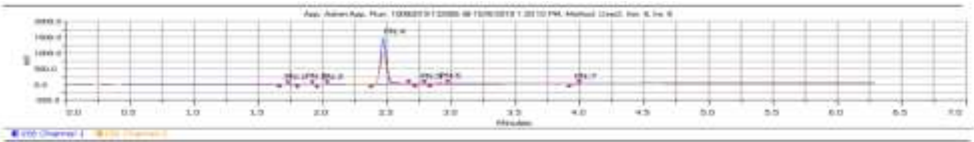

Sample Table

[illegible]	[illegible]	[illegible]	[illegible]	[illegible]	[illegible]	[illegible]	[illegible]	[illegible]
[illegible]	[illegible]	[illegible]	[illegible]	[illegible]	[illegible]	[illegible]	[illegible]	[illegible]
[illegible]	[illegible]	[illegible]	2	[illegible]	[illegible]	[illegible]	[illegible]	[illegible]
[illegible]	[illegible]	[illegible]	3	[illegible]	[illegible]	[illegible]	[illegible]	[illegible]
[illegible]	[illegible]	[illegible]	[illegible]	[illegible]	[illegible]	[illegible]	[illegible]	[illegible]
[illegible]	[illegible]	[illegible]	5	[illegible]	[illegible]	[illegible]	[illegible]	[illegible]
[illegible]	[illegible]	[illegible]	6	[illegible]	[illegible]	[illegible]	[illegible]	[illegible]
[illegible]	[illegible]	[illegible]	7	[illegible]	[illegible]	[illegible]	[illegible]	[illegible]

^{1}H NMR, ^{13}C NMR, HRMS and HPLC spectra of **12h**

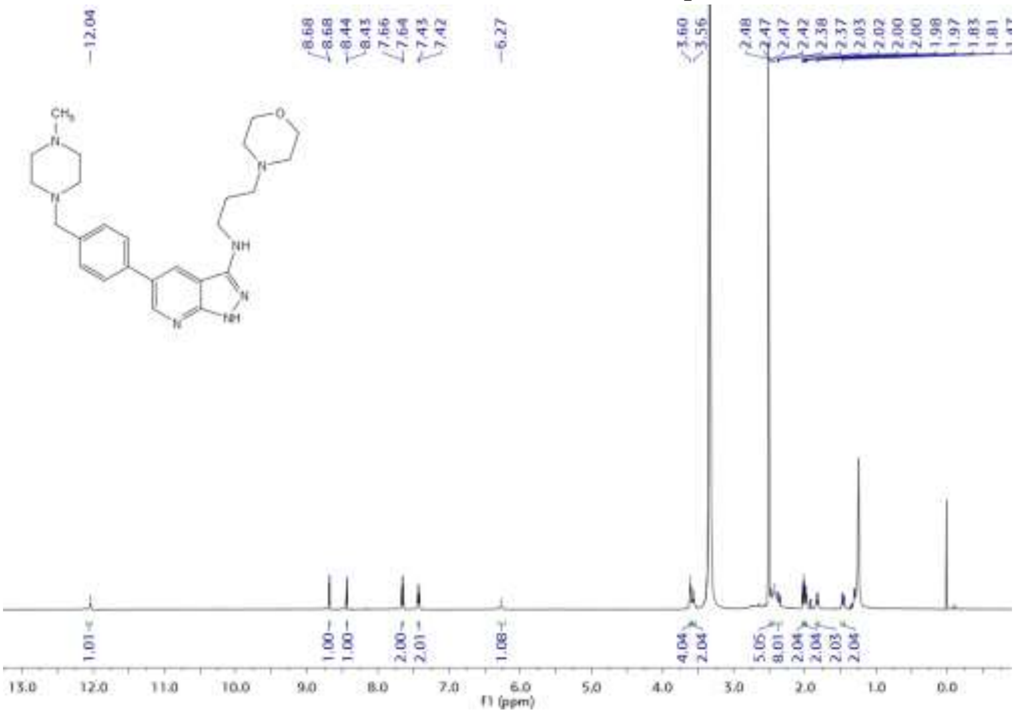

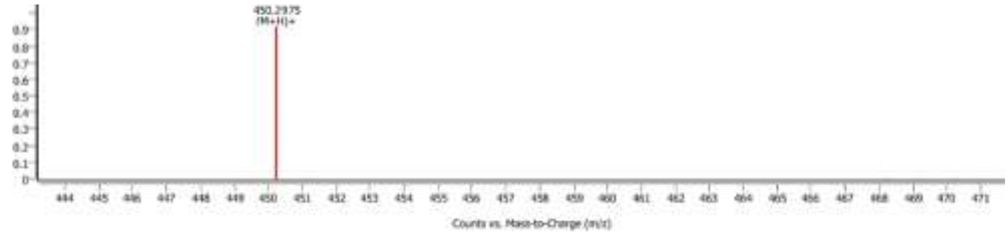

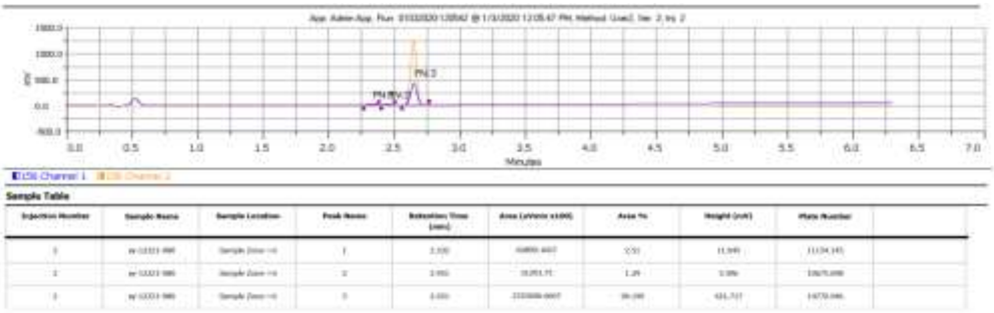

^{1}H NMR, ^{13}C NMR, HRMS and HPLC spectra of **12i**

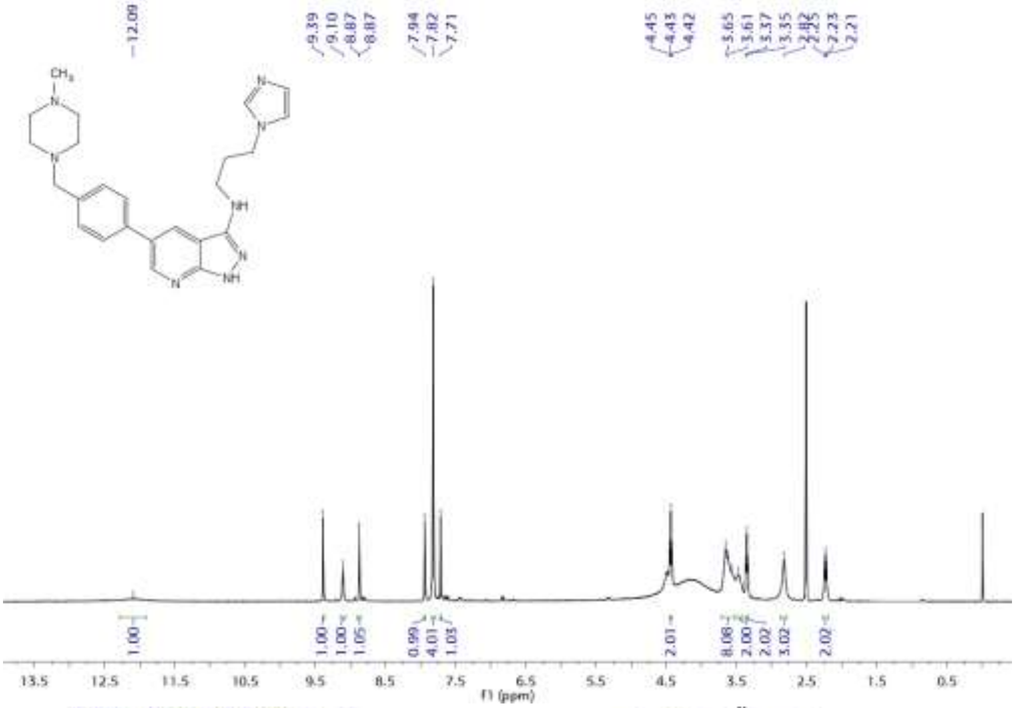

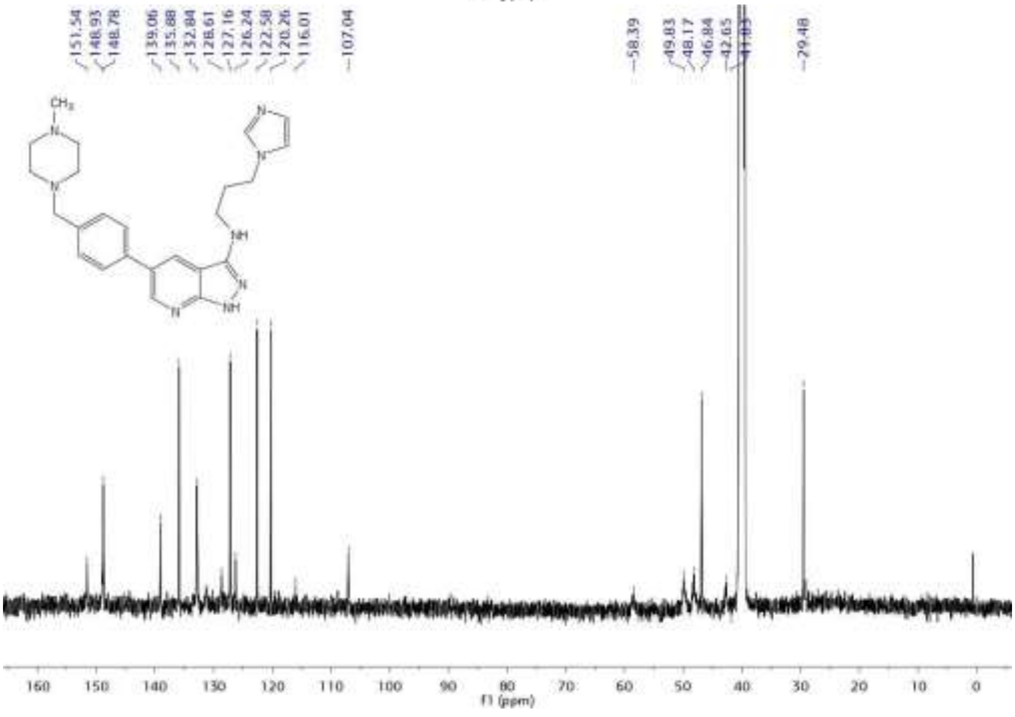

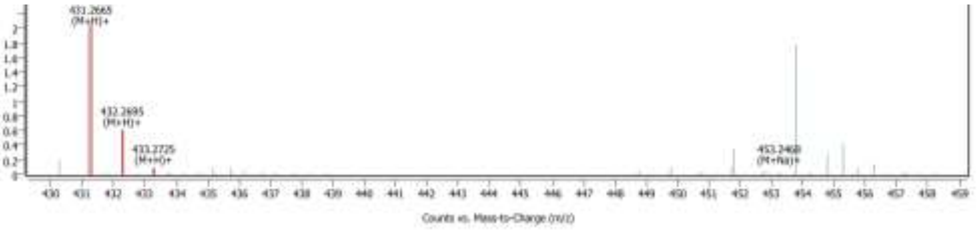

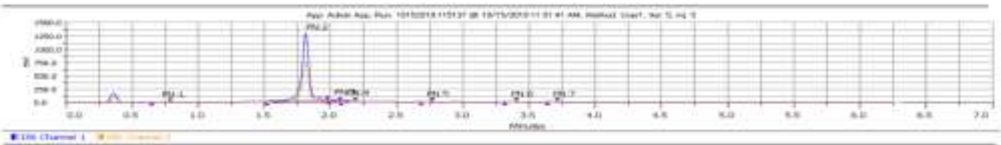

Sample Table

[illegible]	[illegible]	[illegible]	[illegible]	[illegible] (min)	[illegible]	Area %	[illegible]	[illegible]
[illegible]	[illegible]	[illegible]	1	[illegible]	[illegible]	[illegible]	[illegible]	[illegible]
[illegible]	[illegible]	[illegible]	2	[illegible]	[illegible]	[illegible]	[illegible]	[illegible]
[illegible]	[illegible]	[illegible]	3	[illegible]	[illegible]	[illegible]	[illegible]	[illegible]
[illegible]	[illegible]	[illegible]	4	[illegible]	[illegible]	[illegible]	[illegible]	[illegible]
[illegible]	[illegible]	[illegible]	5	[illegible]	[illegible]	[illegible]	[illegible]	[illegible]
[illegible]	[illegible]	[illegible]	6	[illegible]	[illegible]	[illegible]	[illegible]	[illegible]
[illegible]	[illegible]	[illegible]	7	[illegible]	[illegible]	[illegible]	[illegible]	[illegible]

^{1}H NMR, ^{13}C NMR, HRMS and HPLC spectra of **15b**

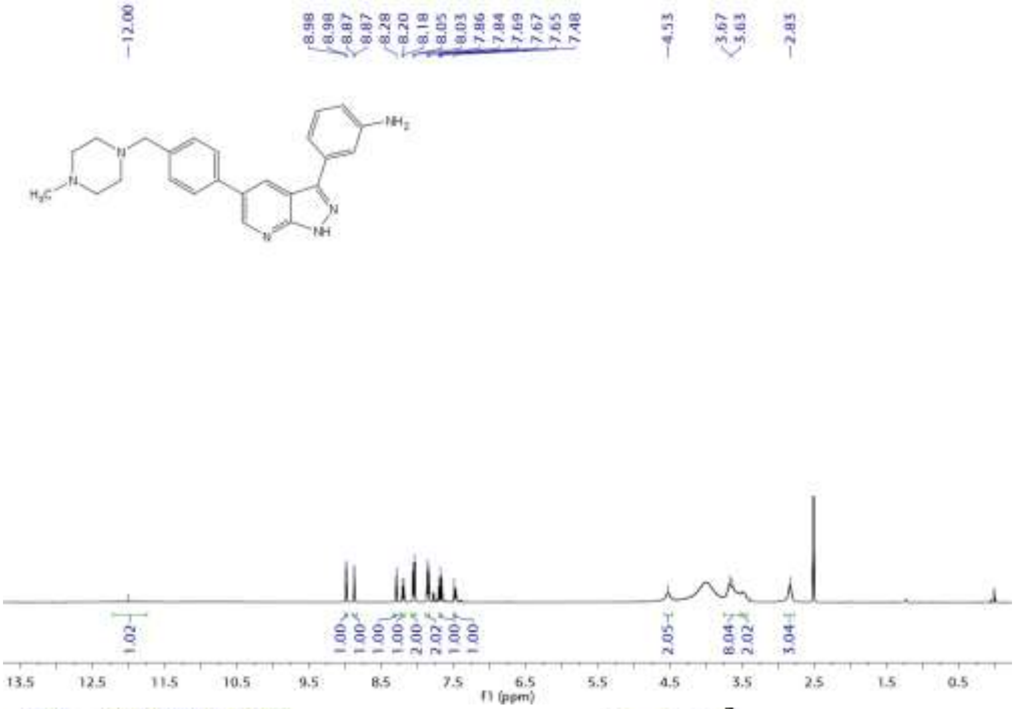

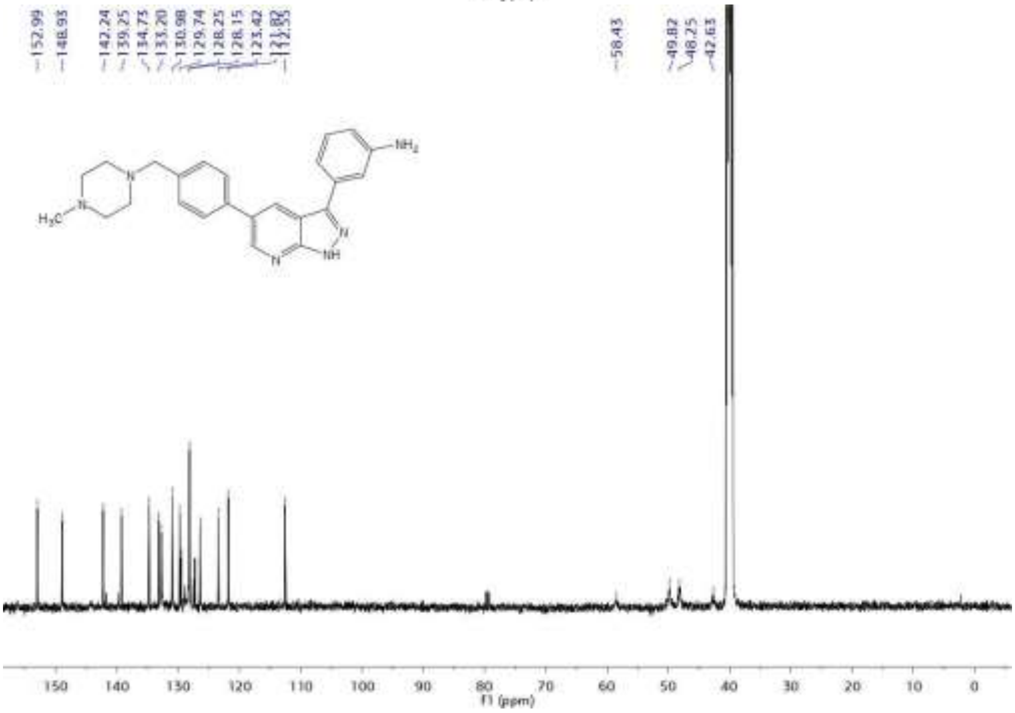

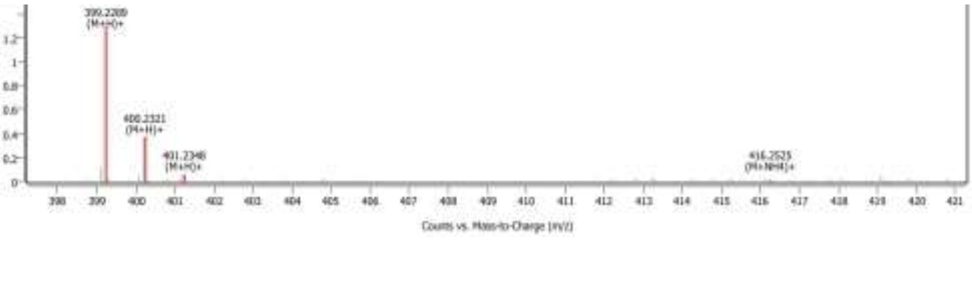

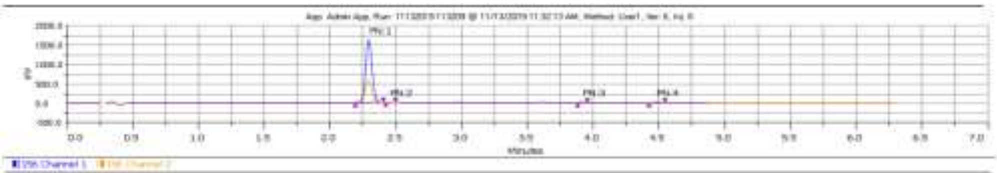

Sample Table

Injection Number	Sample Name	Sample Location	Peak Name	Retention Time (min)	Area (uV*sec x1000)	Area %	Height (mV)	Plate Number
[illegible]	[illegible]	[illegible]	1	[illegible]	[illegible]	[illegible]	[illegible]	[illegible]
[illegible]	[illegible]	[illegible]	2	[illegible]	[illegible]	[illegible]	[illegible]	[illegible]
[illegible]	[illegible]	[illegible]	3	[illegible]	[illegible]	[illegible]	[illegible]	[illegible]
[illegible]	[illegible]	[illegible]	4	[illegible]	[illegible]	[illegible]	[illegible]	[illegible]

^{1}H NMR, ^{13}C NMR, HRMS and HPLC spectra of **15c**

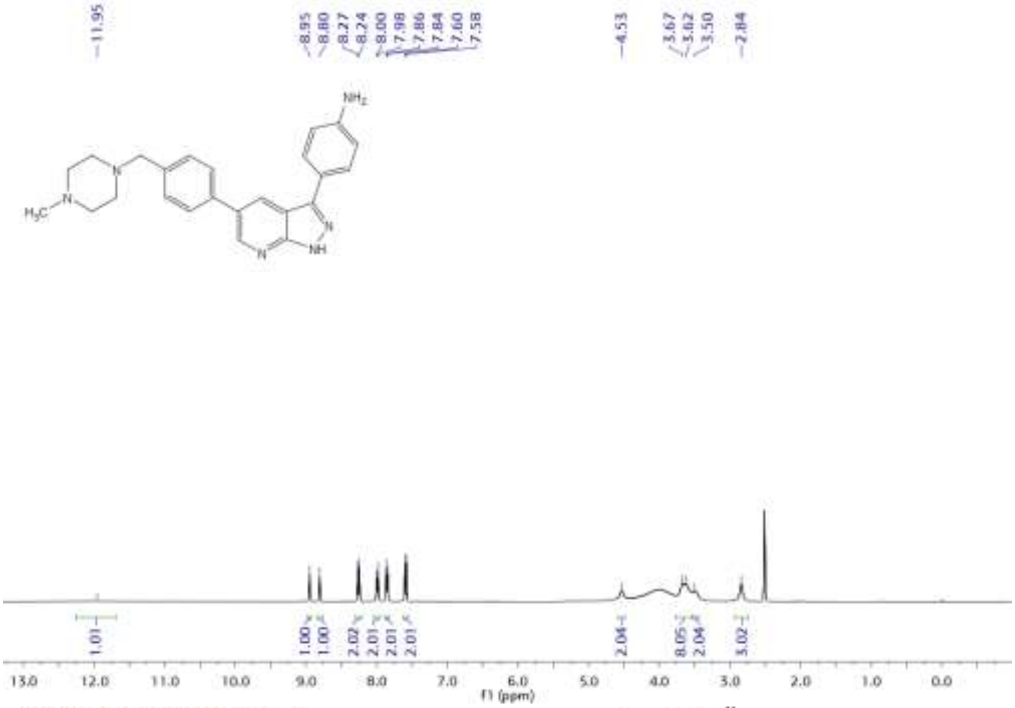

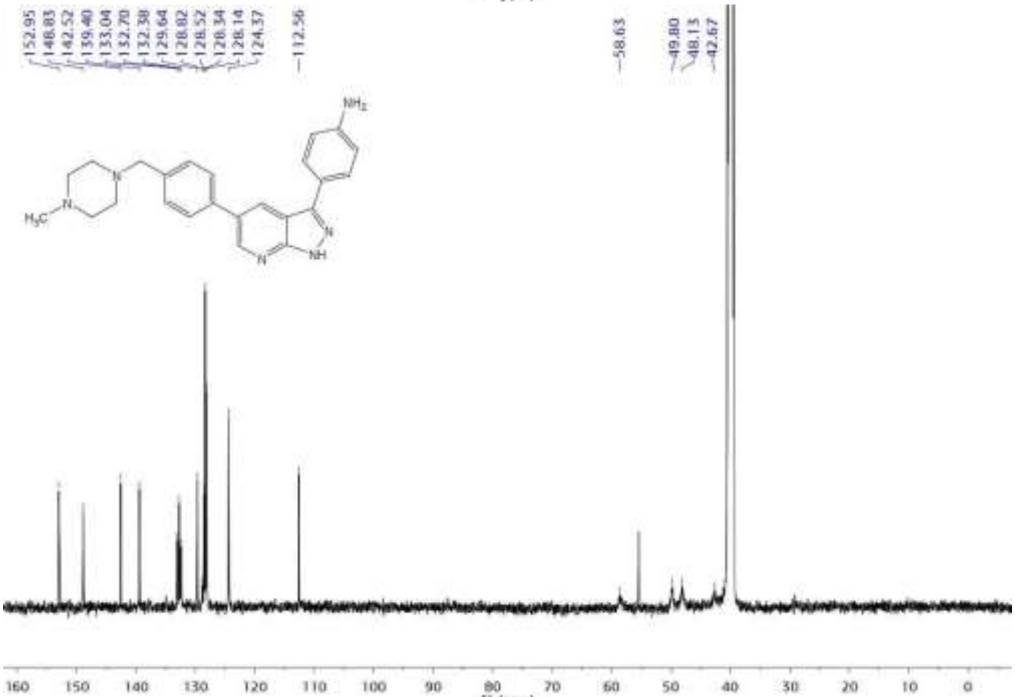

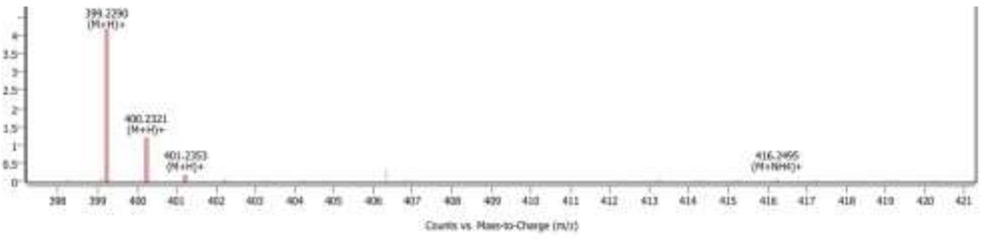

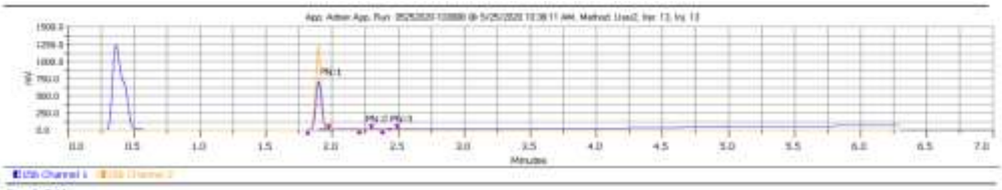

Sample Table

Injection Number	Sample Name	Sample Location	Peak Name	Retention Time (min)	Area ([illegible])	Area %	Height (mV)	Plate Number
13	[illegible]	[illegible]	1	[illegible]	[illegible]	[illegible]	[illegible]	[illegible]
13	[illegible]	[illegible]	2	[illegible]	[illegible]	[illegible]	[illegible]	[illegible]
13	[illegible]	[illegible]	3	[illegible]	[illegible]	[illegible]	[illegible]	[illegible]

^{1}H NMR, ^{13}C NMR, HRMS and HPLC spectra of **15a**

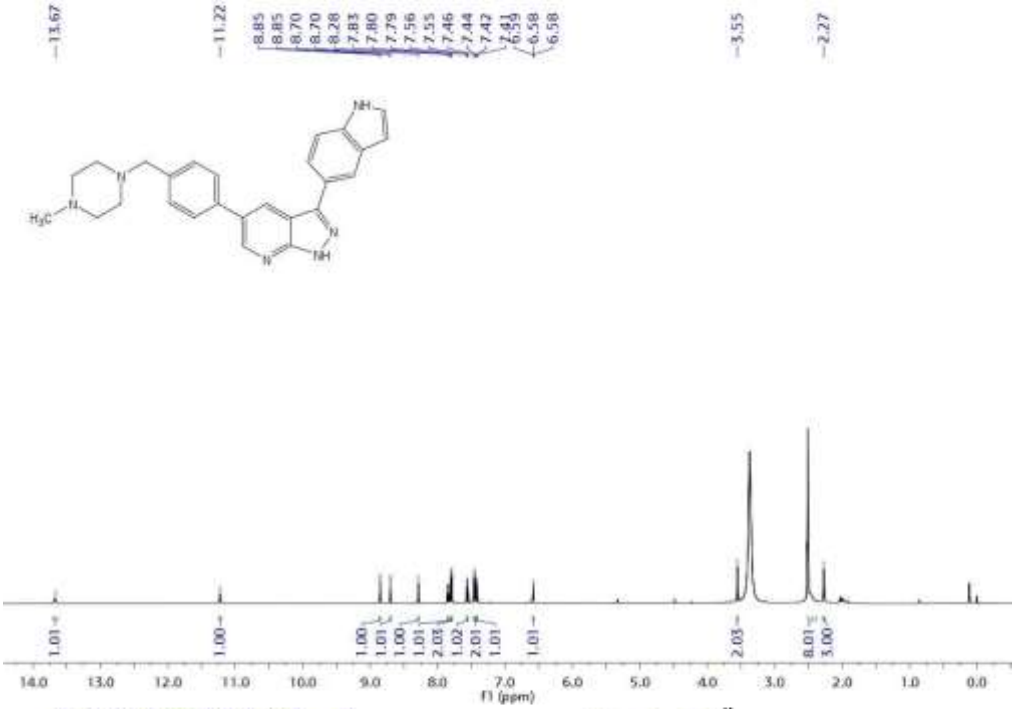

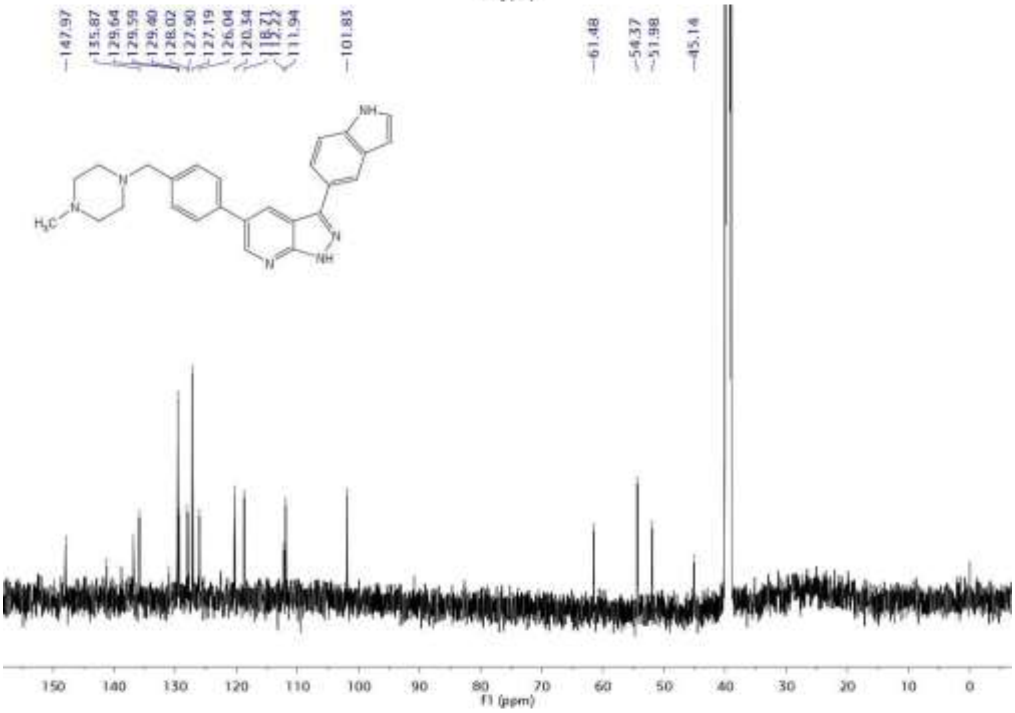

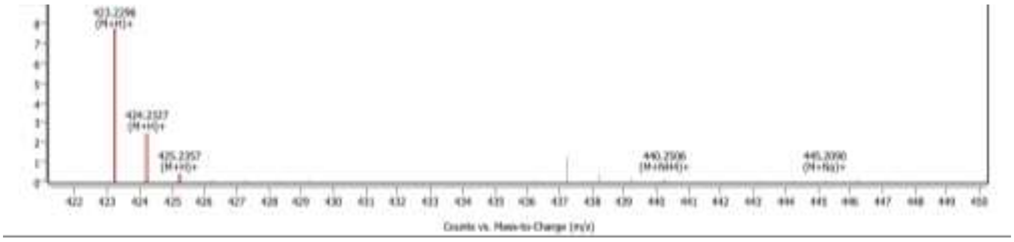

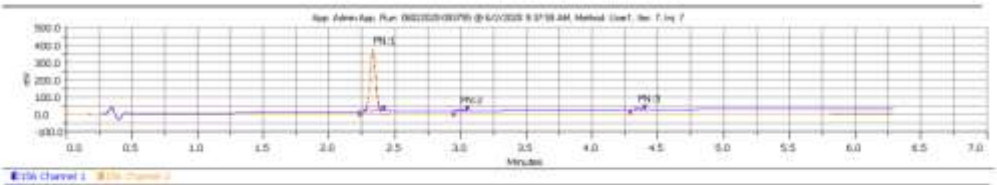

Sample Table

Injection Number	Sample Name	Sample Location	Peak Name	Retention Time (min)	Area ([illegible])	Area %	Height ([illegible])	Plate Number	
[illegible]	[illegible]	[illegible]	1	[illegible]	[illegible]	[illegible]	[illegible]	[illegible]	
[illegible]	[illegible]	[illegible]	2	[illegible]	[illegible]	[illegible]	[illegible]	[illegible]	
[illegible]	[illegible]	[illegible]	3	[illegible]	[illegible]	[illegible]	[illegible]	[illegible]	

^{1}H NMR, ^{13}C NMR, HRMS and HPLC spectra of **15d**

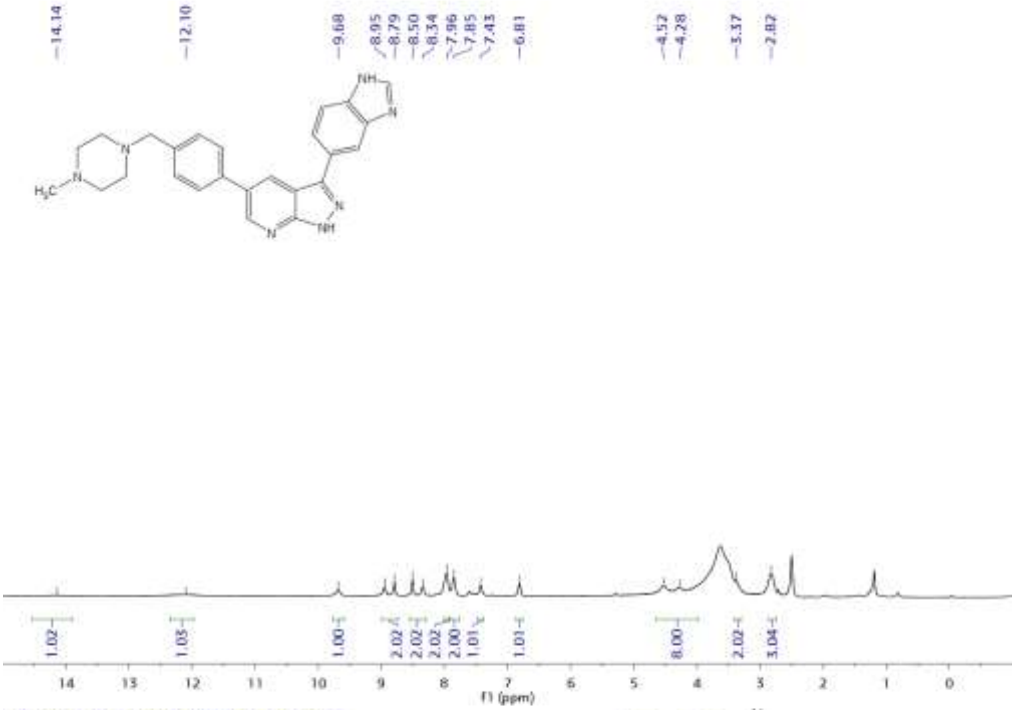

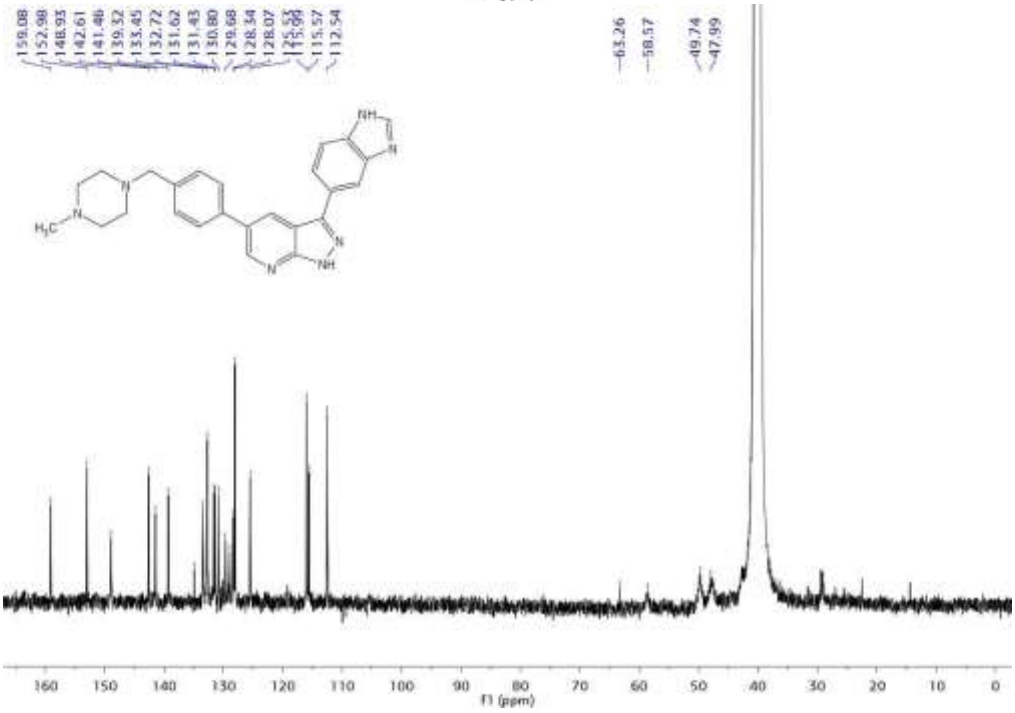

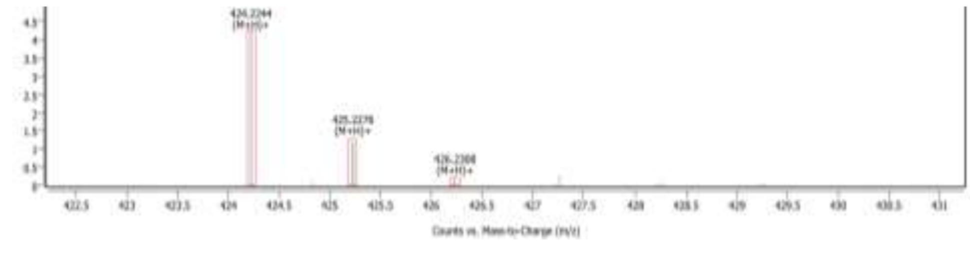

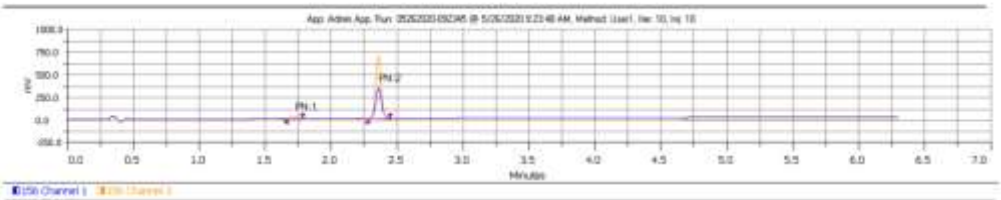

Sample Table

Injection Number	Sample Name	Sample Location	Peak Name	Retention Time (min)	Area [illegible]	Area %	Height (mV)	Plate Number	
10	[illegible]	[illegible]	1	[illegible]	[illegible]	[illegible]	[illegible]	[illegible]	
10	[illegible]	[illegible]	2	[illegible]	[illegible]	[illegible]	[illegible]	[illegible]	

^{1}H NMR, ^{13}C NMR, HRMS and HPLC spectra of **15e**

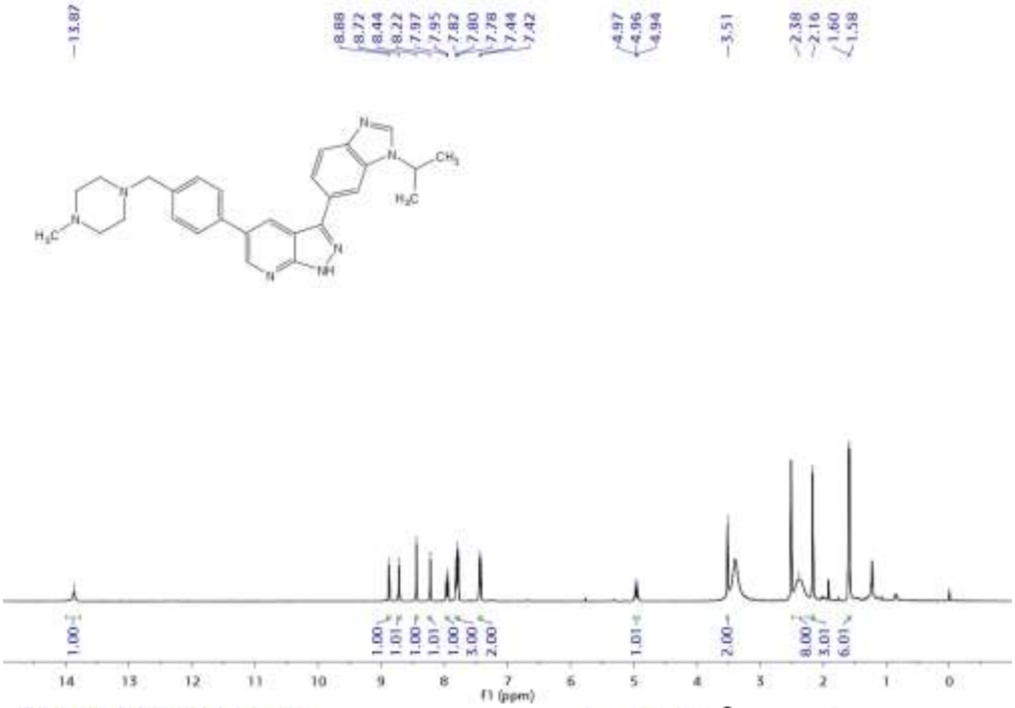

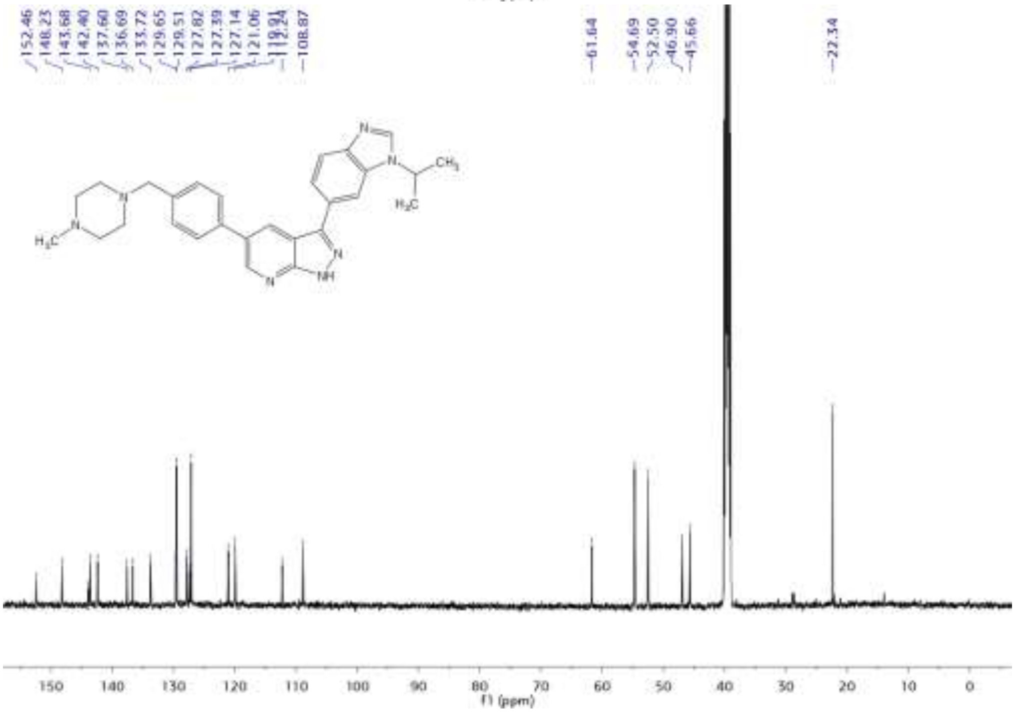

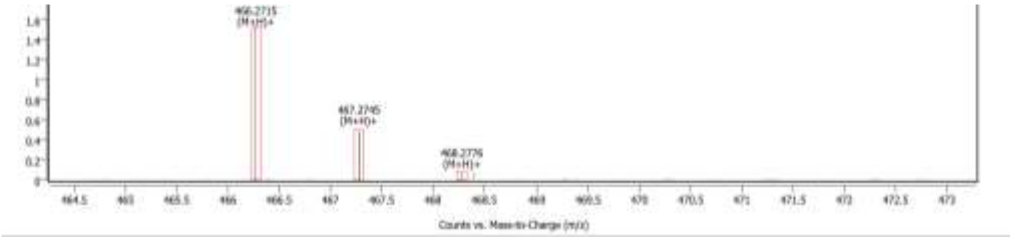

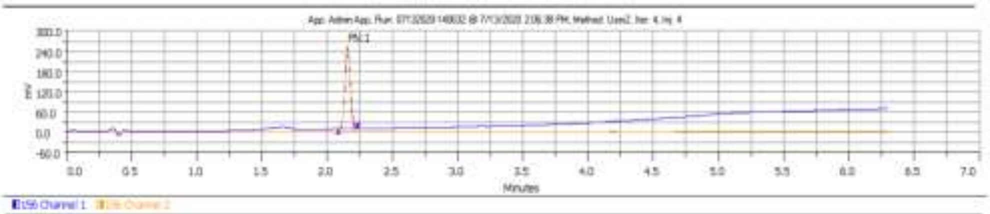

Sample Table

Injection Number	Sample Name	Sample Location	Peak Name	Retention Time (min)	Area (uV*min x100)	Area %	Height (mV)	Plate Number
4	[illegible]	[illegible]	1	[illegible]	[illegible]	100	[illegible]	[illegible]

^{1}H NMR, ^{13}C NMR, HRMS and HPLC spectra of **15f**

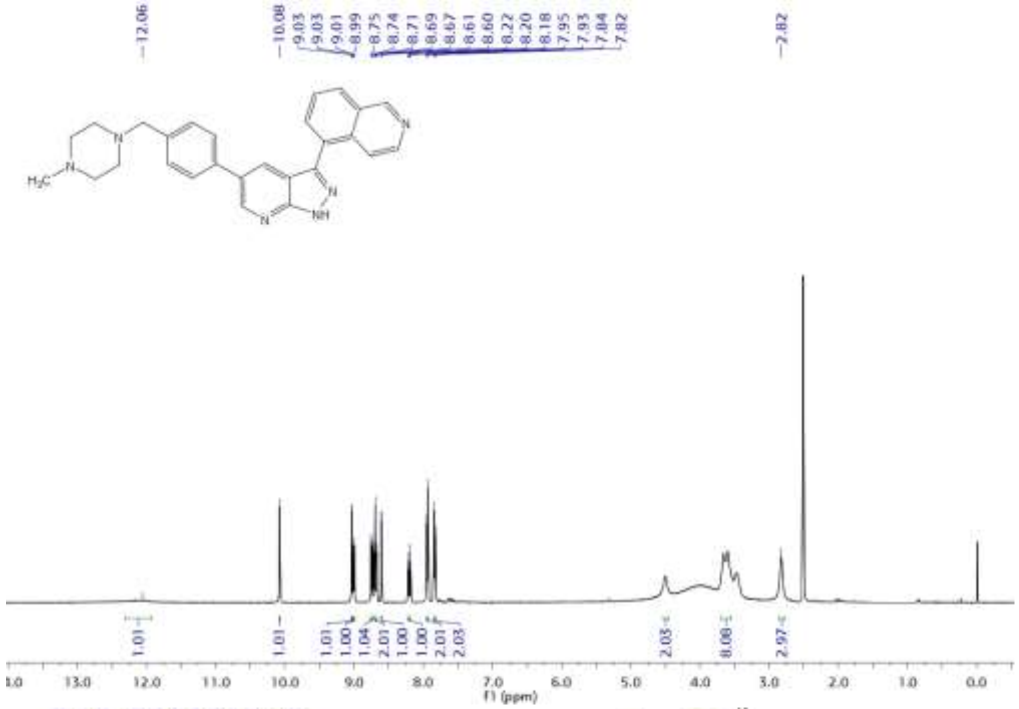

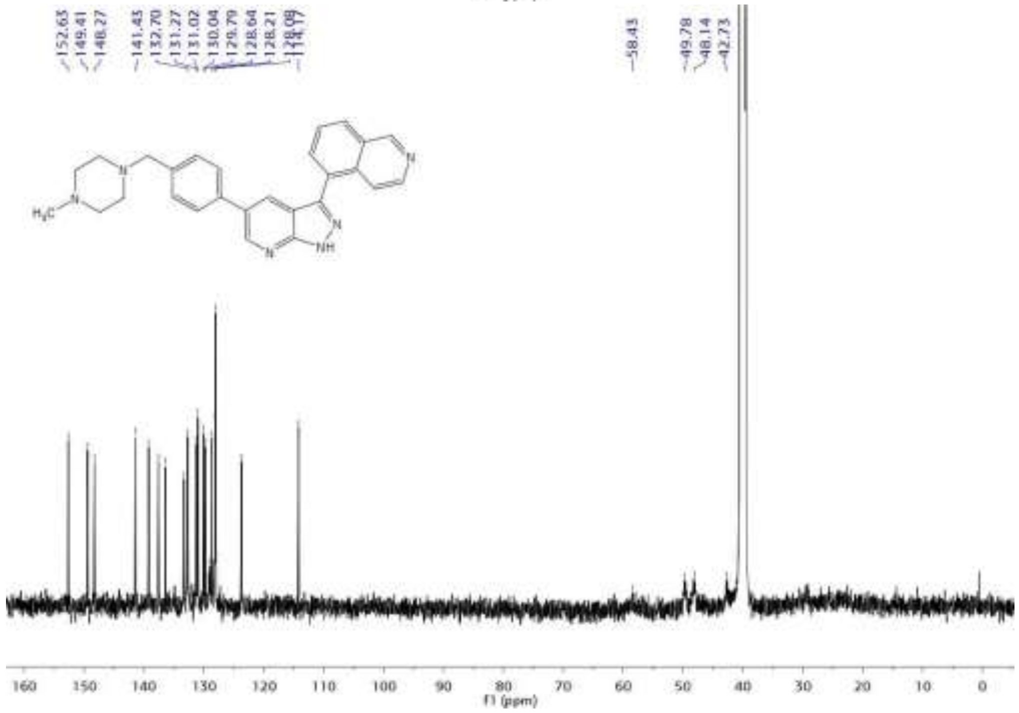

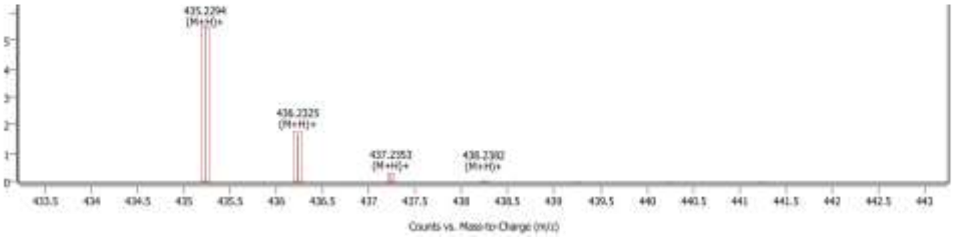

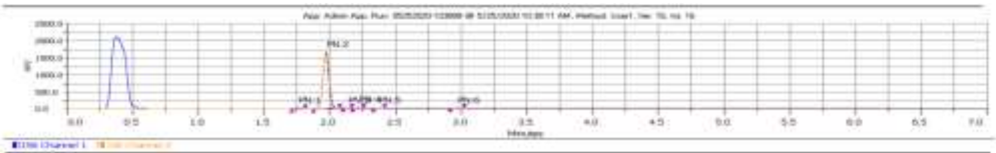

Sample Table

[illegible]	[illegible]	[illegible]	[illegible]	[illegible]	[illegible]	Area %	[illegible]	[illegible]
[illegible]	[illegible]	[illegible]	1	[illegible]	[illegible]	[illegible]	[illegible]	[illegible]
[illegible]	[illegible]	[illegible]	2	[illegible]	[illegible]	[illegible]	[illegible]	[illegible]
[illegible]	[illegible]	[illegible]	3	[illegible]	[illegible]	[illegible]	[illegible]	[illegible]
[illegible]	[illegible]	[illegible]	4	[illegible]	[illegible]	[illegible]	[illegible]	[illegible]
[illegible]	[illegible]	[illegible]	5	[illegible]	[illegible]	[illegible]	[illegible]	[illegible]
[illegible]	[illegible]	[illegible]	6	[illegible]	[illegible]	[illegible]	[illegible]	[illegible]

^{1}H NMR, ^{13}C NMR, HRMS and HPLC spectra of **15g**

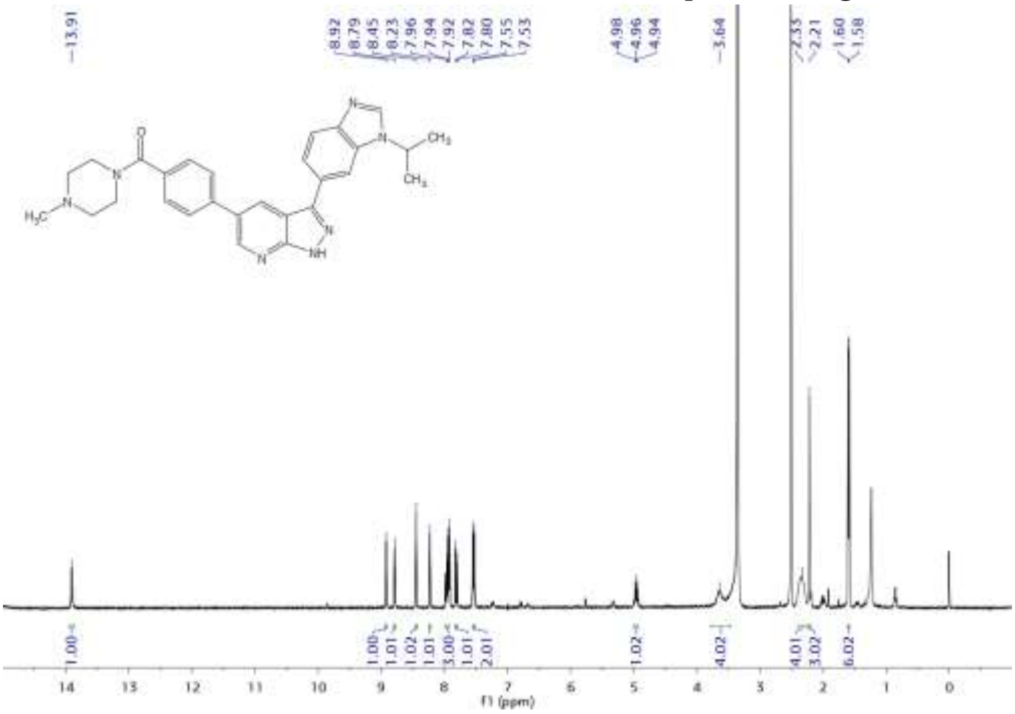

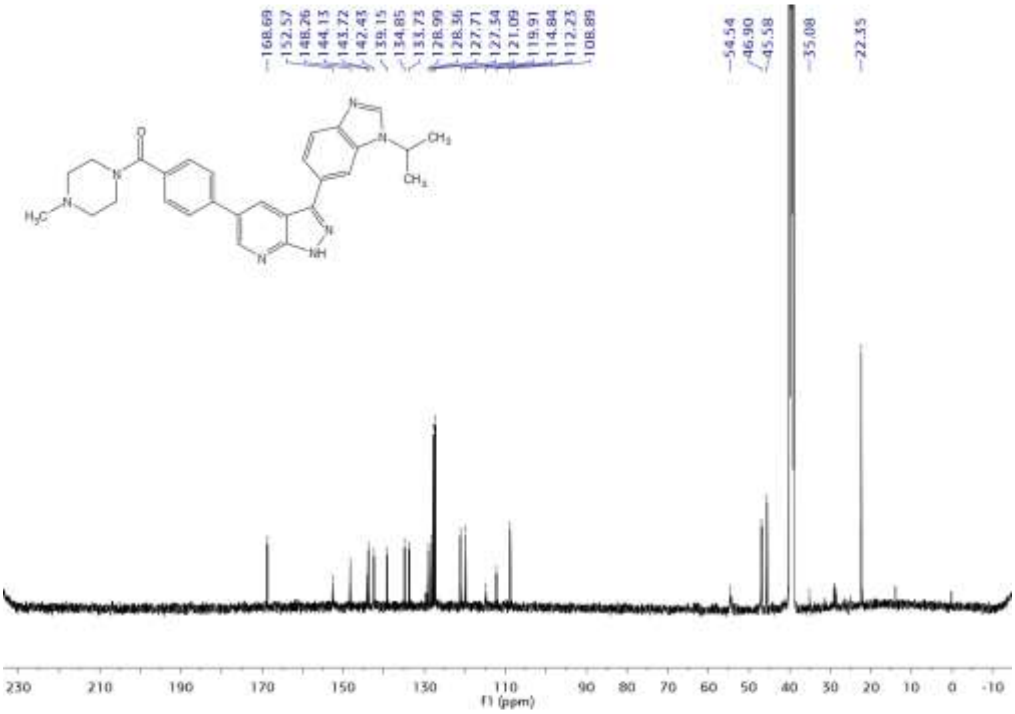

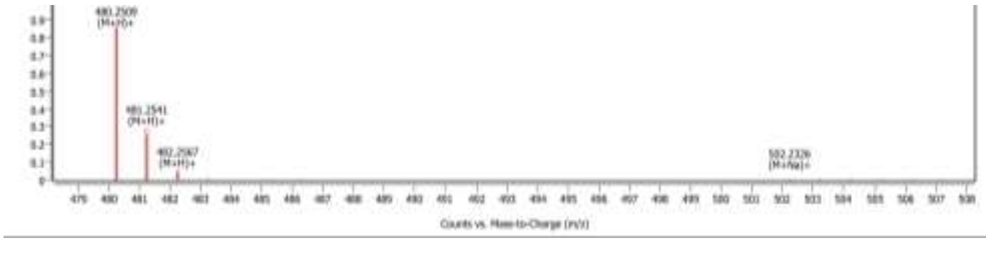

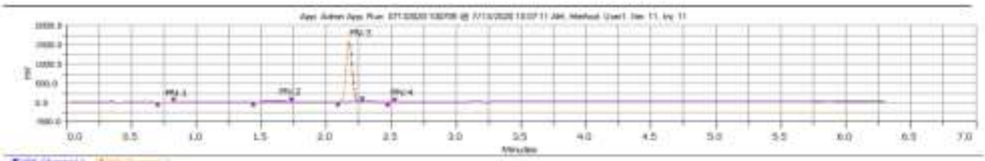

Sample Table

Injection Number	Sample Name	Sample Location	Peak Name	Retention Time (min)	Area [illegible]	Area %	Height (mV)	Plate Number
11	[illegible]	[illegible]	1	[illegible]	[illegible]	[illegible]	[illegible]	[illegible]
11	[illegible]	[illegible]	2	[illegible]	[illegible]	[illegible]	[illegible]	[illegible]
11	[illegible]	[illegible]	3	[illegible]	[illegible]	[illegible]	[illegible]	[illegible]
11	[illegible]	[illegible]	4	[illegible]	[illegible]	[illegible]	[illegible]	[illegible]

^{1}H NMR, ^{13}C NMR, HRMS and HPLC spectra of **15h**

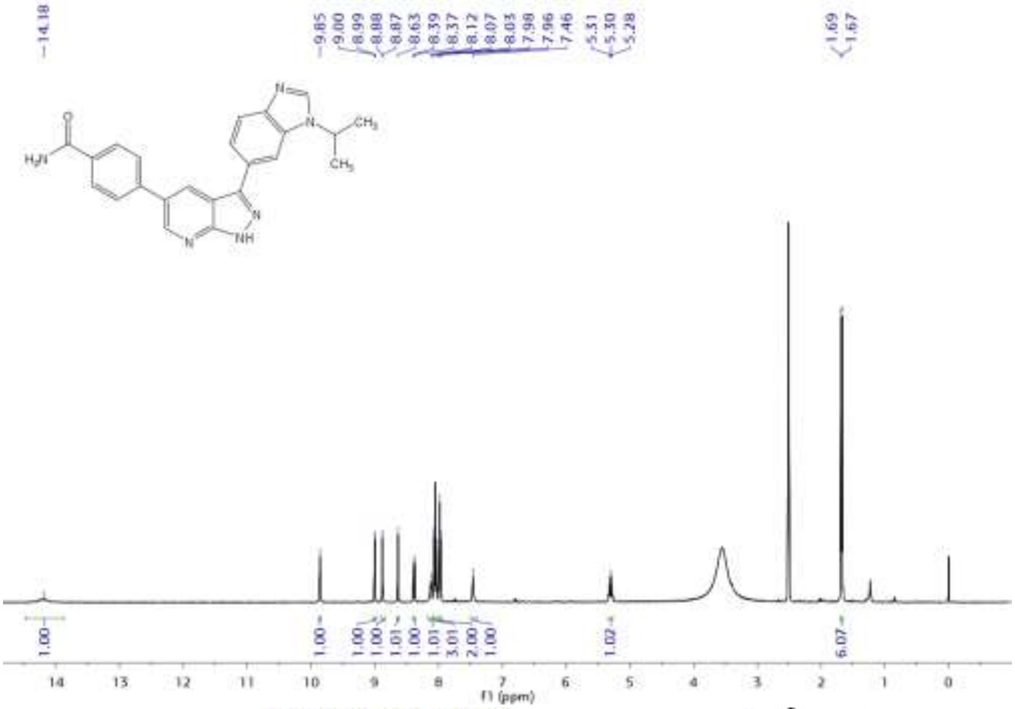

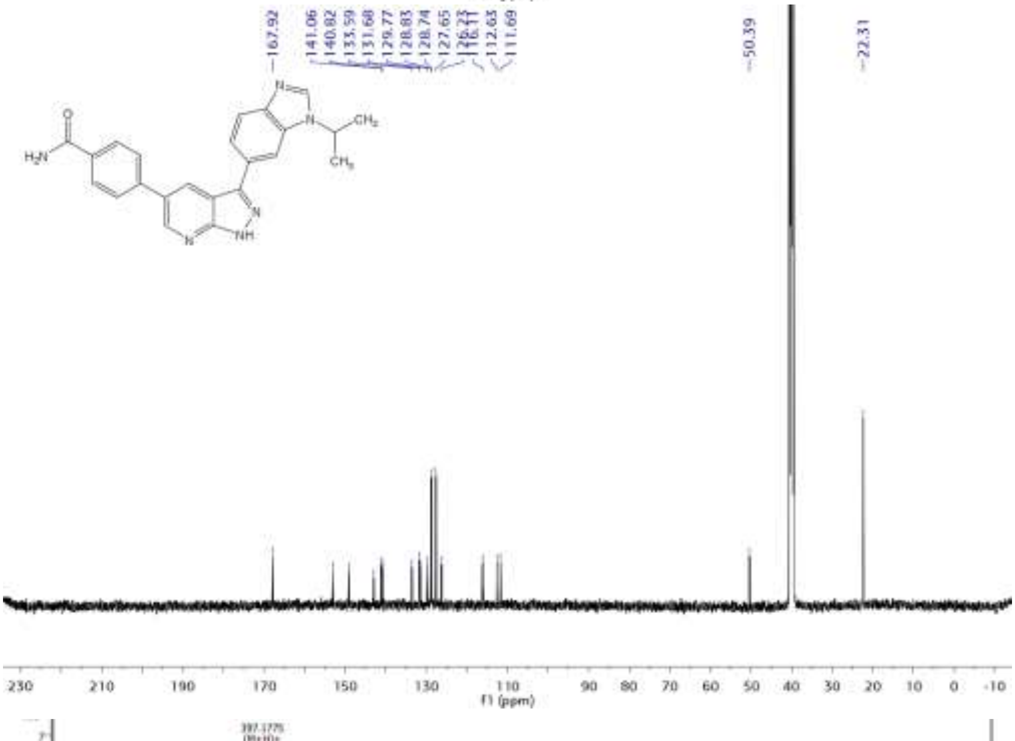

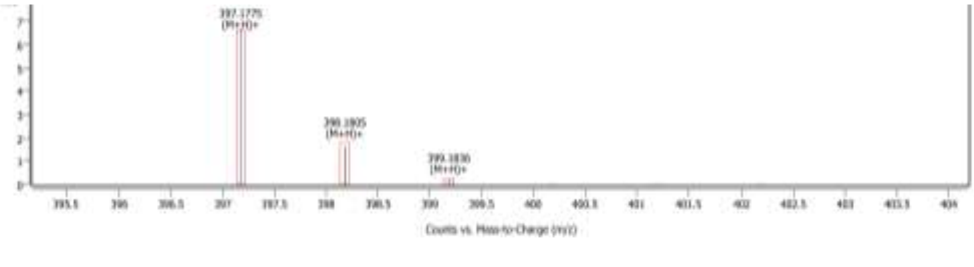

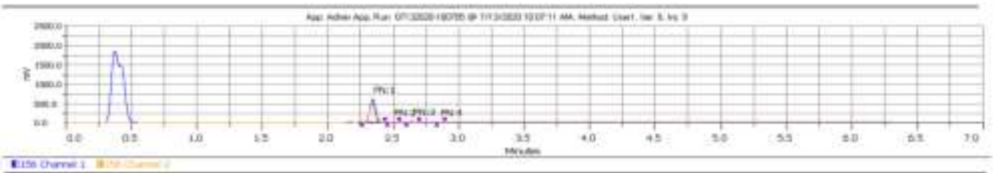

Sample Table

Injection Number	Sample Name	Sample Location	Peak Name	Retention Time (min)	Area [illegible]	Area %	Height (mV)	Plate Number	
[illegible]	[illegible]	[illegible]	1	[illegible]	[illegible]	[illegible]	[illegible]	[illegible]	
[illegible]	[illegible]	[illegible]	2	[illegible]	[illegible]	[illegible]	[illegible]	[illegible]	
[illegible]	[illegible]	[illegible]	3	[illegible]	[illegible]	[illegible]	[illegible]	[illegible]	
[illegible]	[illegible]	[illegible]	4	[illegible]	[illegible]	[illegible]	[illegible]	[illegible]	

^{1}H NMR, ^{13}C NMR, HRMS and HPLC spectra of **15i**

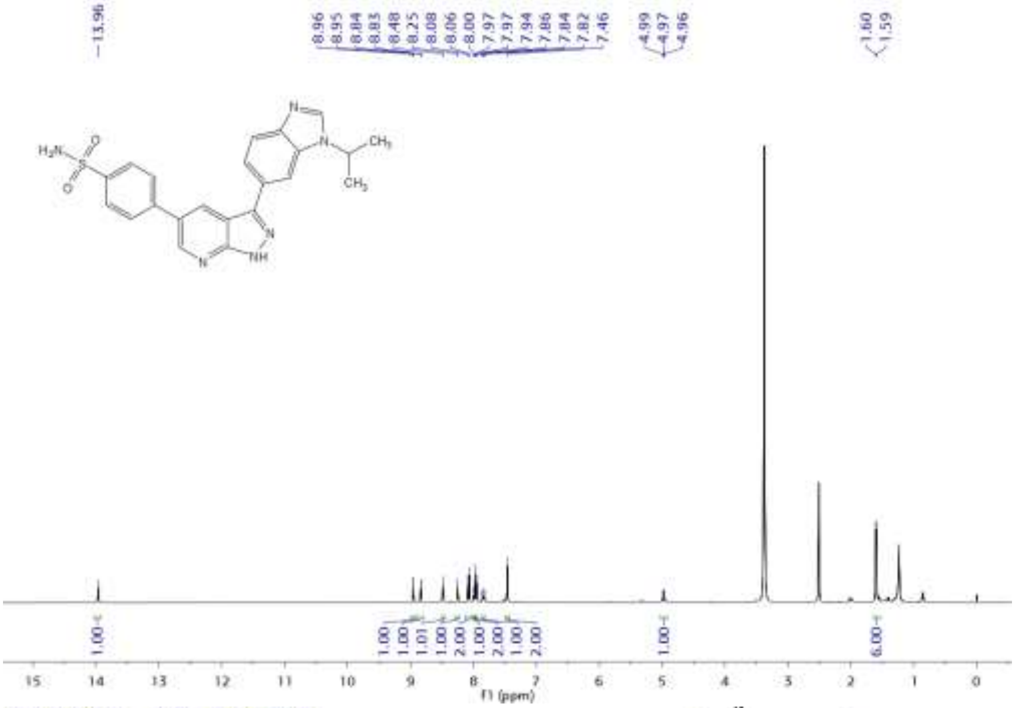

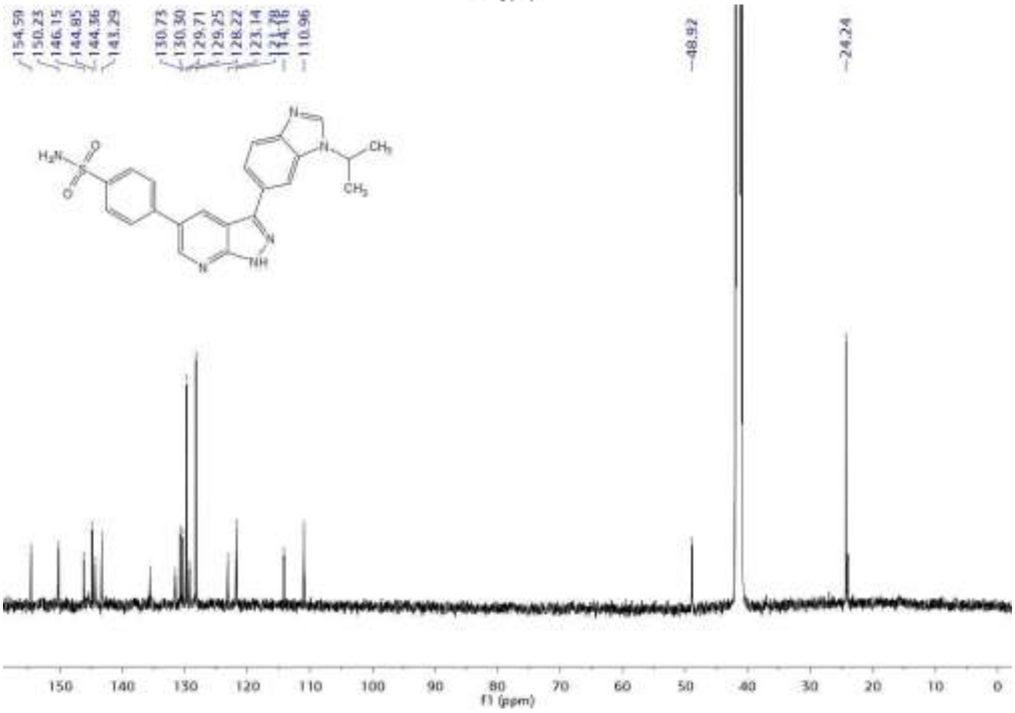

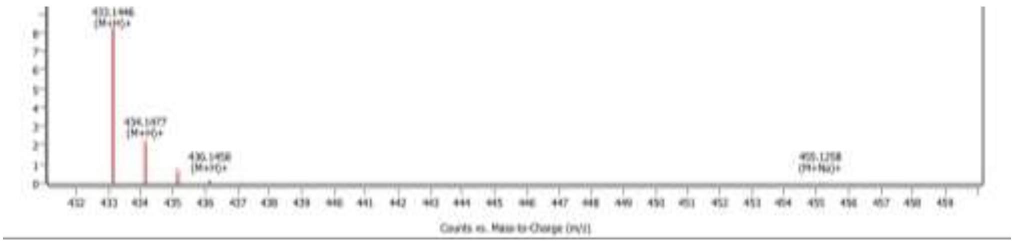

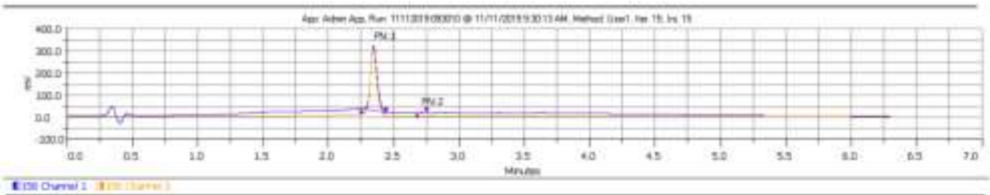

Sample Table

Injection Number	Sample Name	Sample Location	Peak Name	Retention Time (min)	Area (uV*min x100)	Area %	Height (mV)	Plate Number	
[illegible]	[illegible]	[illegible]	1	[illegible]	[illegible]	[illegible]	[illegible]	[illegible]	
[illegible]	[illegible]	[illegible]	2	[illegible]	[illegible]	[illegible]	[illegible]	[illegible]	

^{1}H NMR, ^{13}C NMR, HRMS and HPLC spectra of **15j**

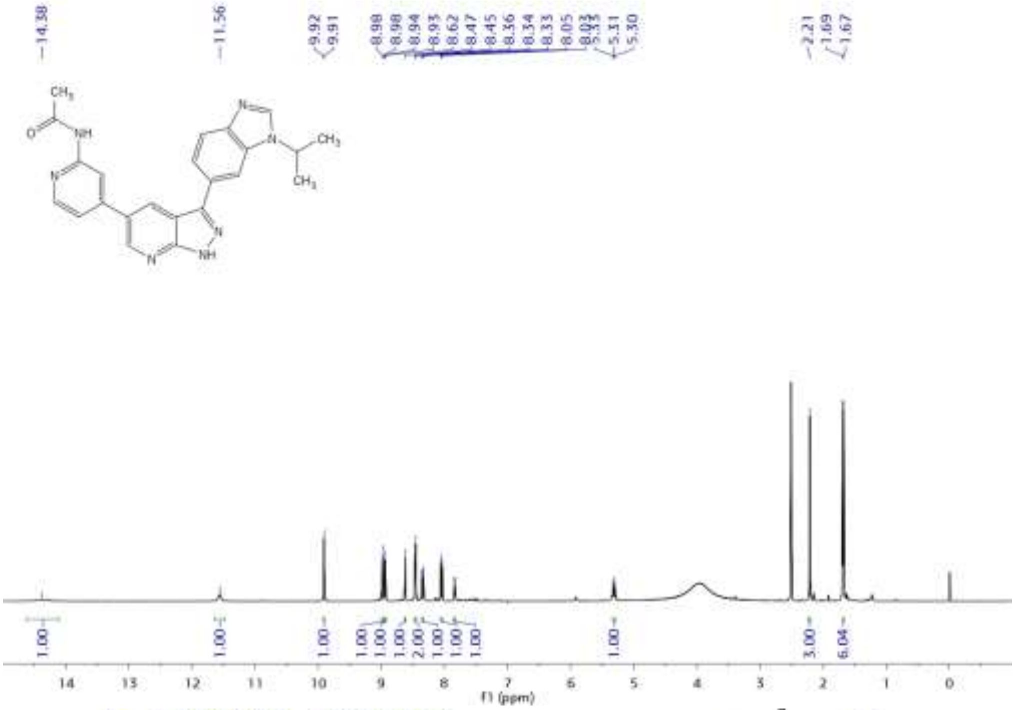

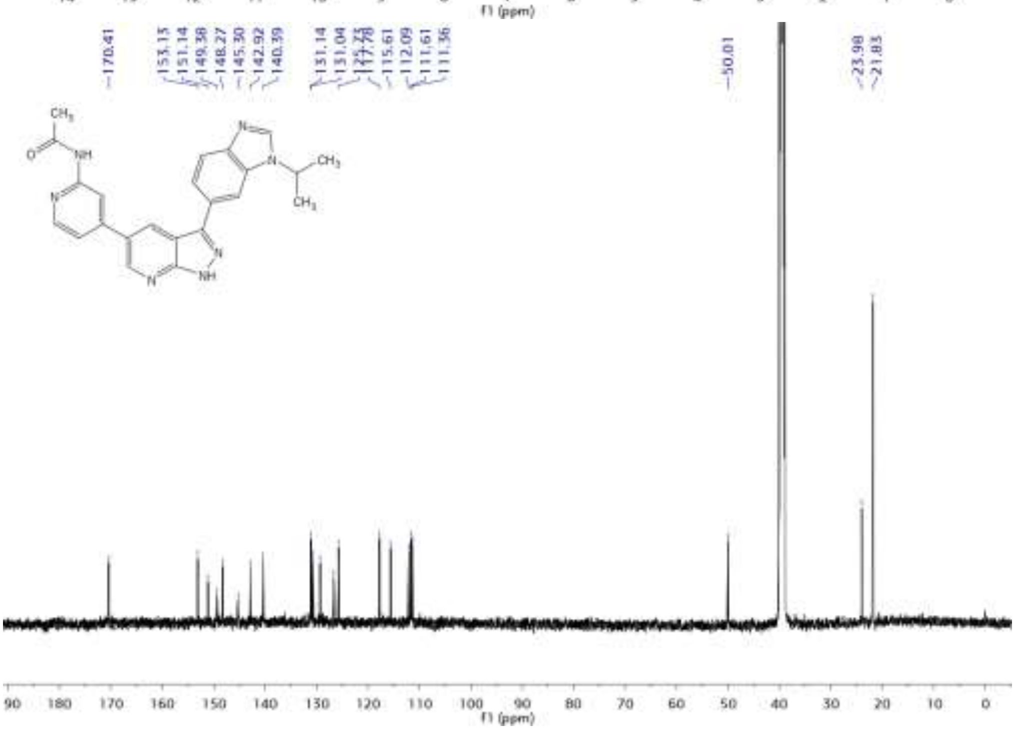

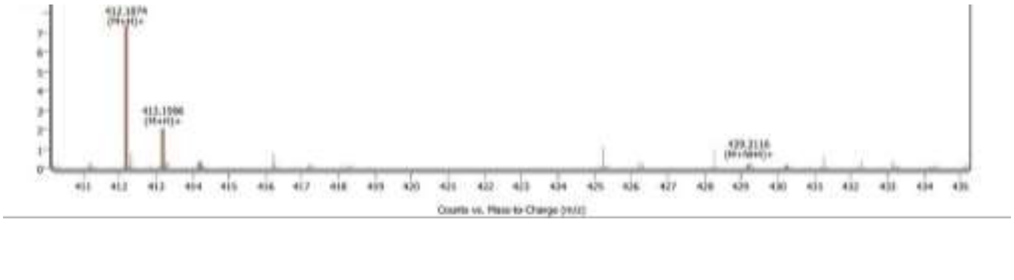

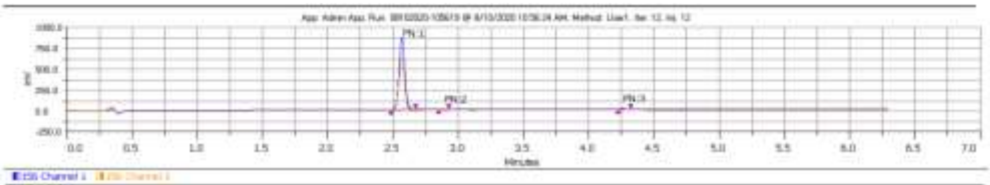

Sample Table

Injection Number	Sample Name	Sample Location	Peak Name	Retention Time (min)	Area [illegible]	Area %	Height (mV)	Plate Number	
12	[illegible]	[illegible]	1	2.57	[illegible]	[illegible]	[illegible]	[illegible]	
12	[illegible]	[illegible]	2	[illegible]	[illegible]	[illegible]	[illegible]	[illegible]	
12	[illegible]	[illegible]	3	[illegible]	[illegible]	[illegible]	2.6	[illegible]	

^{1}H NMR, ^{13}C NMR, HRMS and HPLC spectra of **15k**

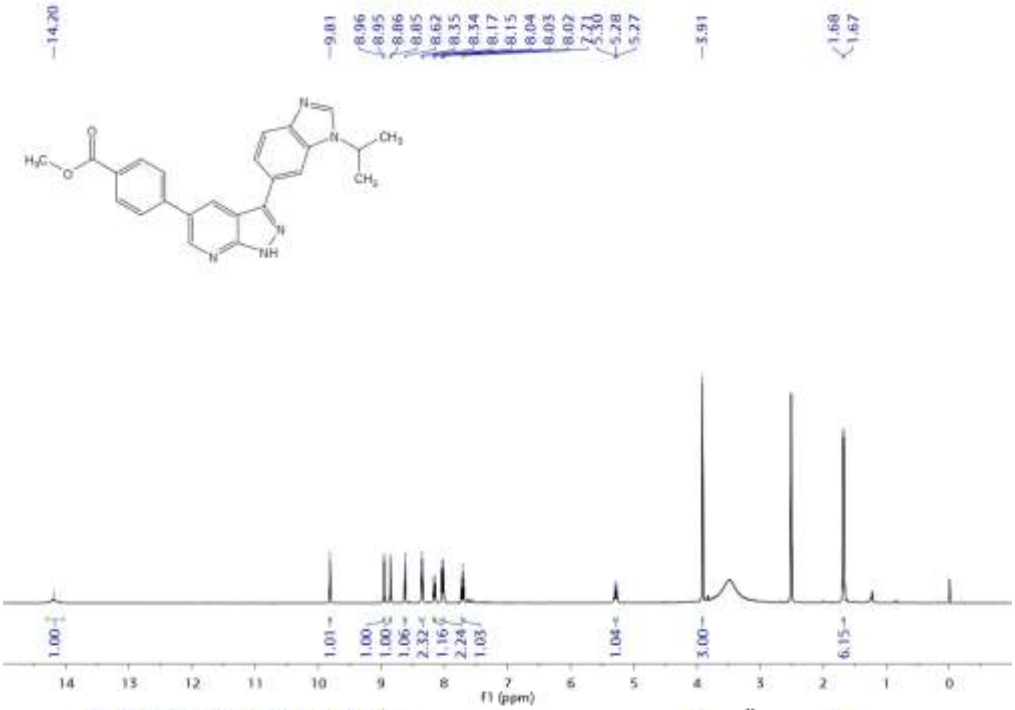

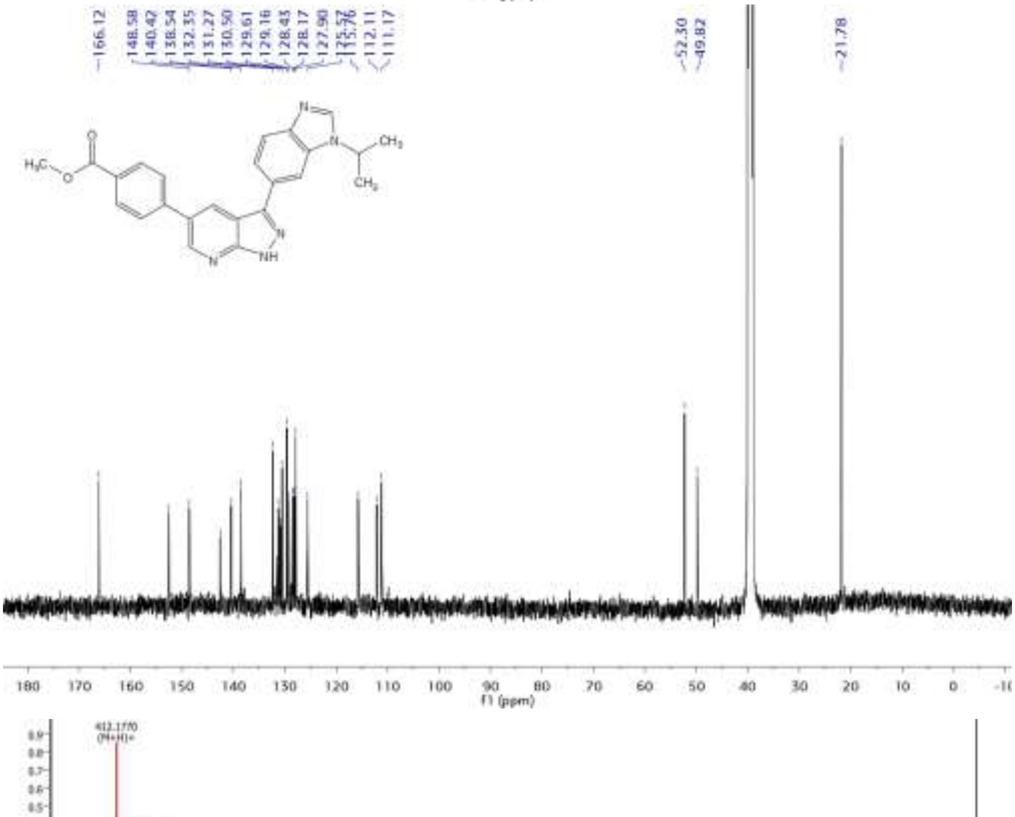

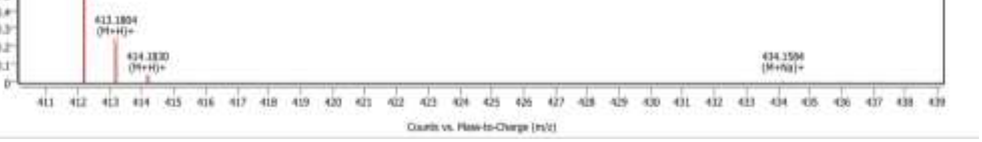

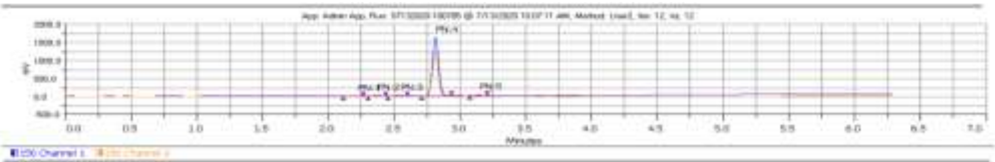

Sample Table

Injection Number	Sample Name	Sample Location	Peak Name	Retention Time (min)	Area (uV*sec x1000)	Area %	Height (uV)	Plate Number	
[illegible]	[illegible]	[illegible]	1	[illegible]	[illegible]	[illegible]	[illegible]	[illegible]	
[illegible]	[illegible]	[illegible]	2	[illegible]	[illegible]	[illegible]	[illegible]	[illegible]	
[illegible]	[illegible]	[illegible]	3	[illegible]	[illegible]	[illegible]	[illegible]	[illegible]	
[illegible]	[illegible]	[illegible]	4	[illegible]	[illegible]	[illegible]	[illegible]	[illegible]	
[illegible]	[illegible]	[illegible]	5	[illegible]	[illegible]	[illegible]	[illegible]	[illegible]	

^{1}H NMR, ^{13}C NMR, HRMS and HPLC spectra of **15l**

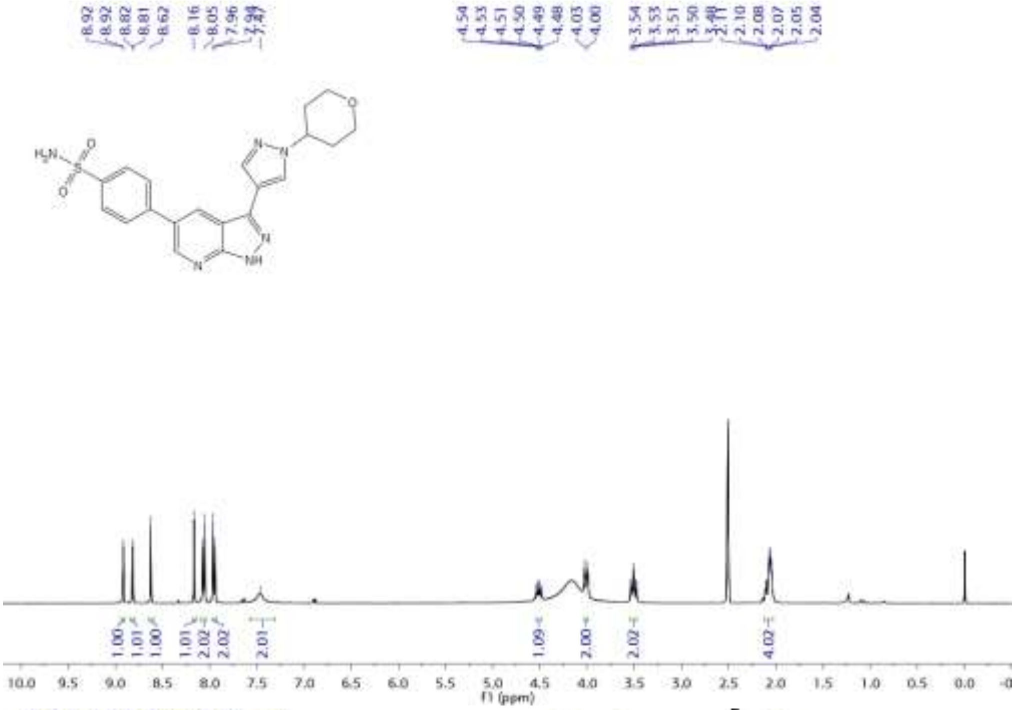

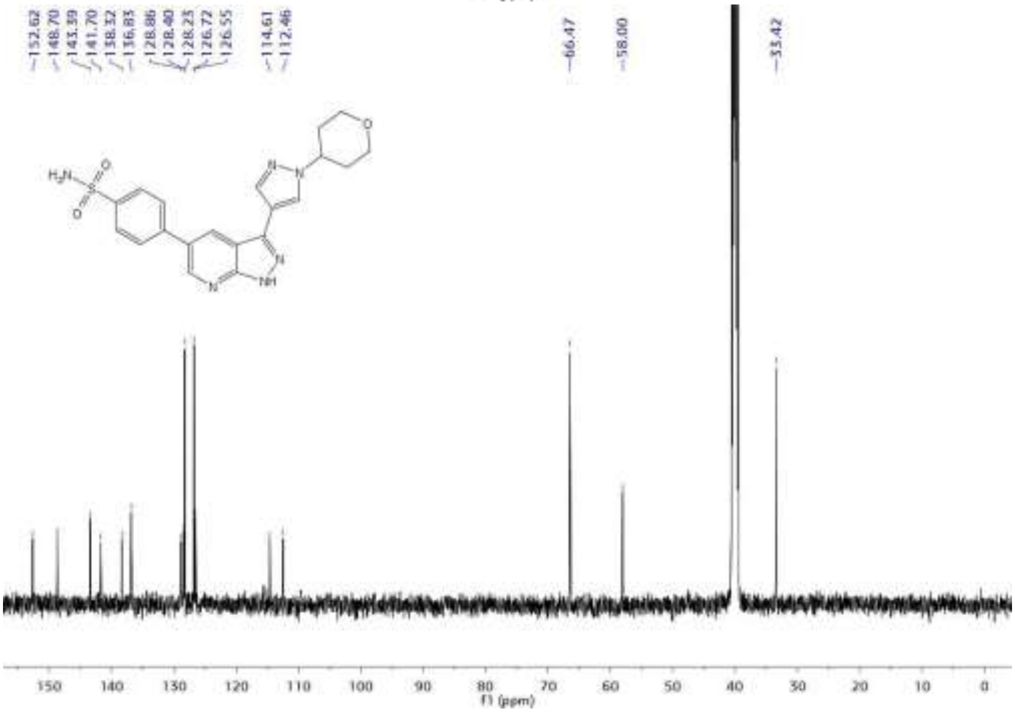

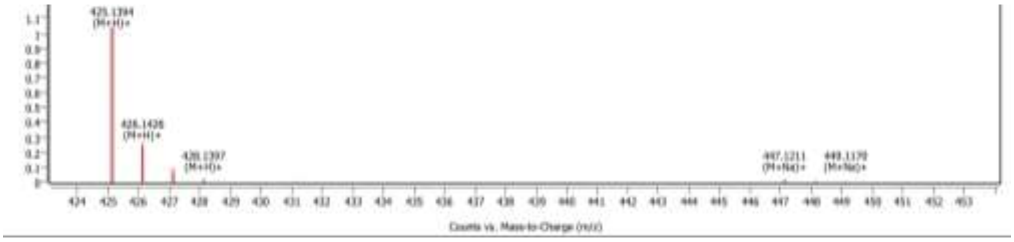

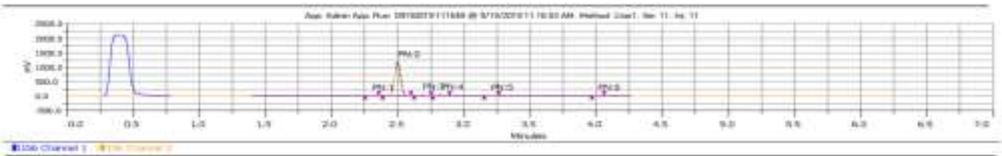

Sample Table

Injection Number	Sample Name	Sample Location	Peak Name	Retention Time (min)	Area ([illegible])	Area %	Height ([illegible])	Plate Number	
11	[illegible]	[illegible]	1	[illegible]	[illegible]	[illegible]	[illegible]	[illegible]	
11	[illegible]	[illegible]	2	[illegible]	[illegible]	[illegible]	[illegible]	[illegible]	
11	[illegible]	[illegible]	3	[illegible]	[illegible]	[illegible]	[illegible]	[illegible]	
11	[illegible]	[illegible]	4	[illegible]	[illegible]	[illegible]	[illegible]	[illegible]	
11	[illegible]	[illegible]	5	[illegible]	[illegible]	[illegible]	[illegible]	[illegible]	
11	[illegible]	[illegible]	6	[illegible]	[illegible]	[illegible]	[illegible]	[illegible]	

^{1}H NMR, ^{13}C NMR, HRMS and HPLC spectra of **15m**

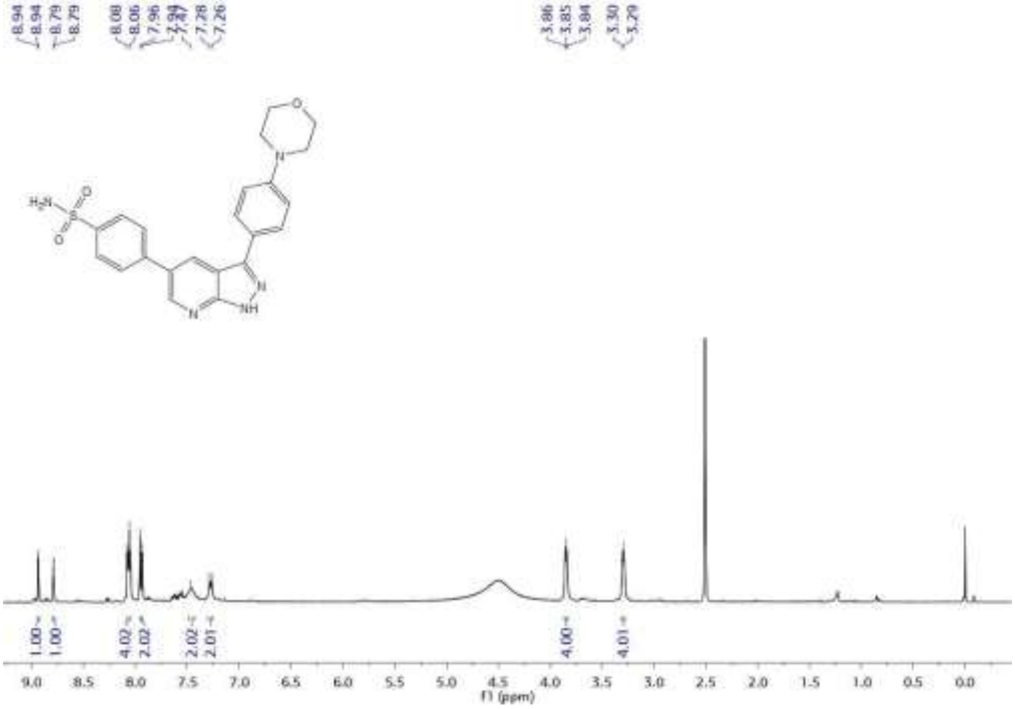

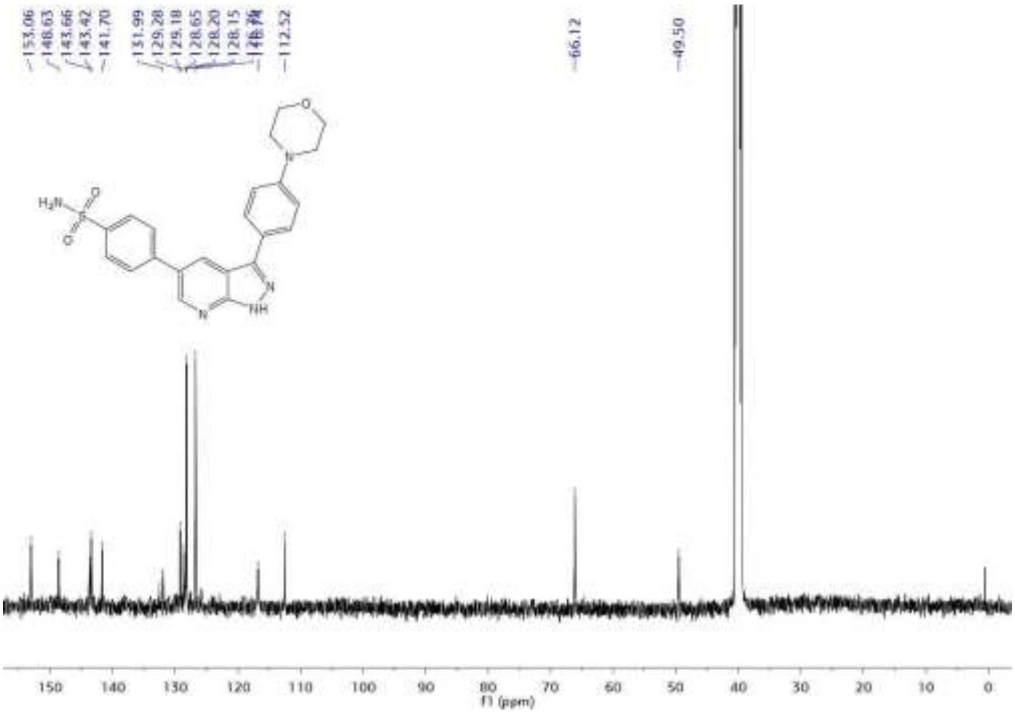

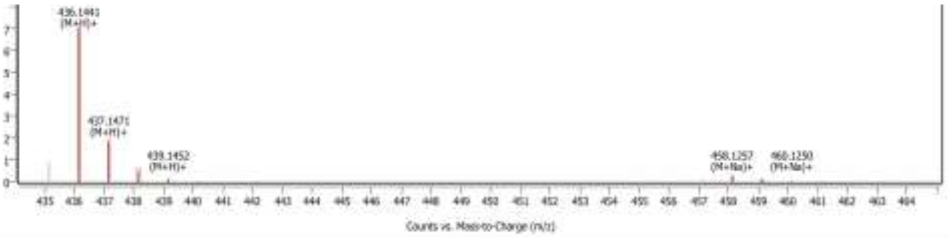

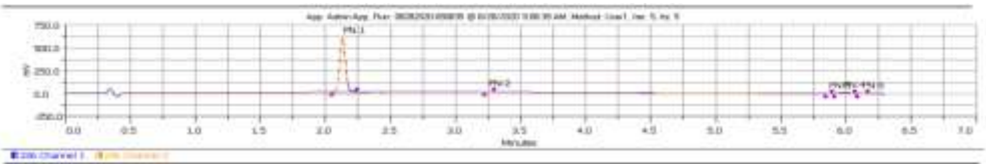

Sample Table

Injection Number	Sample Name	Sample Location	Peak Name	Retention Time (min)	Area ([illegible])	Area %	Height (mV)	Plate Number
[illegible]	[illegible]	[illegible]	1	[illegible]	[illegible]	[illegible]	[illegible]	[illegible]
[illegible]	[illegible]	[illegible]	2	[illegible]	[illegible]	[illegible]	[illegible]	[illegible]
[illegible]	[illegible]	[illegible]	3	[illegible]	[illegible]	[illegible]	[illegible]	[illegible]
[illegible]	[illegible]	[illegible]	4	[illegible]	[illegible]	[illegible]	[illegible]	[illegible]
[illegible]	[illegible]	[illegible]	5	[illegible]	[illegible]	[illegible]	[illegible]	[illegible]

^{1}H NMR, ^{13}C NMR, HRMS and HPLC spectra of **15n**

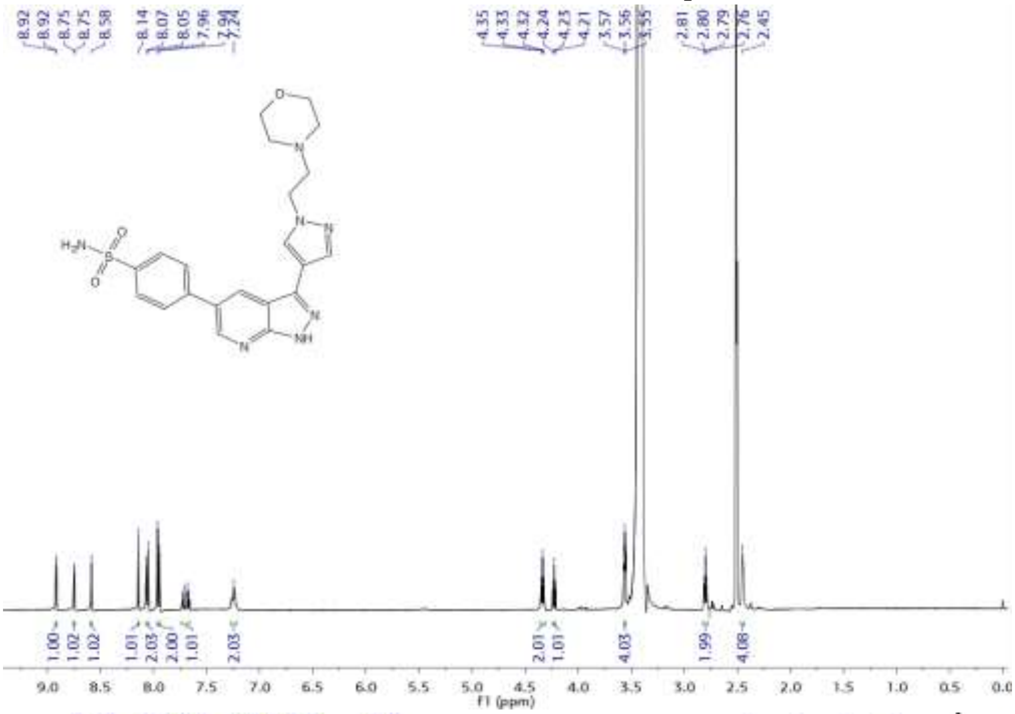

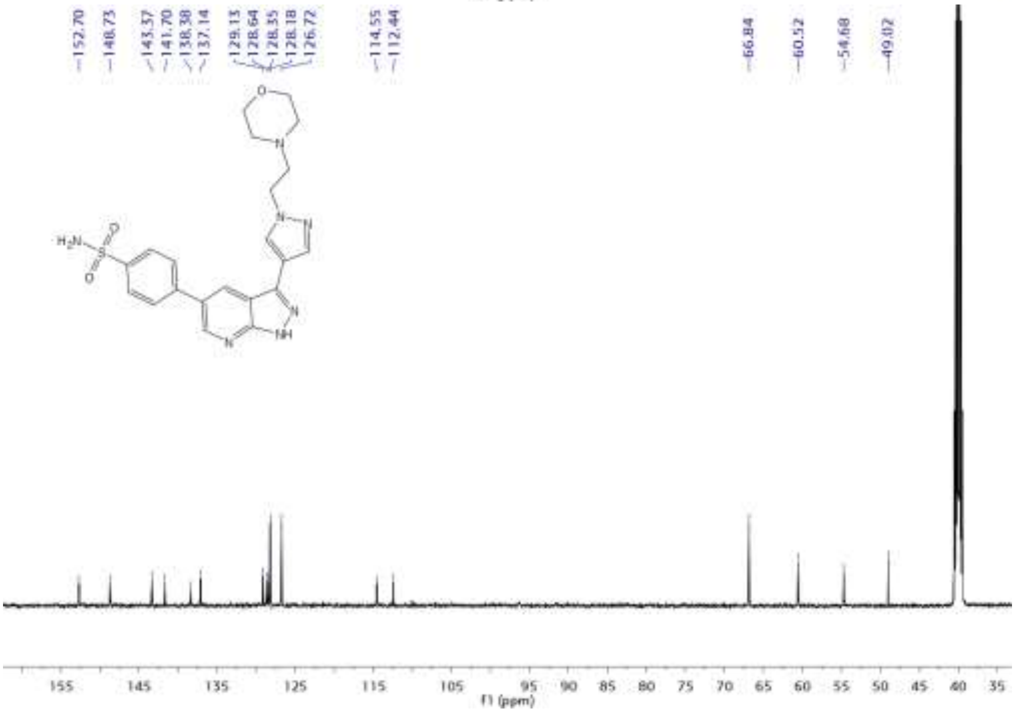

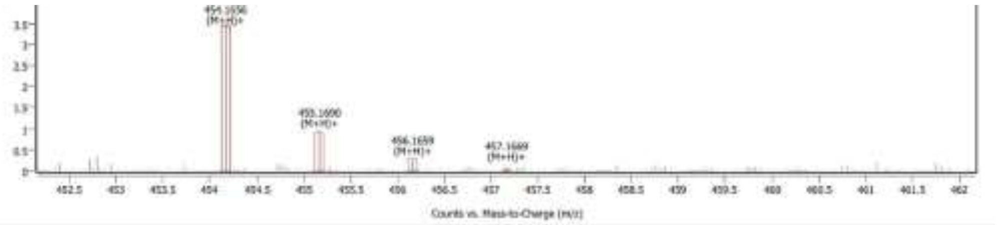

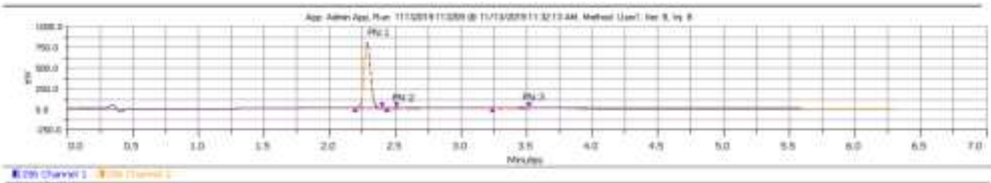

Sample Table

Injection Number	Sample Name	Sample Location	Peak Name	Retention Time (min)	Area (uV*sec x100)	Area %	Height (mV)	Plate Number
[illegible]	[illegible]	[illegible]	1	2.280	[illegible]	[illegible]	[illegible]	[illegible]
[illegible]	[illegible]	[illegible]	2	[illegible]	[illegible]	[illegible]	[illegible]	[illegible]
[illegible]	[illegible]	[illegible]	3	[illegible]	[illegible]	[illegible]	[illegible]	[illegible]

^{1}H NMR, ^{13}C NMR, HRMS and HPLC spectra of **15o**

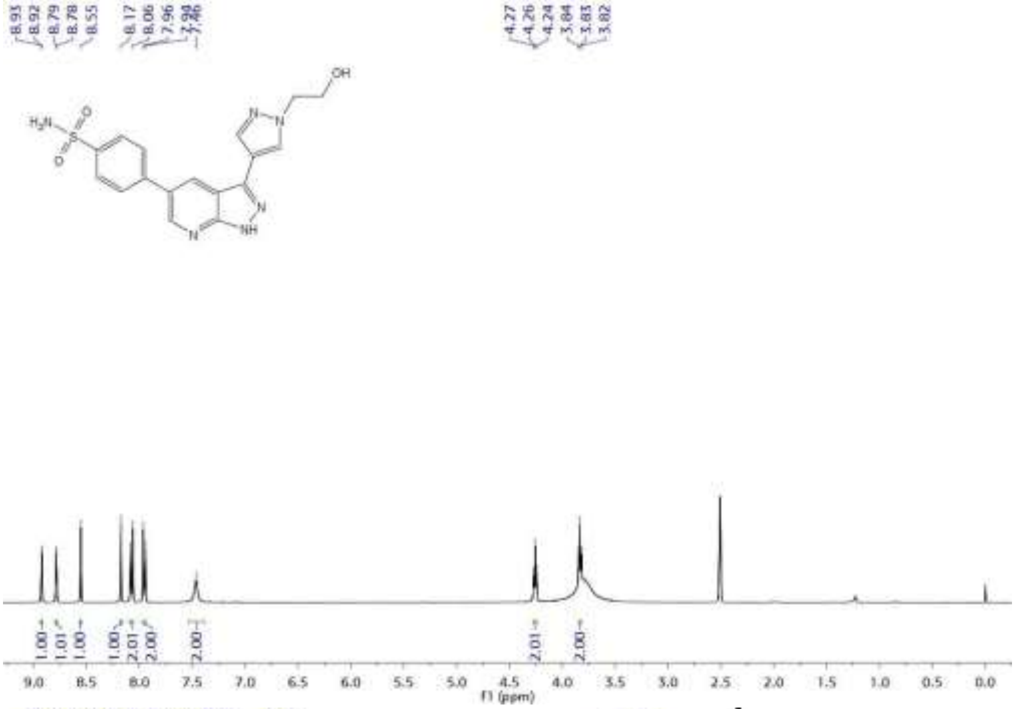

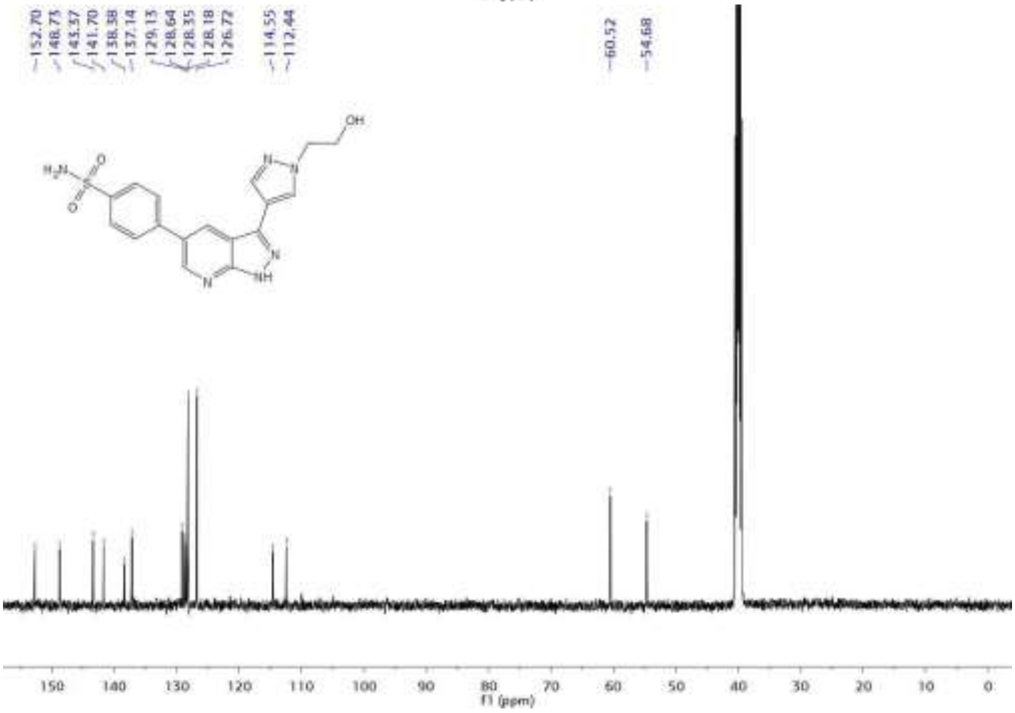

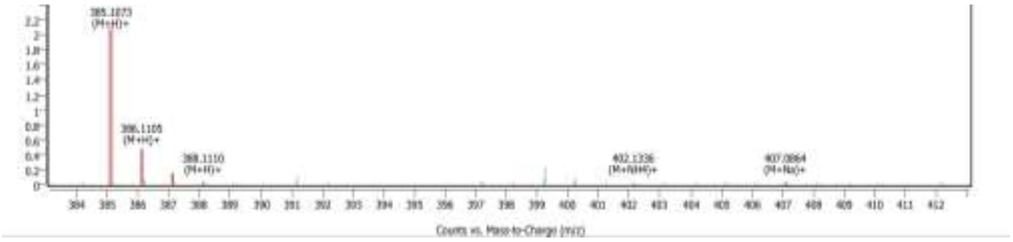

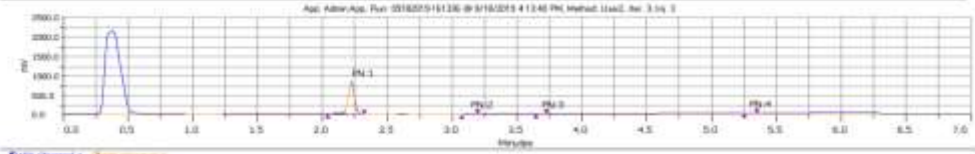

Sample Table

Injection Number	Sample Name	Sample Location	Peak Name	Retention Time (min)	Area ([illegible])	Area %	Height ([illegible])	Plate Number	
[illegible]	[illegible]	[illegible]	1	[illegible]	[illegible]	[illegible]	[illegible]	[illegible]	
[illegible]	[illegible]	[illegible]	2	[illegible]	[illegible]	[illegible]	[illegible]	[illegible]	
[illegible]	[illegible]	[illegible]	3	[illegible]	[illegible]	[illegible]	[illegible]	[illegible]	
[illegible]	[illegible]	[illegible]	4	[illegible]	[illegible]	[illegible]	[illegible]	[illegible]	

^{1}H NMR, ^{13}C NMR, HRMS and HPLC spectra of **15p**

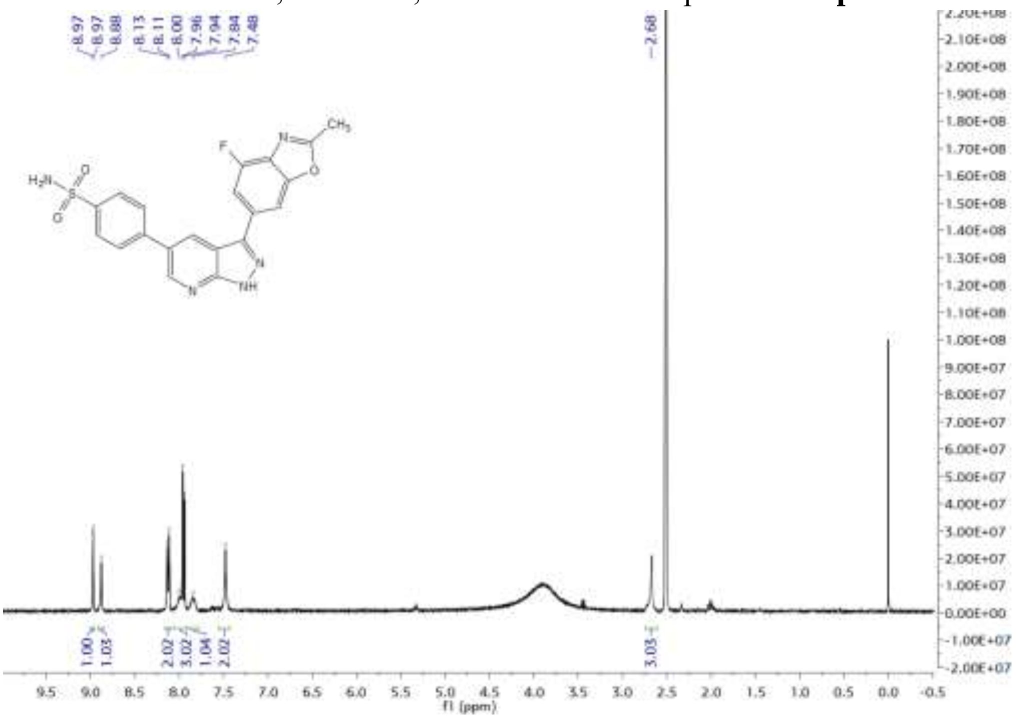

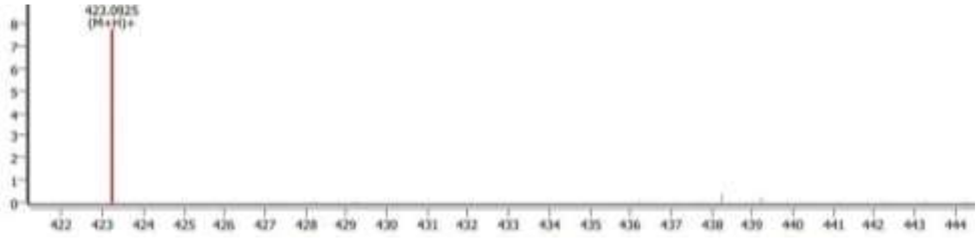

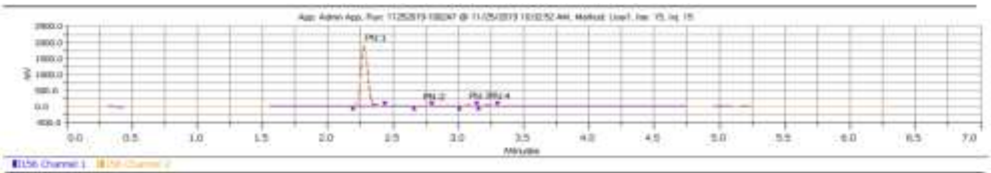

Sample Table

Injection Number	Sample Name	Sample Location	Peak Name	Retention Time (min)	Area (μV*sec x1000)	Area %	Height (μV)	Plate Number
15	[illegible]	[illegible]	1	[illegible]	[illegible]	[illegible]	[illegible]	[illegible]
15	[illegible]	[illegible]	2	[illegible]	[illegible]	[illegible]	[illegible]	[illegible]
15	[illegible]	[illegible]	3	[illegible]	[illegible]	[illegible]	[illegible]	[illegible]
15	[illegible]	[illegible]	4	[illegible]	[illegible]	[illegible]	[illegible]	[illegible]

^{1}H NMR, ^{13}C NMR, HRMS and HPLC spectra of **15q**

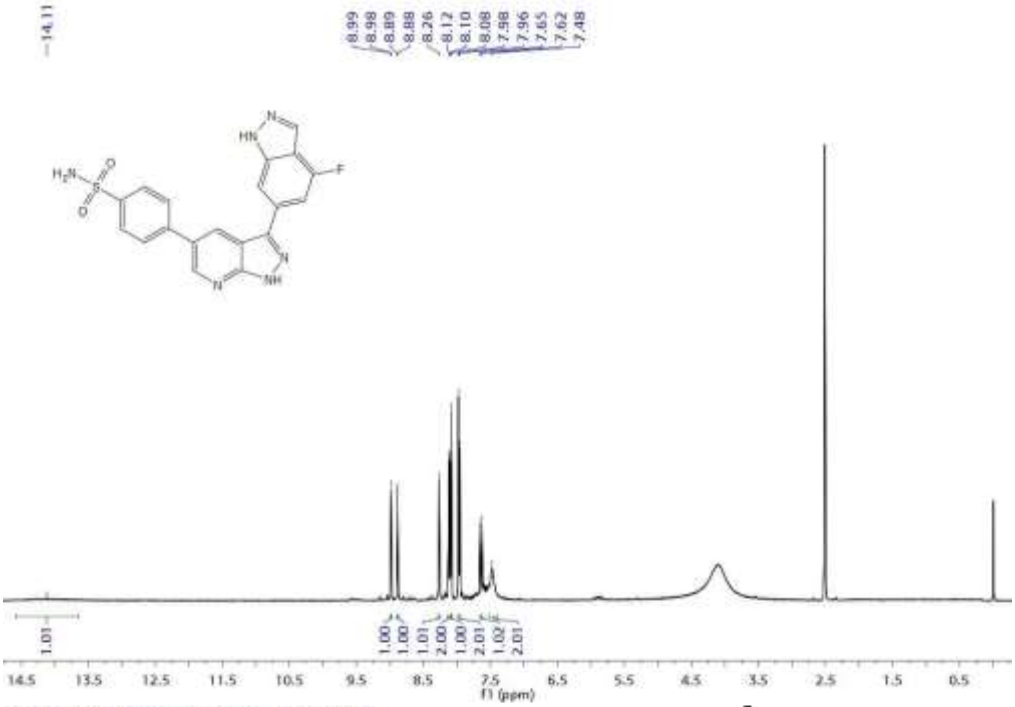

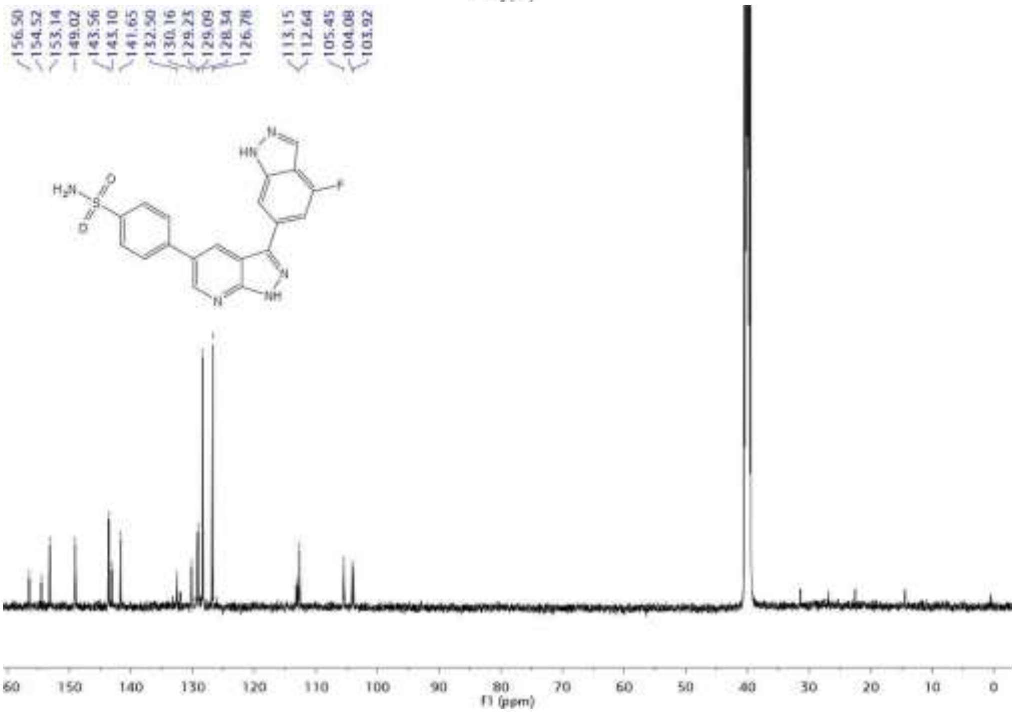

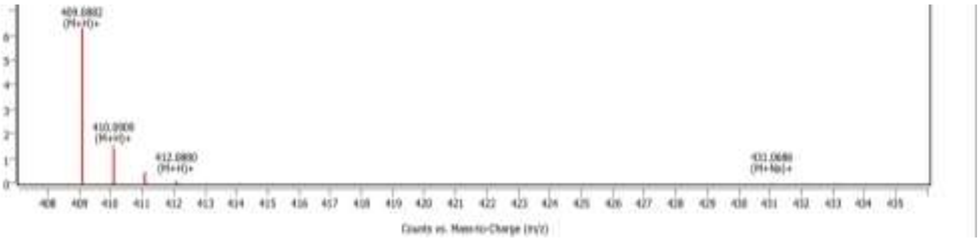

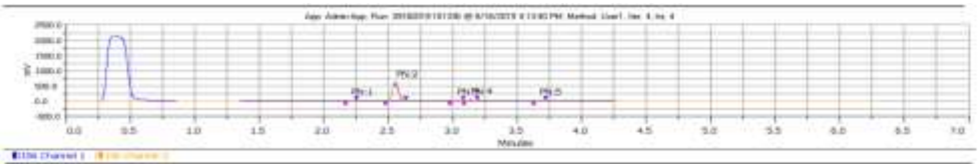

Sample Table

Injection Number	Sample Name	Sample Location	Peak Name	Retention Time (min)	Area (µV*sec x10?)	Area %	Height (µV)	Plate Number	
[illegible]	[illegible]	[illegible]	1	[illegible]	[illegible]	[illegible]	[illegible]	[illegible]	
[illegible]	[illegible]	[illegible]	2	[illegible]	[illegible]	[illegible]	[illegible]	[illegible]	
[illegible]	[illegible]	[illegible]	3	[illegible]	[illegible]	[illegible]	[illegible]	[illegible]	
[illegible]	[illegible]	[illegible]	4	[illegible]	[illegible]	[illegible]	[illegible]	[illegible]	
[illegible]	[illegible]	[illegible]	5	[illegible]	[illegible]	[illegible]	[illegible]	[illegible]	

^{1}H NMR, ^{13}C NMR, HRMS and HPLC spectra of **15r**

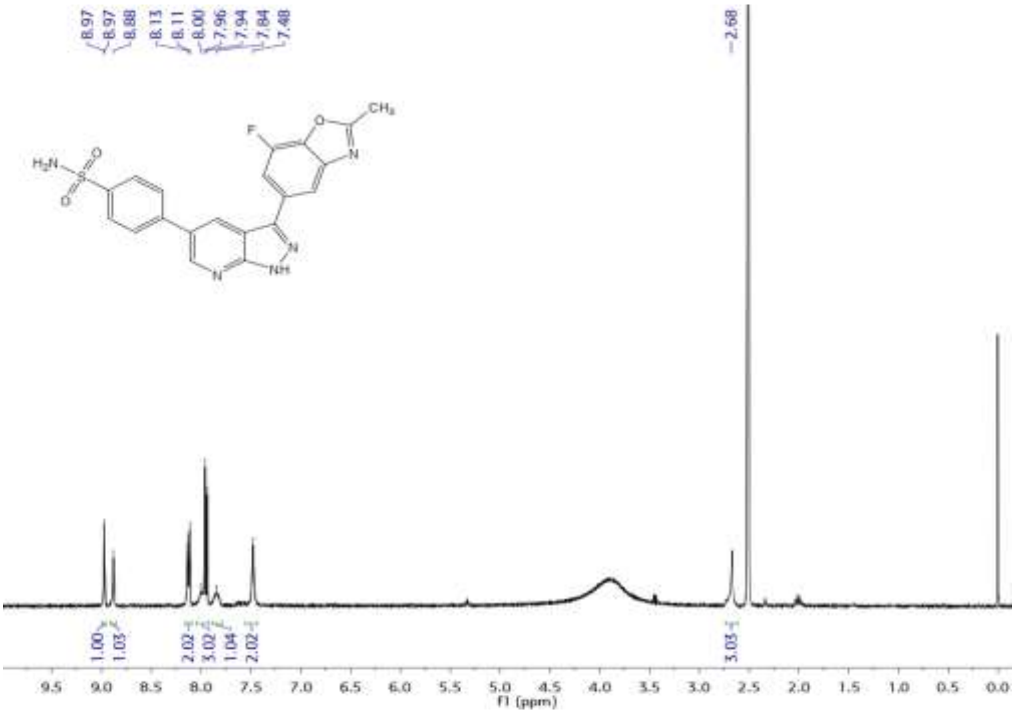

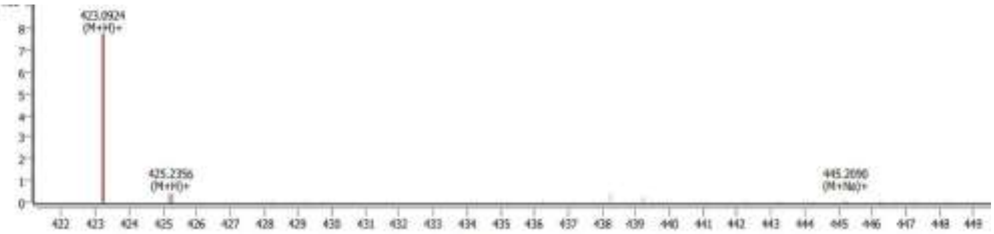

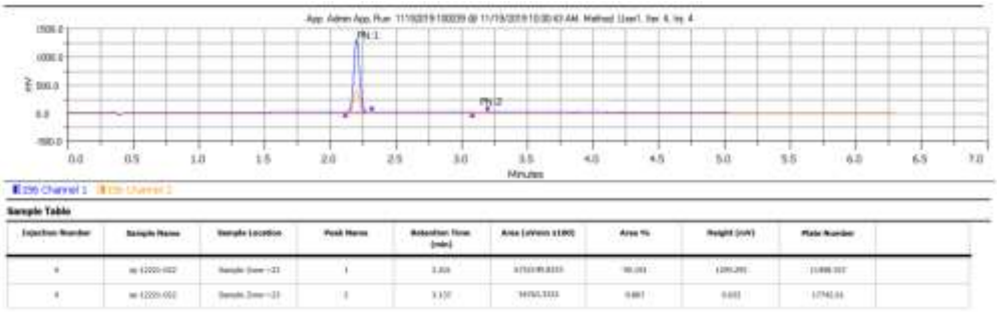

^{1}H NMR, ^{13}C NMR, HRMS and HPLC spectra of **15s**

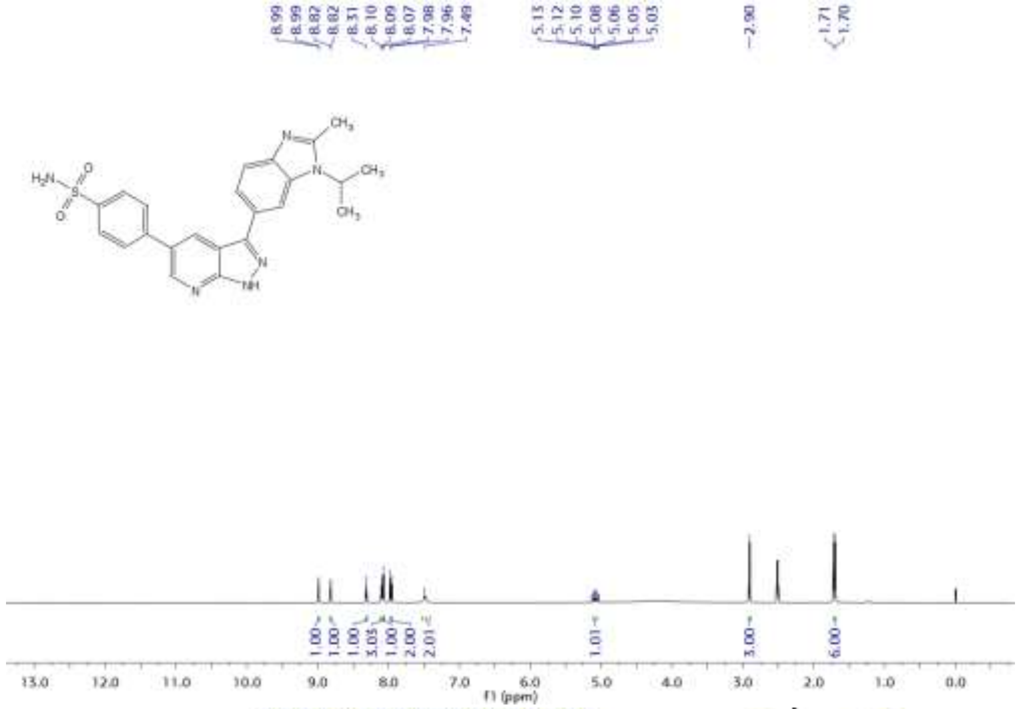

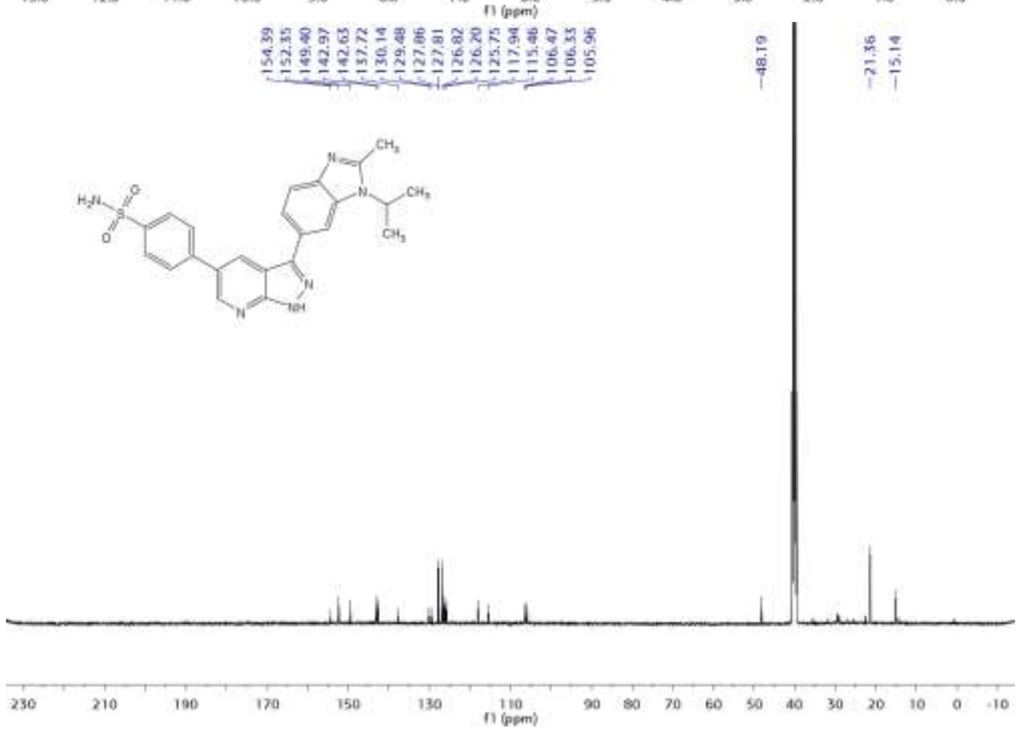

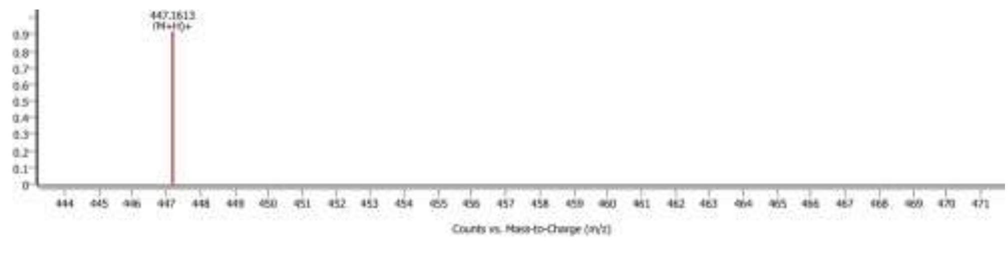

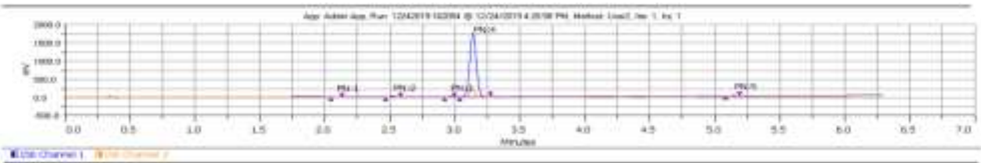

Sample Table

Injection Number	Sample Name	Sample Location	Peak Name	Retention Time (min)	Area (uVolts x1000	Area %	Height (mV)	Plate Number	
1	[illegible]	[illegible]	1	[illegible]	[illegible]	[illegible]	[illegible]	[illegible]	
1	[illegible]	[illegible]	2	[illegible]	[illegible]	[illegible]	[illegible]	[illegible]	
1	[illegible]	[illegible]	3	[illegible]	[illegible]	[illegible]	[illegible]	[illegible]	
1	[illegible]	[illegible]	4	[illegible]	[illegible]	[illegible]	[illegible]	[illegible]	
1	[illegible]	[illegible]	5	[illegible]	[illegible]	[illegible]	[illegible]	[illegible]	

^{1}H NMR, ^{13}C NMR, HRMS and HPLC spectra of **15t**

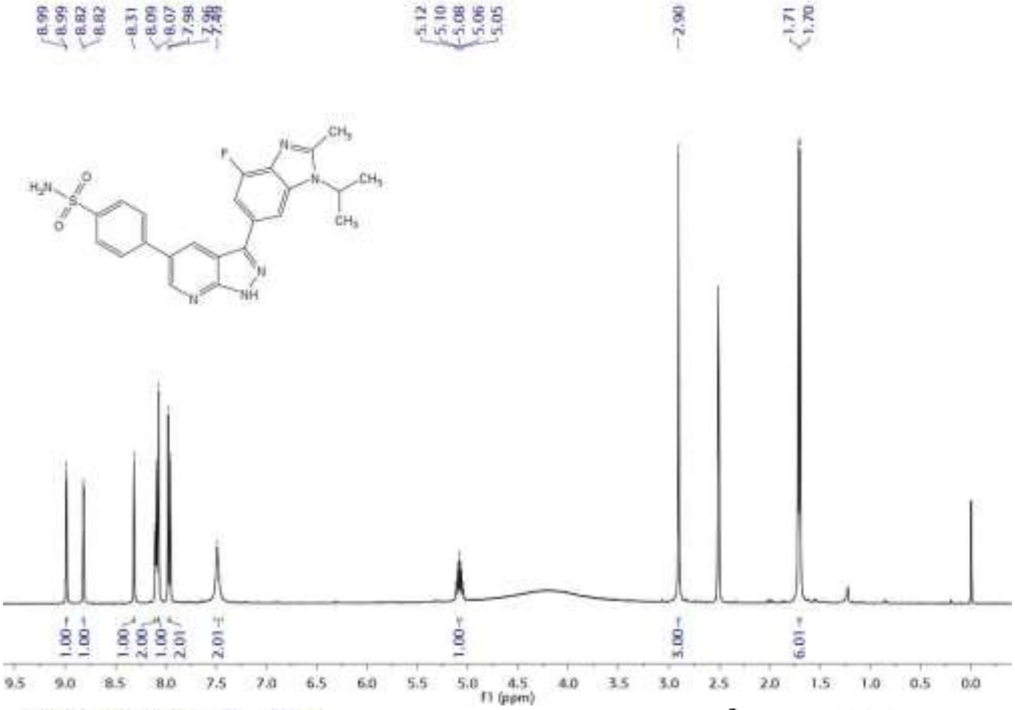

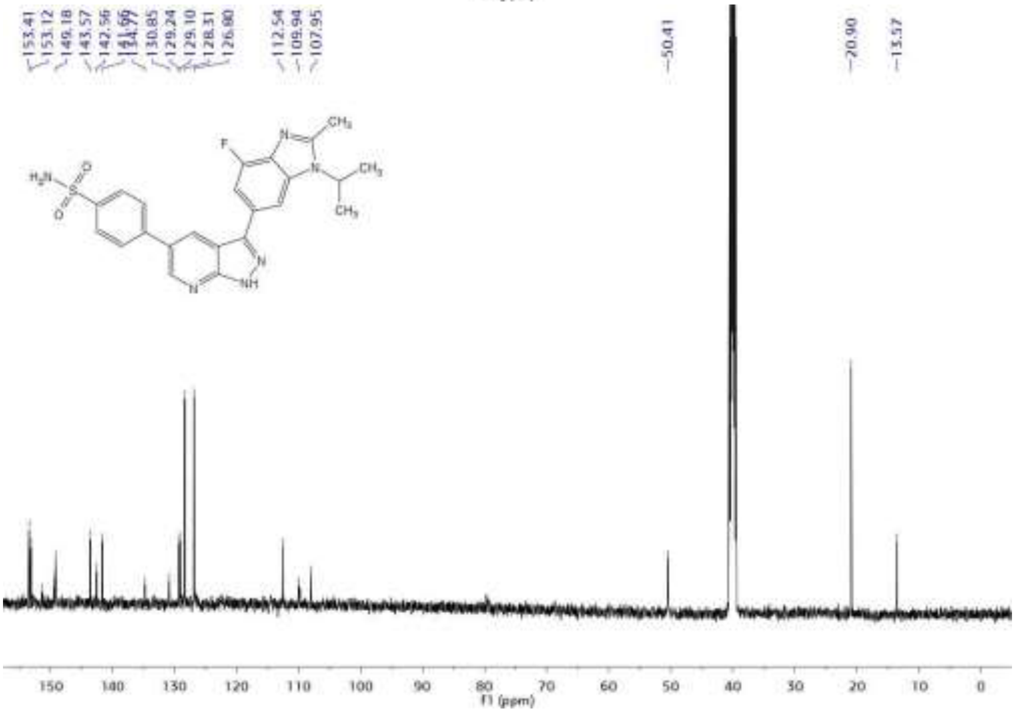

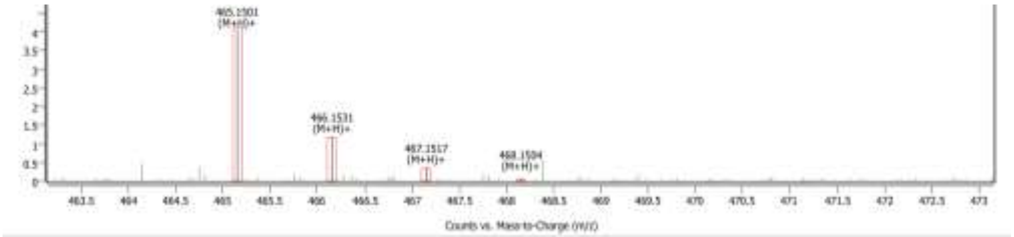

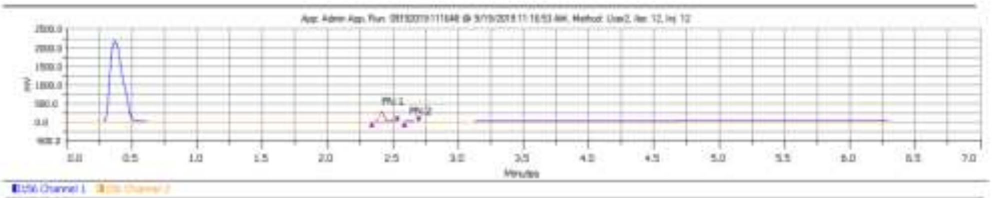

Sample Table

Injection Number	Sample Name	Sample Location	Peak Name	Retention Time (min)	Area (μV*min x1000)	Area %	Height (mV)	Plate Number	
[illegible]	[illegible]	[illegible]	1	[illegible]	[illegible]	[illegible]	[illegible]	[illegible]	
[illegible]	[illegible]	[illegible]	2	[illegible]	[illegible]	[illegible]	[illegible]	[illegible]	

^{1}H NMR, ^{13}C NMR, HRMS and HPLC spectra of **15u**

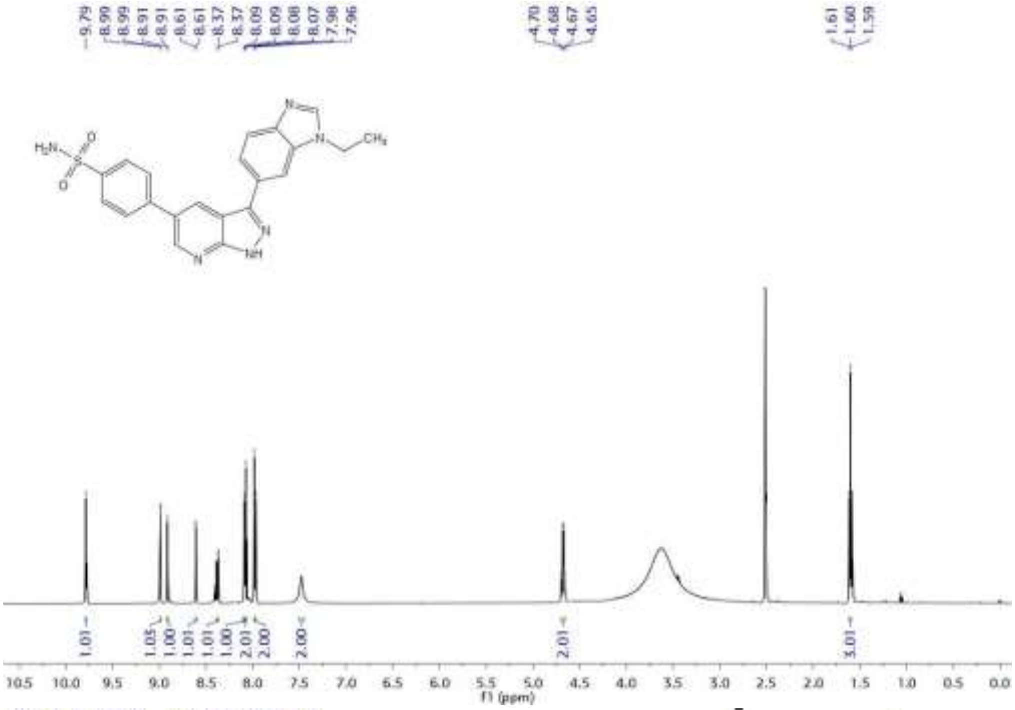

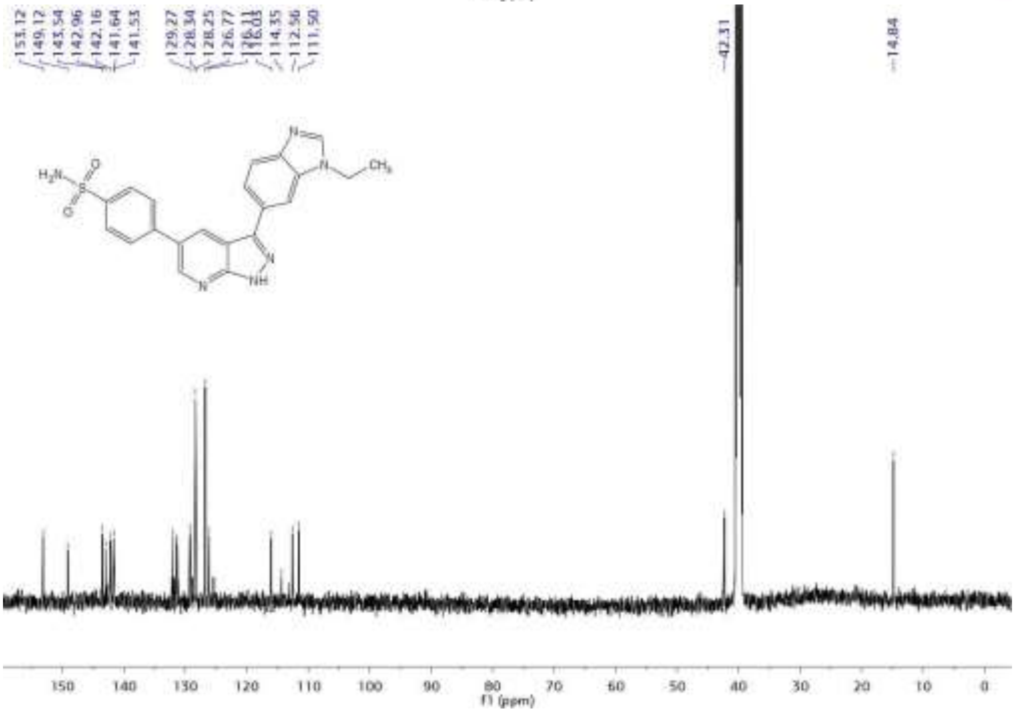

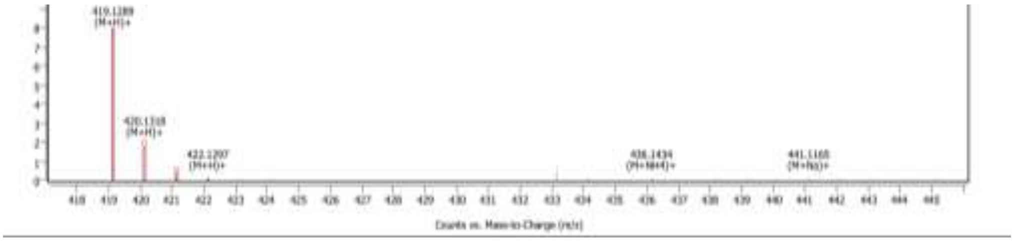

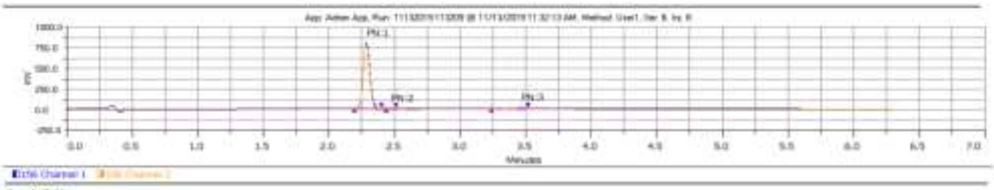

Sample Table

Injection Number	Sample Name	Sample Location	Peak Name	Retention Time (min)	Area (uV*min x100)	Area %	Height (mV)	Plate Number
[illegible]	[illegible]	[illegible]	1	[illegible]	[illegible]	[illegible]	[illegible]	[illegible]
[illegible]	[illegible]	[illegible]	2	[illegible]	[illegible]	[illegible]	[illegible]	[illegible]
[illegible]	[illegible]	[illegible]	3	[illegible]	[illegible]	[illegible]	[illegible]	[illegible]

^{1}H NMR, ^{13}C NMR, HRMS and HPLC spectra of **15v**

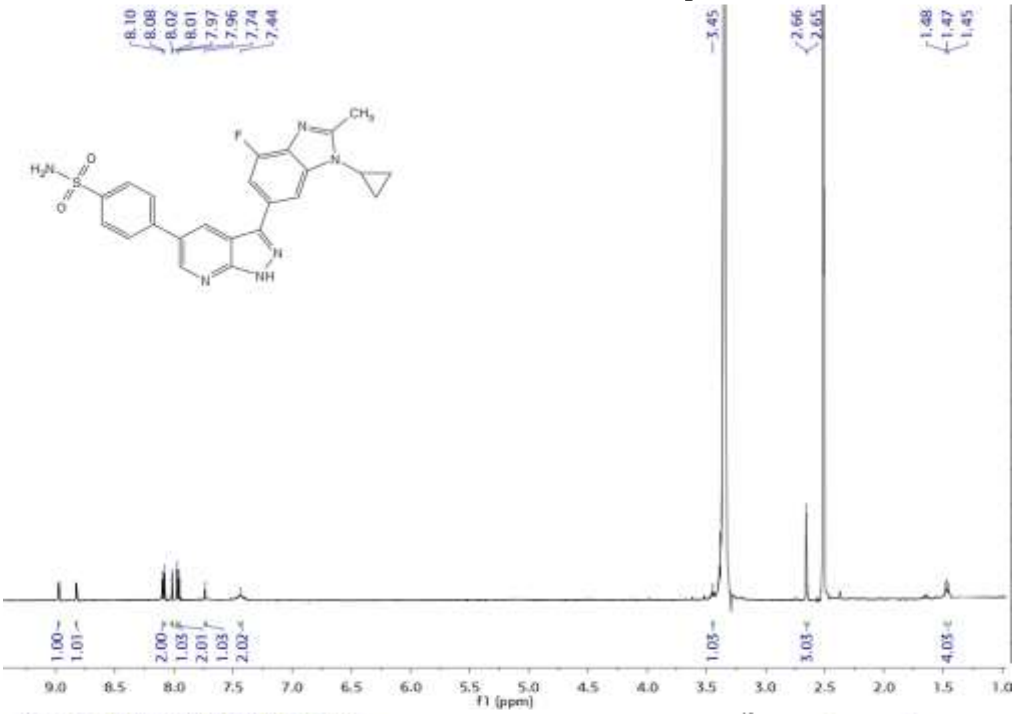

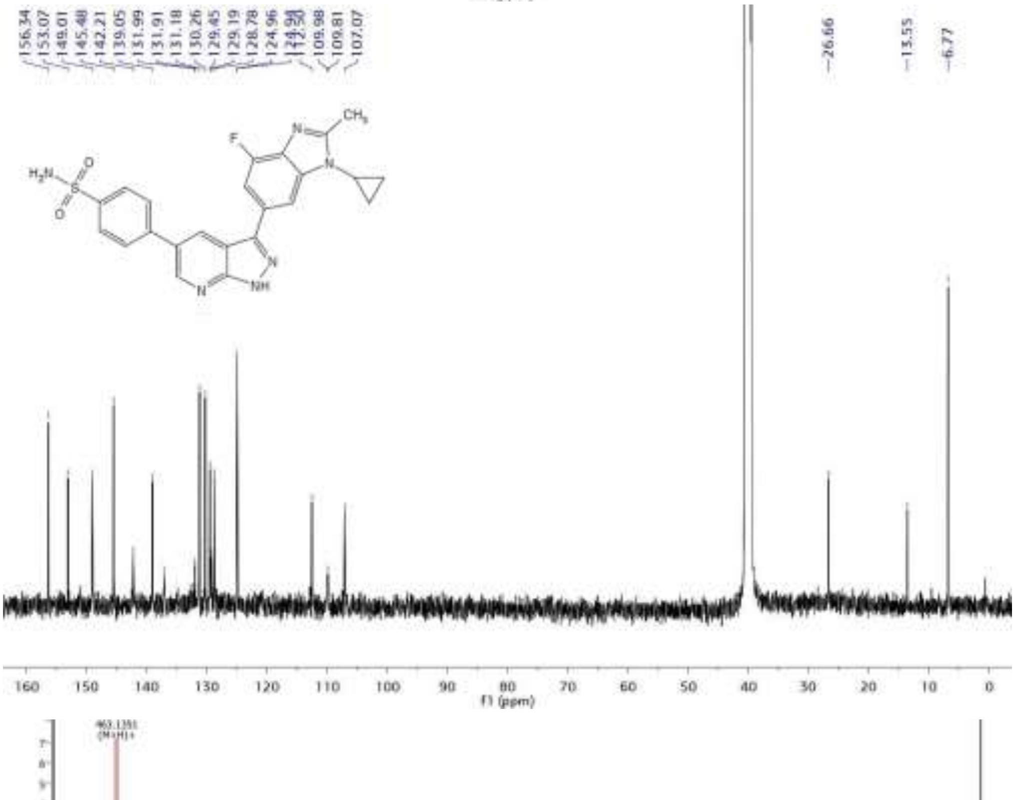

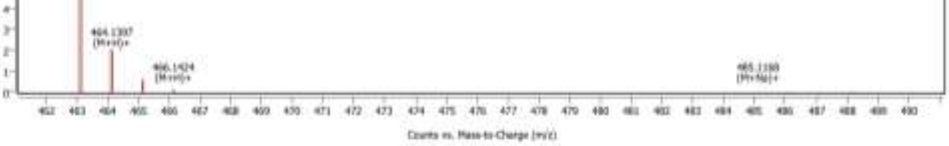

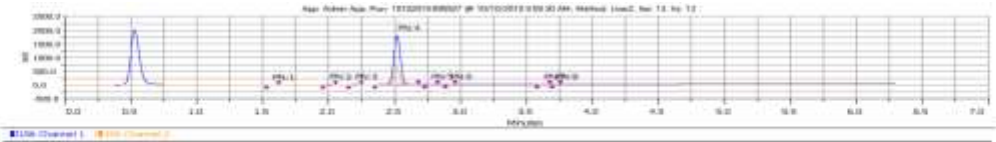

Sample Table

Injection Number	Sample Name	Sample Location	Peak Name	Retention Time (min)	Area [illegible]	Area %	Height [illegible]	Plate Number	
[illegible]	[illegible]	[illegible]	1	[illegible]	[illegible]	[illegible]	[illegible]	[illegible]	
[illegible]	[illegible]	[illegible]	2	[illegible]	[illegible]	[illegible]	[illegible]	[illegible]	
[illegible]	[illegible]	[illegible]	3	[illegible]	[illegible]	[illegible]	[illegible]	[illegible]	
[illegible]	[illegible]	[illegible]	4	[illegible]	[illegible]	[illegible]	[illegible]	[illegible]	
[illegible]	[illegible]	[illegible]	5	[illegible]	[illegible]	[illegible]	[illegible]	[illegible]	
[illegible]	[illegible]	[illegible]	6	[illegible]	[illegible]	[illegible]	[illegible]	[illegible]	
[illegible]	[illegible]	[illegible]	7	[illegible]	[illegible]	[illegible]	[illegible]	[illegible]	
[illegible]	[illegible]	[illegible]	8	[illegible]	[illegible]	[illegible]	[illegible]	[illegible]	

^{1}H NMR, ^{13}C NMR, HRMS and HPLC spectra of **15w**

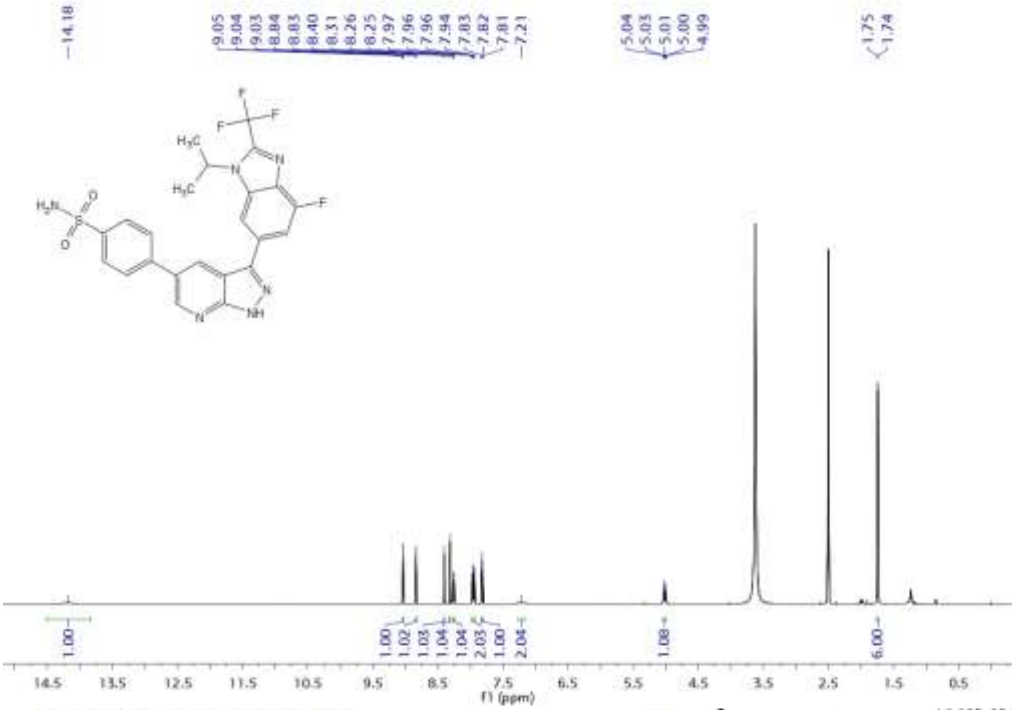

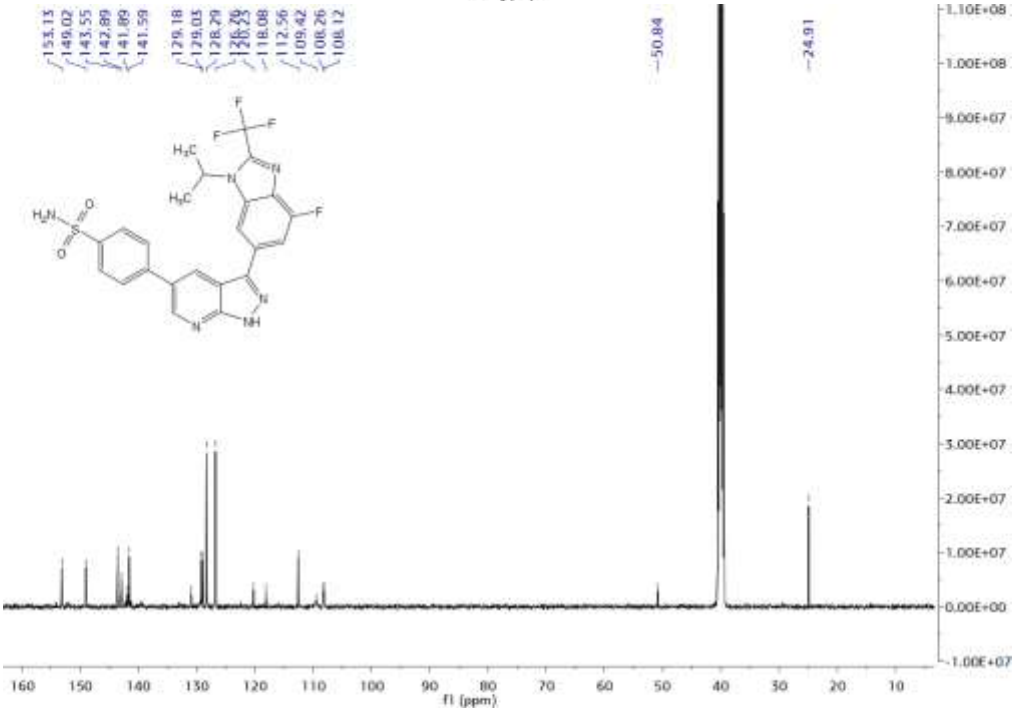

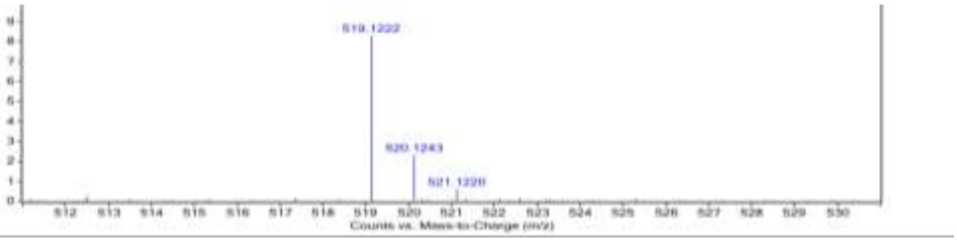

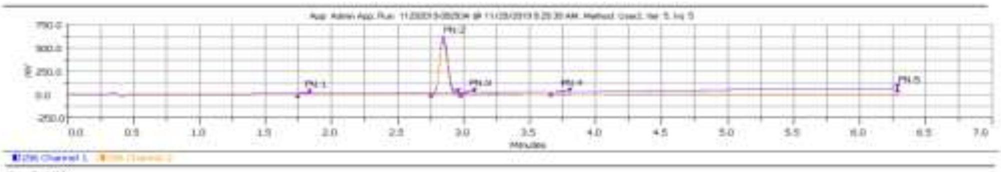

Sample Table

Injection Number	Sample Name	Sample Location	Peak Name	Retention Time (min)	Area (uV*min x1000)	Area %	Height (mV)	Plate Number	
[illegible]	[illegible]	[illegible]	1	[illegible]	[illegible]	[illegible]	[illegible]	[illegible]	
[illegible]	[illegible]	[illegible]	2	[illegible]	[illegible]	[illegible]	[illegible]	[illegible]	
[illegible]	[illegible]	[illegible]	3	[illegible]	[illegible]	[illegible]	[illegible]	[illegible]	
[illegible]	[illegible]	[illegible]	4	[illegible]	[illegible]	[illegible]	[illegible]	[illegible]	
[illegible]	[illegible]	[illegible]	5	[illegible]	[illegible]	0	[illegible]	[illegible]	

^{1}H NMR, ^{13}C NMR, HRMS and HPLC spectra of **15x**

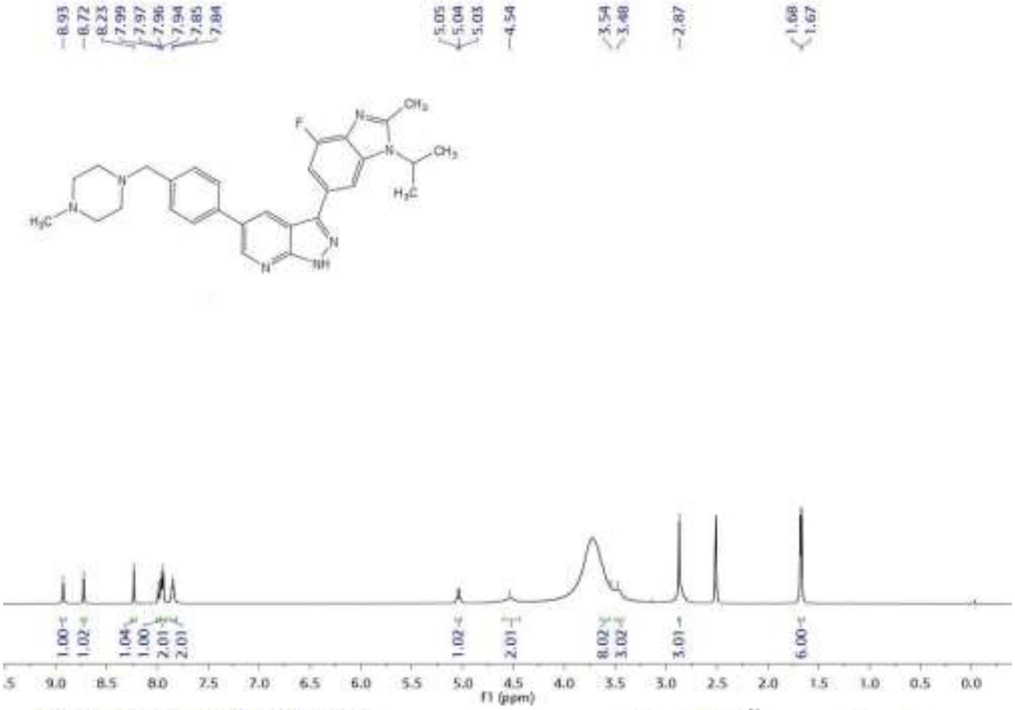

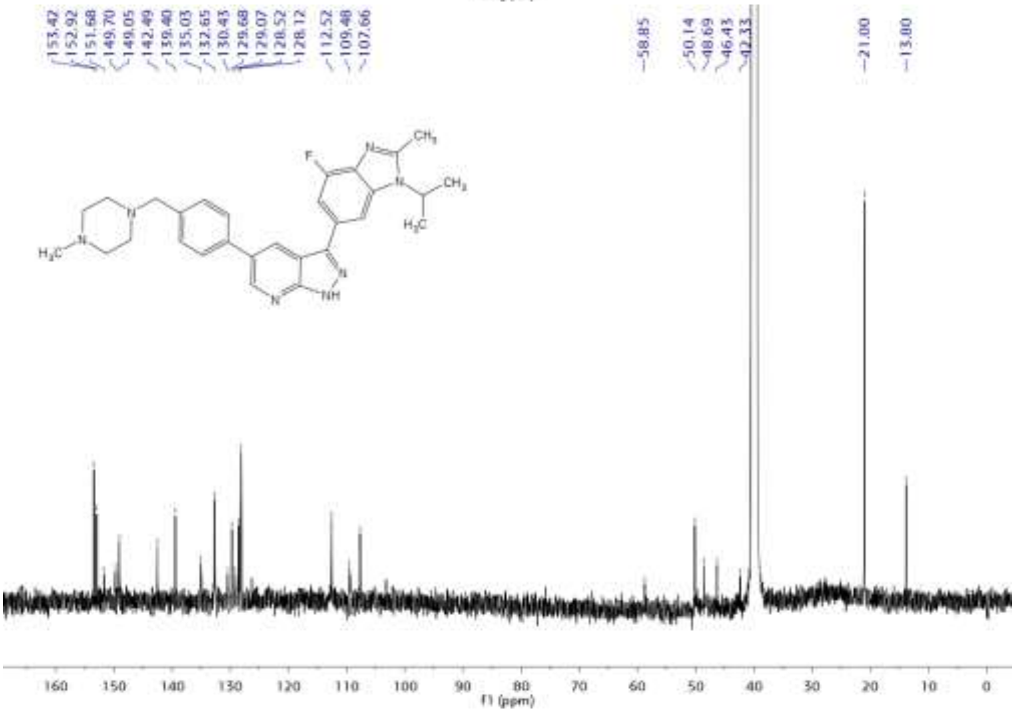

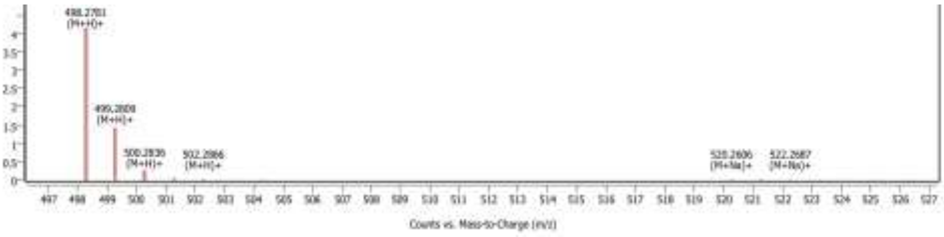

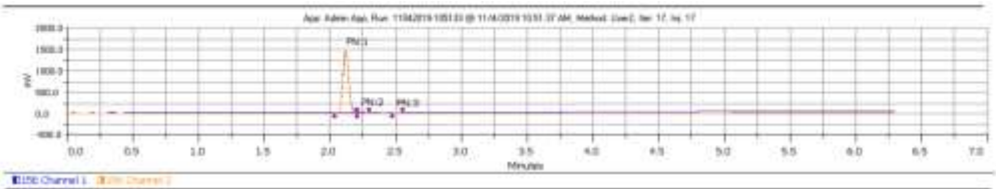

Sample Table

Injection Number	Sample Name	Sample Location	Peak Name	Retention Time (min)	Area ([illegible])	Area %	Height ([illegible])	Plate Number	
17	[illegible]	[illegible]	1	[illegible]	[illegible]	[illegible]	[illegible]	[illegible]	
17	[illegible]	[illegible]	[illegible]	[illegible]	[illegible]	[illegible]	[illegible]	[illegible]	
17	[illegible]	[illegible]	[illegible]	[illegible]	[illegible]	[illegible]	[illegible]	[illegible]	

^{1}H NMR, ^{13}C NMR, HRMS, LRMS and HPLC spectra of **15y**

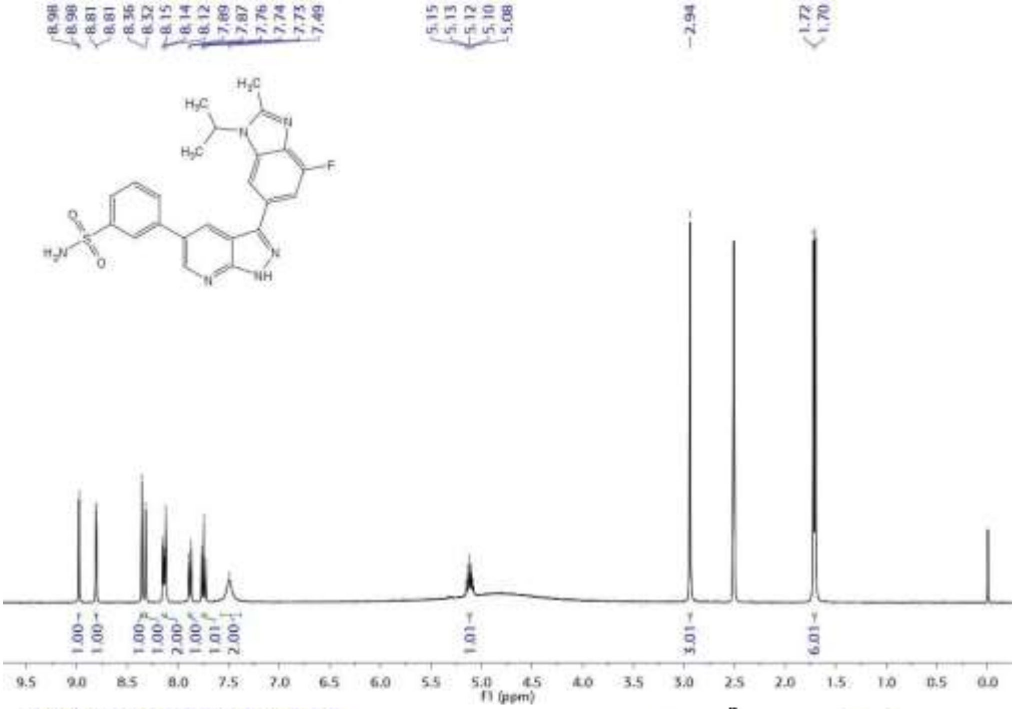

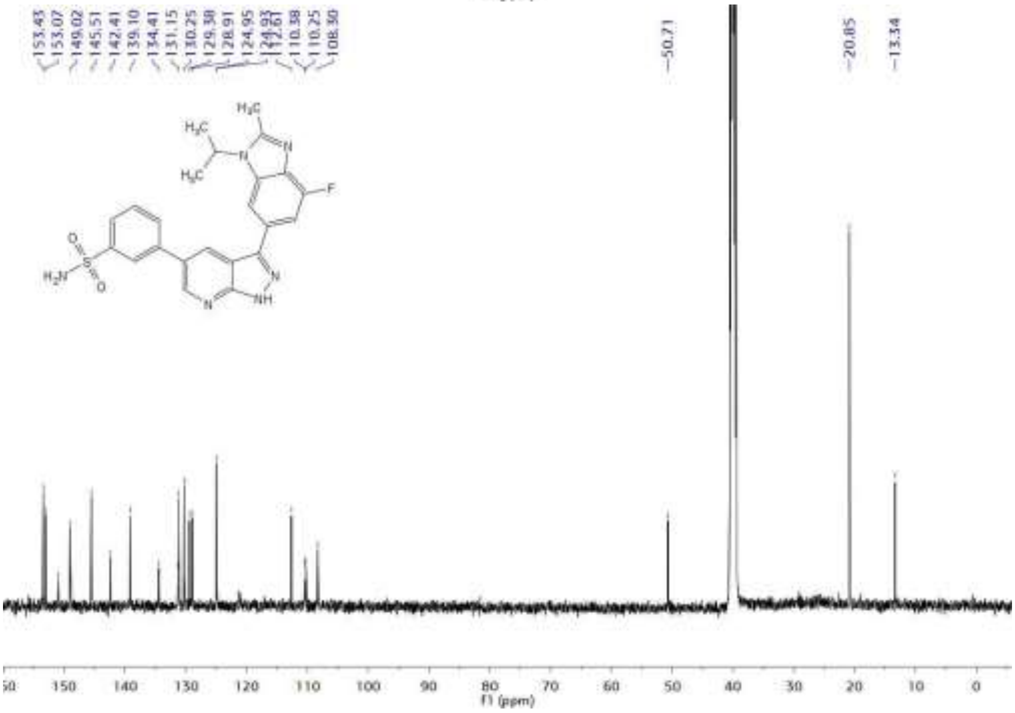

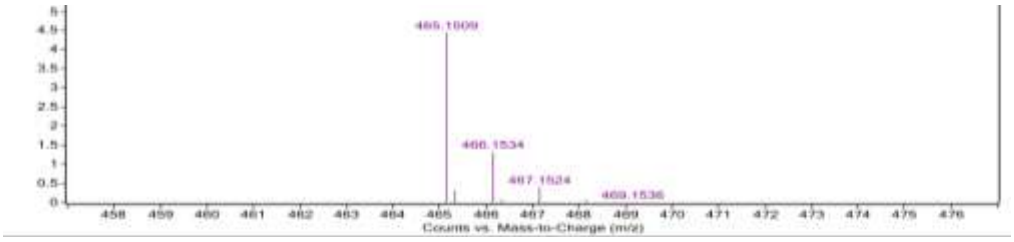

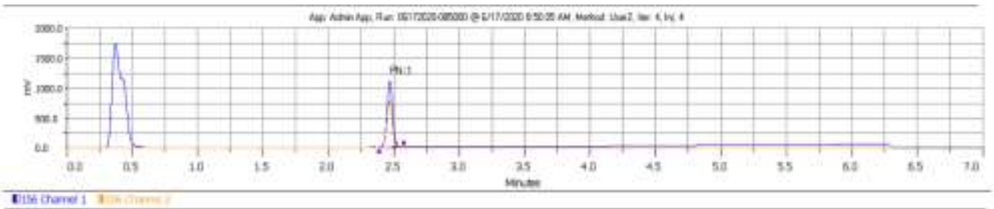

Sample Table

Injection Number	Sample Name	Sample Location	Peak Name	Retention Time (min)	Area [illegible]	Area %	Height (mV)	Plate Number	
4	[illegible]	[illegible]	1	2.471	[illegible]	100	[illegible]	[illegible]	

^{1}H NMR, ^{13}C NMR, HRMS and HPLC spectra of **15z**

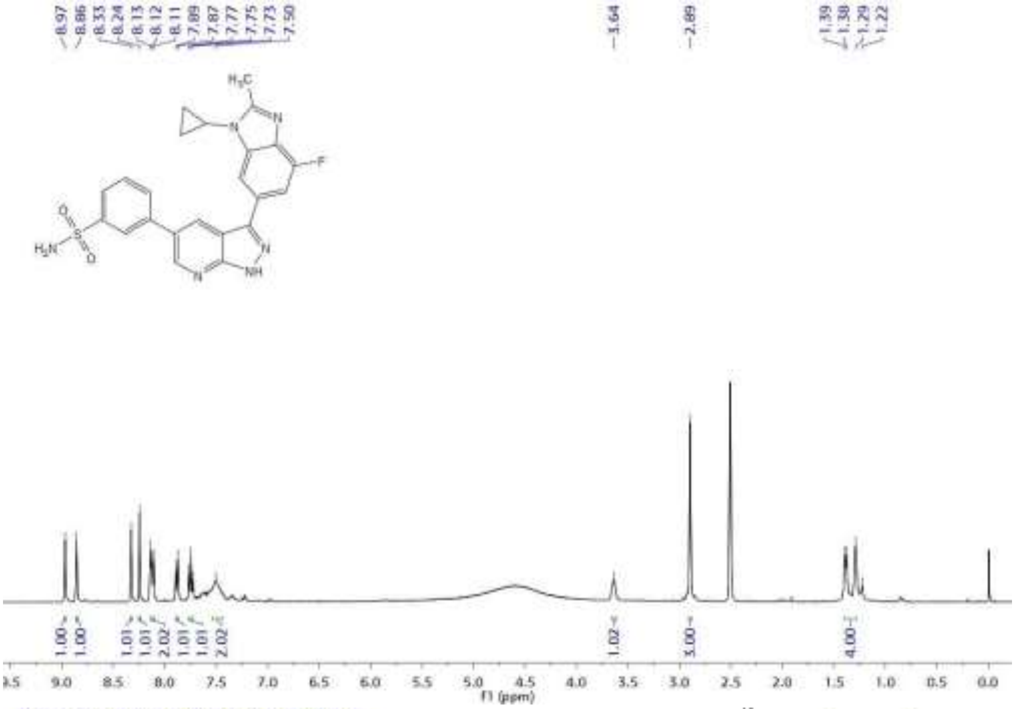

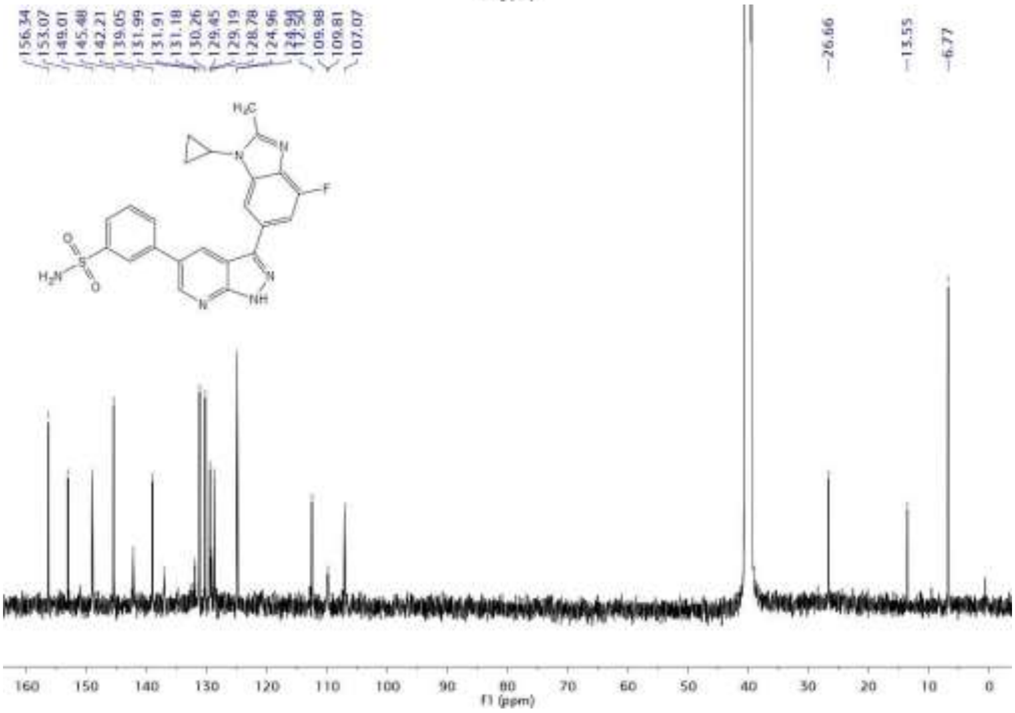

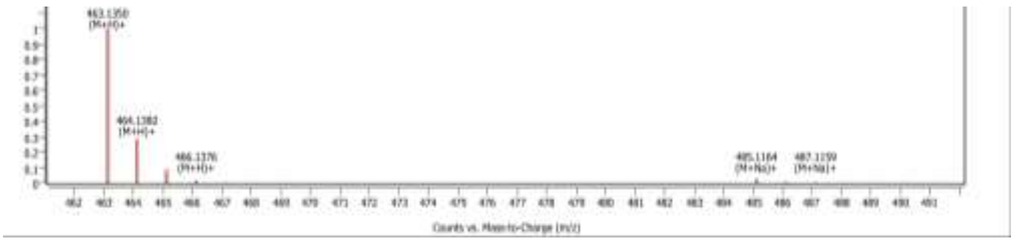

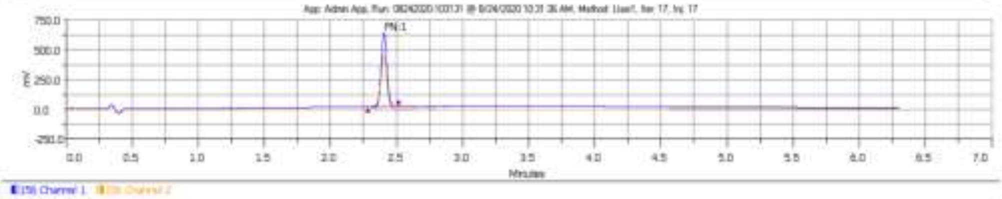

Sample Table

Injection Number	Sample Name	Sample Location	Peak Name	Retention Time (min)	Area (uV*min x100)	Area %	Height (mV)	Plate Number	
17	sy-12221-657-h	Sample Zone->13	1	2.408	3176440.8262	100	628.825	14505.098	

^{1}H NMR, ^{13}C NMR, HRMS and HPLC spectra of **15aa**

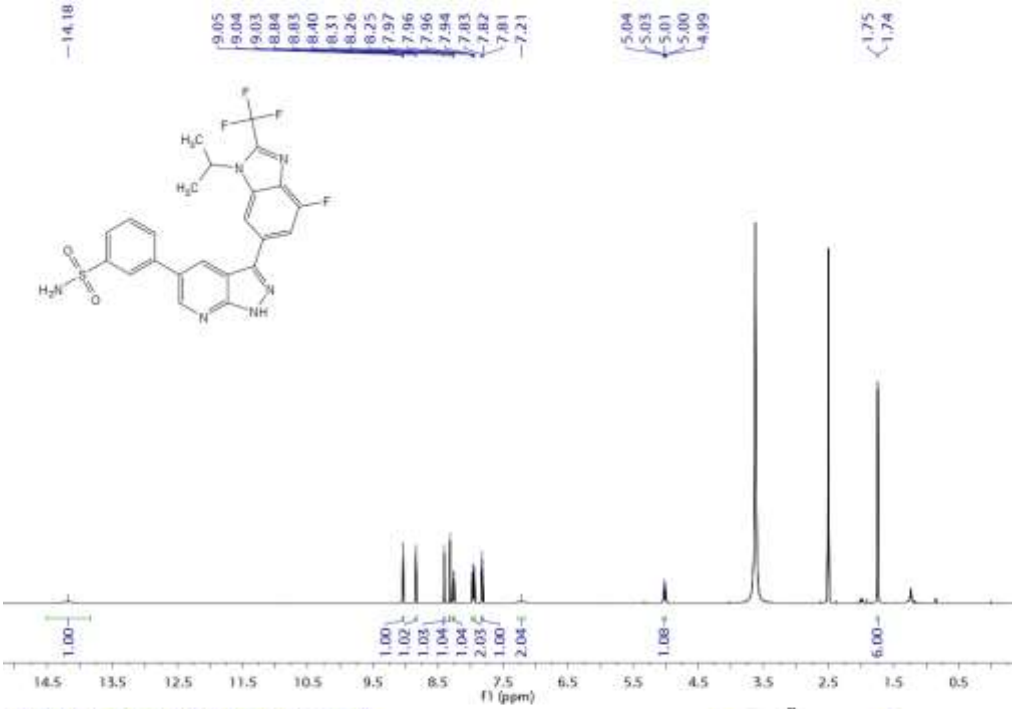

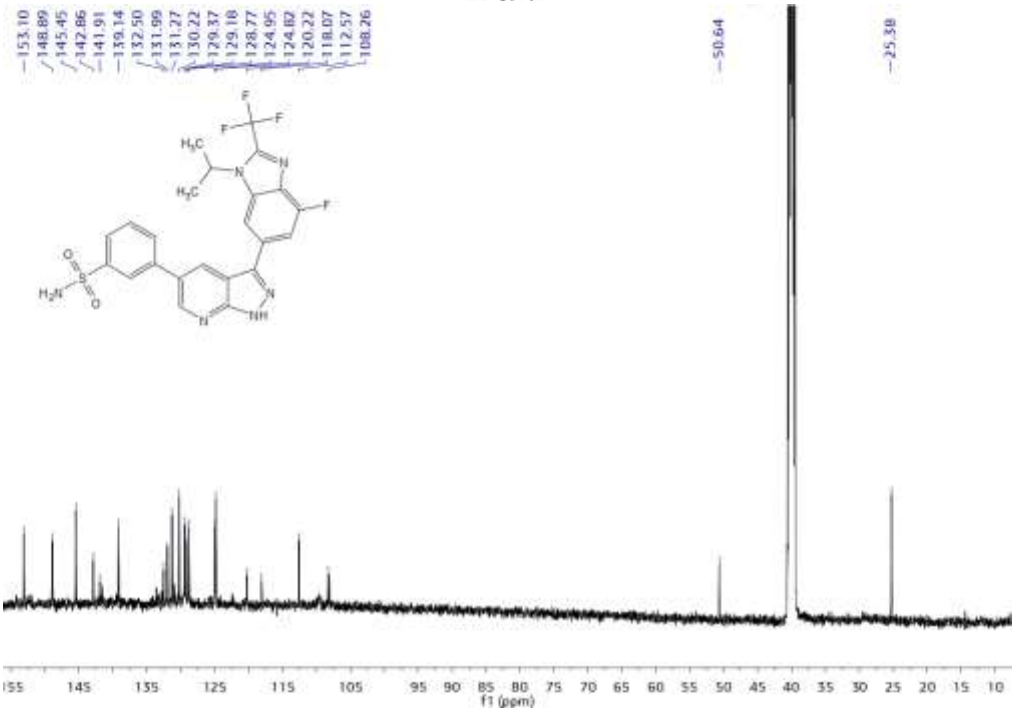

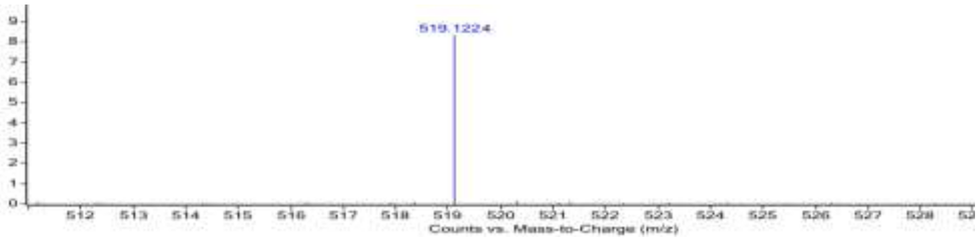

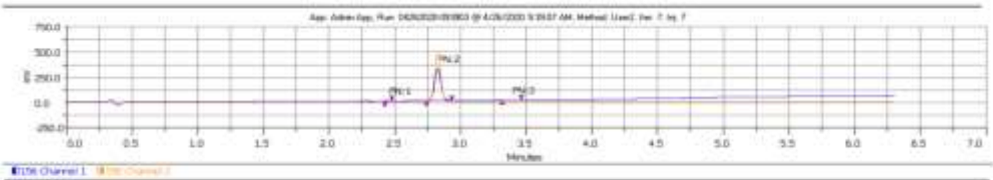

Sample Table

Injection Number	Sample Name	Sample Location	Peak Name	Retention Time (min)	Area [illegible]	Area %	Height [illegible]	Plate Number
7	[illegible]	Sample Zone [illegible]	1	2.451	[illegible]	[illegible]	[illegible]	[illegible]
7	[illegible]	Sample Zone [illegible]	2	2.83	[illegible]	98.138	[illegible]	[illegible]
7	[illegible]	Sample Zone [illegible]	3	[illegible]	[illegible]	1.464	[illegible]	[illegible]

^{1}H NMR, ^{13}C NMR, HRMS and HPLC spectra of **15ab**

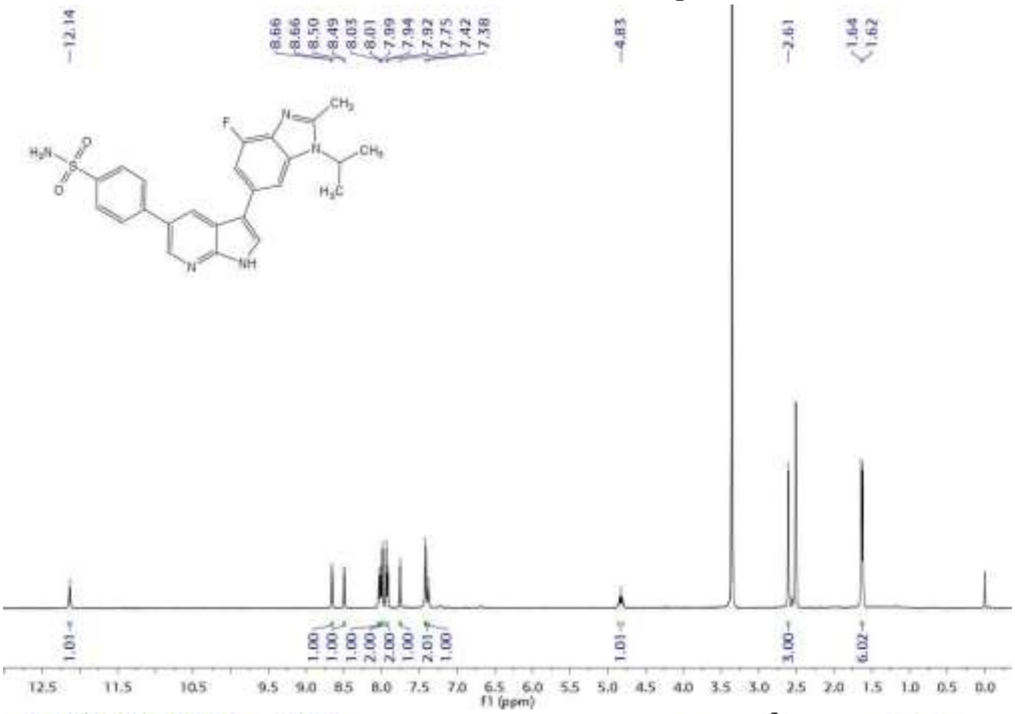

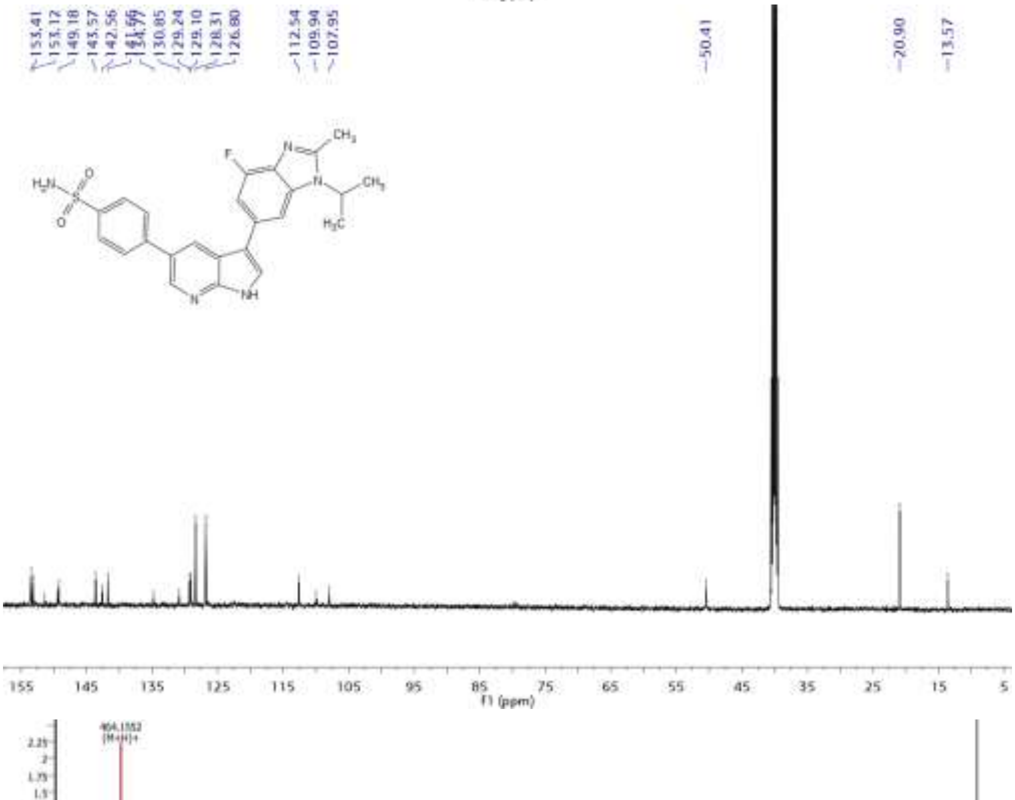

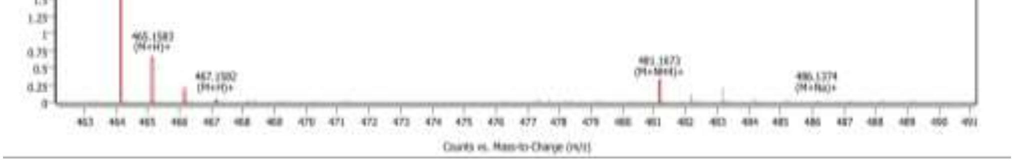

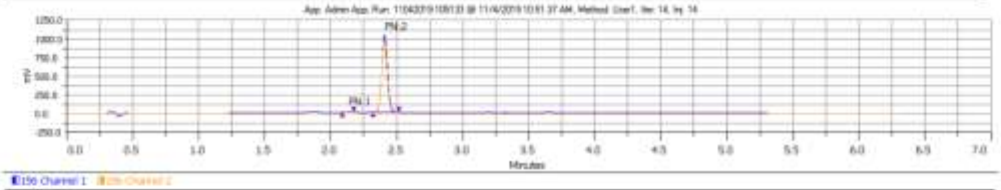

Sample Table

Injection Number	Sample Name	Sample Location	Peak Name	Retention Time (min)	Area [illegible]	Area %	Height (mV)	Plate Number
14	[illegible]	[illegible]	1	[illegible]	[illegible]	[illegible]	[illegible]	[illegible]
14	[illegible]	[illegible]	2	2.41	[illegible]	[illegible]	[illegible]	[illegible]
